# A Reinterpretation of the Imidazolate Au(I) Cyclic Trinuclear Compounds Reactivity with Iodine and Methyl Iodide with the Perspective of the Inverted Ligand Field Theory

**DOI:** 10.1021/acs.inorgchem.1c03492

**Published:** 2022-02-15

**Authors:** Rossana Galassi, Lorenzo Luciani, Claudia Graiff, Gabriele Manca

**Affiliations:** † School of Science and Technology, Chemistry Division, University of Camerino, Via Sant’Agostino, 1, I-62032, Camerino, Italy; ‡ Department of Chemistry, Life Sciences and Environmental Sustainability, Università degli Studi di Parma, Parco Area delle Scienze 17/A, 43124 Parma, Italy; § Istituto di Chimica dei Composti Organo-Metallici, CNR-ICCOM, 50019, Sesto Fiorentino, Italy

## Abstract

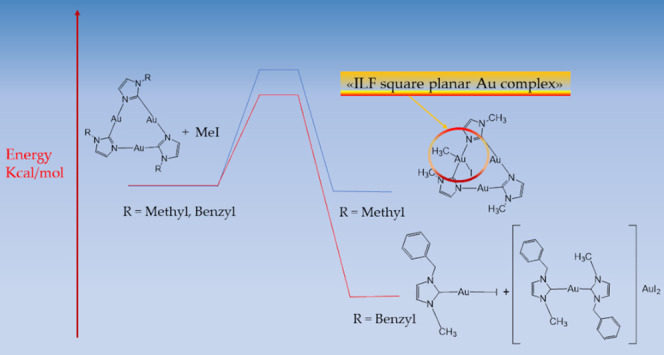

Coinage metal cyclic trinuclear compounds (CTCs) are an emerging class of metal coordination compounds that are valuable for many fine optoelectronic applications, even though the reactivity dependence by the different bridging ligands remains somewhat unclear. In this work, to furnish some hints to unravel the effect of substituents on the chemistry of Au(I) CTCs made of a specific class of bridging ligand, we have considered two imidazolate Au(I) CTCs and the effect of different substituents on the pyrrolic N atoms relative to classic metal oxidations with I_2_ or by probing electrophilic additions. Experimental suggestions depict a thin borderline between the addition of MeI to the N-methyl or N-benzyl imidazolyl CTCs, which afford the oxidized CTC in the former and the ring opening of the CTC and the formation of carbene species in the latter. Moreover, the reactions with iodine yield to the oxidation of the metal centers for the former and just of a metal center in the latter, even in molar excess of iodine. The analysis of the bond distances in the X-ray crystal structures of the oxidized highlights that Au(III)-C and Au(III)-N bonds are longer than observed for Au(I)–C and Au(I)–N bonds, as formally not expected for Au(III) centers. Computational studies converge on the attribution of these discrepancies to an additional case of inverted ligand field (ILF), which solves the question with a new interpretation of the Au(I)–ligand bonding in the oxidized CTCs, which furnishes a new interpretation of the Au(I)-ligand bonding in the oxidized CTCs, opening a discussion about addition/oxidation reactions. Finally, the theoretical studies outputs depict energy profiles that are compatible with the experimental results obtained in the reaction of the two CTCs toward the addition of I_2_, MeI, and HCl.

## Abstract 2

A revisitation of some classic oxidation reactions of gold centers in cyclic trinuclear compounds (CTCs) provides experimental results leading to the opportunity to delineate the effect of imidazole substituents in different outcomes from the reactions of CTCs with I_2_ or MeI. Moreover, with the match between experimental and theoretical results, a new interpretation of the oxidation states of tetracoordinate gold as cases of inverted ligand field (ILF) is discussed.

## Introduction

Cyclic trinuclear complexes (CTCs) containing triangular d^10^ metal frame may be obtained with a series of angular ditopic anionic bridging ligands in combination with linearly coordinated M(I) cations from Group 11 elements; hence, ligands as pyrazolate (Pz), imidazolate (Im), 1,2,4-triazolate (Trz), pyridinate (Py), and carbeniate (Cb) have been employed to obtain this fascinating class of compounds.^1,2^ The attention toward these compounds was earlier focused on their elegant structures, which were later discovered to be associated with multipurpose and functional emissive properties.^3,4^ These observations were accompanied by the study of unique chemical properties due, for example, to π-acid/π-base features promoting them as potential materials for sensing, molecular recognition, in general, for optoelectronics application.^4,5^

**Scheme 1:**
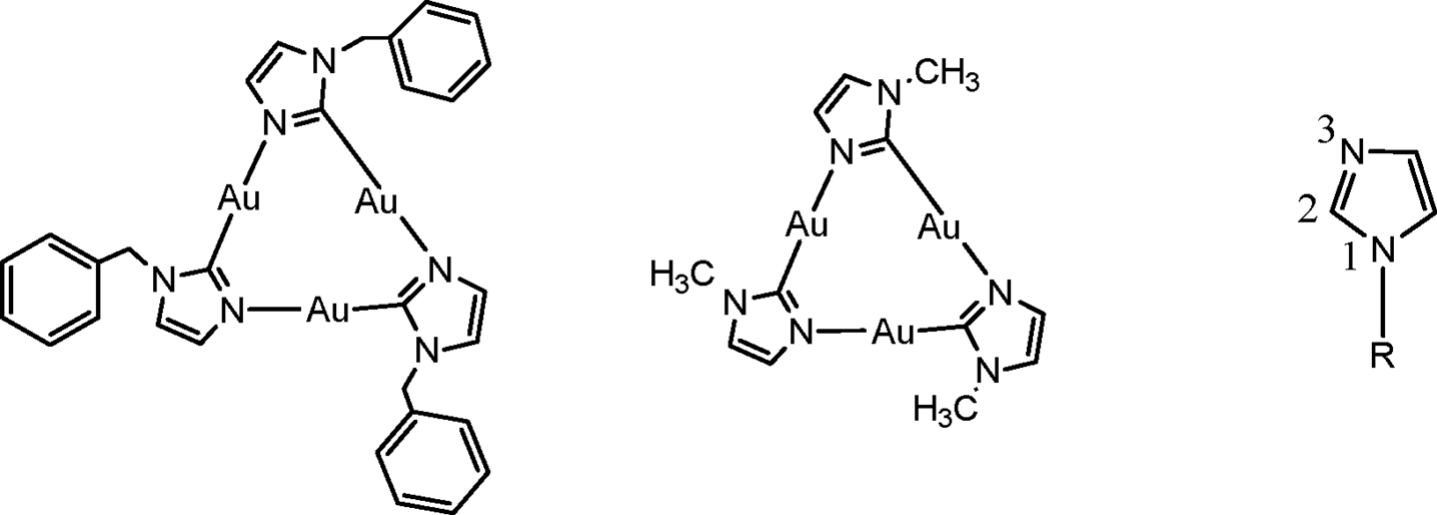
Au(I) CTCs with the Labeling of the Imidazole Atoms Quoted in the Manuscript

Theoretical studies have shown that both the nature of the central metal and the ligand, as well as of the substituents on the ligands, modulates the acid–base properties of the CTCs, as well as their photophysical characteristics. For example, CTCs with carbeniates and imidazolates as ligands and Au(I) centers as metals feature superior π-basic properties.^6^ Therefore, gold(I) carbeniate or imidazolate CTCs, after their first preparation in the early 1970s^7^ and 1980s^8^ have been reconsidered for novel purposes as the preparation of stacked supramolecular compounds with electrophiles such as metal cations,^9^ π-acid organic molecules,^10,11^ or for the formulation of mixed metal Au/Ag or Au/Cu CTCs, whose peculiar emissive properties are strongly dependent on the heteronuclear M–M′ intertrimer interactions.^12,13^ In this latter work, the reaction between CTCs consisting of imidazole ligands with different alkyl or aryl substituents and Au(I) with a Cu(I) CTC, the [Cu-μ-N,N-3,5-(CF_3_)_2_-pyrazolate]_3_, allowed the formation of mixed-metal CTCs, consisting of Au_2_Cu or Cu_2_Au arrangements, depending on the stoichiometry of the reactions. These compounds are obtained with very high yields and they exhibit extraordinary stability in the solid state and strongly emission properties with quantum yields close to unity.^13^ By considering the acid/base scale build up according to theoretical calculations made by Tekarli,^6^ the imidazole-based Au(I) CTCs are π-basic while the Cu pyrazole based CTC is π-acid; therefore, the different outcomes of these reactions, yielding π–π adducts or mixed metal CTCs, highlighted that the mechanism is likely influenced by both the electronic and steric effects of the substituents on the imidazolate ligand.^13^ At a first glance, the steric effects may be considered as the key point but, to gain a deeper insight into the role of the substituents, additional studies are herein performed to highlight the reactivity of the gold centers belonging to the two different CTCs toward common oxidants. Therefore, the reactions of the [μ-C,N-1-methylimidazolate-Au]_3_, CTC^Me^, or [μ-C,N-1-benzylimidazolate-Au]_3_, CTC^Bz^, with methyl iodide (MeI) and iodine (I_2_) were led. The reactions with halogens or MeI are a well-known method to analyze the tendency of metal centers to be oxidized. Iodine was already used as an oxidant for carbeniate and imidazolate Au(I) CTCs, where a stepwise iodine addition at the three Au(I) centers was observed for the former CTC^14,15^ but only the addition to one Au(I) center was reached for the benzylimidazolate system.^16^ On the other hand, the addition of MeI has been never attempted for this class of compounds. Iodine and MeI are different oxidants: Iodine, contrary to the other halogens, does not oxidize directly AuI to AuI_3_ (redox potential +1.41 V) but it requires a linear LAuI precursor (where L is a phosphane or a carbene);^17,18^ on the other hand, MeI is reported to activate the oxidation of Au(I) to Au(II) species in dinuclear compounds.^19,20^ In this work, to grasp the effect of N_1_-imidazole substituents on the reactivity of Au(I) CTCs, the addition of two classic oxidants as MeI or I_2_ to the [μ-C,N-1-methylimidazolate-Au]_3_ (labeled as CTC^Me^) and to [μ-C,N-1-benzylimidazolate-Au]_3_ (labeled as CTC^Bz^) compounds were performed (see Scheme 1). The experimental results were compared to the ones reported in the literature and with the data obtained by computational methods.

The combined experimental/computational analysis revealed the different reactivity of this class of Au(I) CTC with the alternative substrates. In particular, the work not only highlights the non-innocent behavior of the imidazolate ligand but also reverts the electronic description of the formally depicted “Au(III)” square planar complexes by introducing the inverted ligand field (ILF) concept. In this view, a detailed analysis of the electronic structure revealed that the metal maintains the original d^10^ along the overall reaction pathways.

## Results and Discussion

Au(I) cyclotrimers are a class of cyclic gold compounds where a triangular array of Au ions are linked by C,N or N,N bridging ligands. Following the reactions of different cyclotrimers, CTCs, with iodine and MeI are presented and discussed.

### The Reactions of CTC^Me^ and CTC^Bz^ with Iodine

The reaction of CTC^Me^ with an oxidizing agent as iodine was performed with a molar excess of iodine in CH_2_Cl_2_, according to the method already used for CTC^Bz^.^16^ The evaporation of the reaction mixture yielded a tarry solid, soluble in hot tetrahydrofuran (THF); upon cooling the THF solution, orange-red needles and a few red platelet crystals were obtained, but only the latter were suitable for X-ray crystal diffraction. The elemental analysis of the crystalline solid roughly corresponds to the fully oxidized CTC^Me^I_6_, but the separation of the two types of crystals allowed the characterization of both components. The most abundant crystals are the needles and, according to the elemental analysis, were characterized as the fully oxidized CTC^Me^I_6_, compound **1**, while the platelets match with the partially oxidized CTC^Me^I_4_ • THF, where only two Au centers are bound to I atoms (compound **2**). The MIR and FIR spectra of compounds **1** and **2** exhibit mild differences in intensity and energies (see Figures S1 and S2 in the Supporting Information), while the characterizations of compounds **1** and **2** in solution were complicated by their low solubility in organic solvents. Compound **1** upon dissolution affords to compound **2** in a large extent (see Figures S3 and S4 in the Supporting Information). This latter exhibits a ^1^H NMR spectrum with six doublets for the imidazole ligands (ranging between 7.66 and 7.07 ppm) and three signals for the N-methyl groups (3.84, 3.69, and 3.67 ppm). In the ^13^C NMR spectrum, only one signal was observed for the C2 of the imidazoles at 166.09 ppm, likely for the low intensity of the signals (see Figures S5 and S6 in the Supporting Information); interestingly, these ^13^C signals differ by <1 ppm from that of the C2 of the starting CTC, found at 167 ppm (see Figure S7 in the Supporting Information). The single-crystal X-ray diffraction (XRD) of the crystal platelets revealed a structure made of the CTC^Me^ unit with two Au centers oxidized by the iodine and a molecule of THF.^15^ On the other hand, in the case of the reaction of CTC^Bz^ with iodine, a very soluble product was obtained in good yield, where only one Au center was bound with the I atoms.^16^

### Computational Studies on the Reaction of CTC^Me^ and CTC^Bz^ with Iodine

Although the addition of iodine is a well-known reaction with CTCs, a detailed analysis of the mechanism, an overall energetic/electronic rationalization of the full or partial oxidation of the metal centers, has never been provided. To fill this gap, theoretical calculations defined the mechanism involved in the addition of halogens to these systems and provided some useful hints on the evolution of the electronic structure during the reactivity.

#### Halogen Molecule Activation

**Figure 1:**
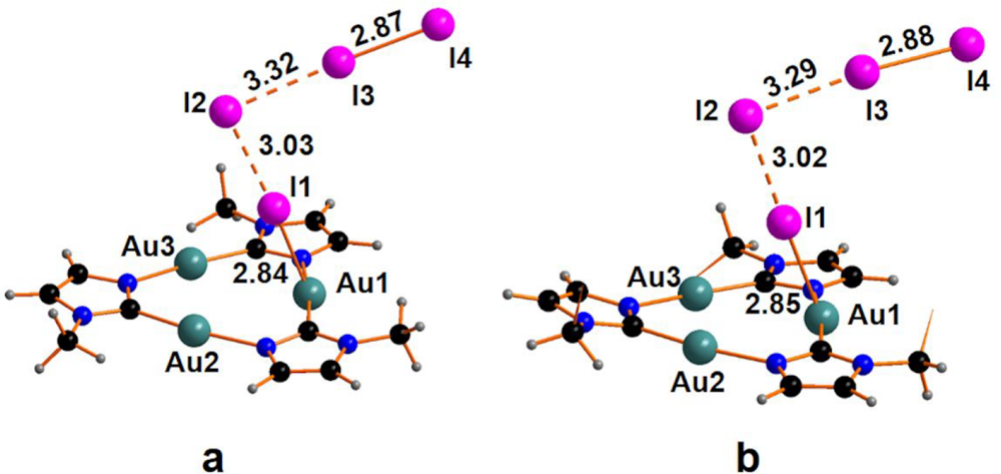
Optimized structure of the adducts: (a) CTC^Me^*2I_2_and (b) CTC^Bz^*2I_2_. The phenyl rings in panel (b) are hidden for the sake of clarity.

The halogen molecule activation by Au(I) complexes have been already addressed in the literature and, in principle, the process is generally accompanied by the formation of a square planar complex with the coordination number around the metal augmented by two units for the formation of two new metal–I linkages.^14−18,21^ This, in principle, allows the oxidation of the metal center; thus, the gold achieves a formal oxidation state of +III, two units more than the starting +I, with the two halogen atoms being reduced to halides. To better understand the reactivity of the starting CTCs with diiodine, two molecules of the substrate have been taken into account given the potential involvement of triiodide anions.^22^ Thus, the first step of the computational analysis was the *in silico* isolation of an initial adduct between the trinuclear species, namely, CTC^Me^ or CTC^Bz^ with the alternative methyl and benzyl substituents at the imidazole rings, and two I_2_ molecules. In both cases, the formation of the two initial adducts CTC^Me^*2I_2_ and CTC^Bz^*2I_2_, shown in Figures 1a and 1b, is exergonic by −11.8 and −15.1 kcal mol^–1^, respectively.

**Figure 2:**
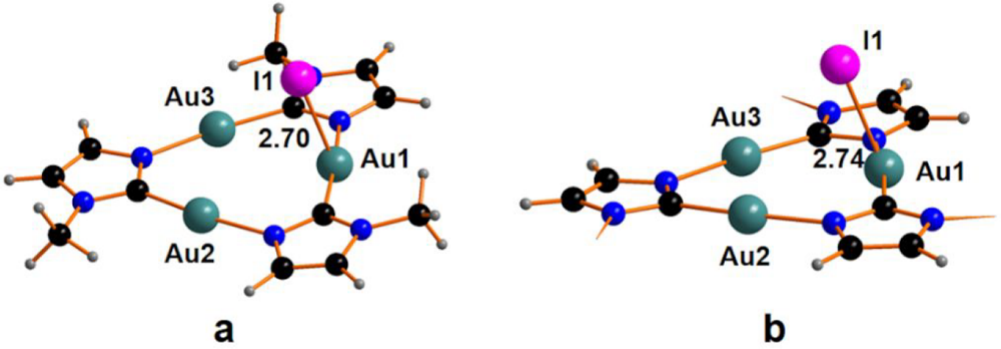
Optimized structure of compound: (a) CTC^Me^I^+^ and (b) CTC^Bz^I^+^. The phenyl rings in panel (b) are hidden for the sake of clarity.

Both structures display an already formed Au–I linkage since the original I1–I2 bonding elongates by *ca.* 0.3 Å, compared to the free I_2_ molecule. The I2–I3–I4 moiety achieves a linear arrangement, with an angle of 175°. The removal of the triiodide anion leaves a cationic intermediate with a gold center in a T-shape coordination. The formation of the cationic species CTC^Me^I^+^ and CTC^Bz^I^+^, shown in Figure 2, has been estimated to be endergonic by +14.5 and +11.6 kcal mol^–1^ for the species with methyl and benzyl substituents, respectively. The computational analysis highlights a stepwise addition of I atoms to the Au center(s) with the formation of tricoordinated intermediates.

The following coordination of the triiodide anion to the Au1 center, in *trans* position to I1, provides the square planar complex featuring two Au–I linkages, compound CTC^Me^I_2_ and CTC^Bz^I_2_, with a free energy gain of −24.1 and −21.8 kcal mol^–1^, respectively. The overall calculated free energy associated to the reactivity between the isolated starting CTC and the first I_2_ molecule, up to the CTCI_2_, featuring a square planar complex, has been estimated to be −21.4 and −25.4 kcal mol^–1^ for the CTC^Me^I_2_ and CTC^Bz^I_2_, respectively. The calculated structural CTC^Bz^I_2_ data is well fitted with the experimental crystal structure reported in the literature.^16^

Further reactivity of the compounds CTCI_2_ with I_2_ molecules evolves similarly, although the cationic intermediates along the pathway feature bridging I between two adjacent Au centers with a slight positive NBO charge of +0.18. Once again, the freed triiodide can perform the attack to a metal center, providing the system with four iodide ligands, two for each metal center. A reasonable explanation of the different reactivity between methyl and benzyl substituents is related to a somewhat hindered attack of the triiodide moiety to the gold in the system with benzyl, while no steric constraints are encountered in the methyl case. The overall process of the activation of three I_2_ molecules with the consequent achievement of three square planar gold centers for the CTC^Me^I_6_, also labeled as compound **1** (overall free-energy gain of −51.2 kcal mol^–1^) is reported in detail in the SI (section 4.1, as well as summarized in Scheme S1 in the Supporting Information).

**Figure 3:**
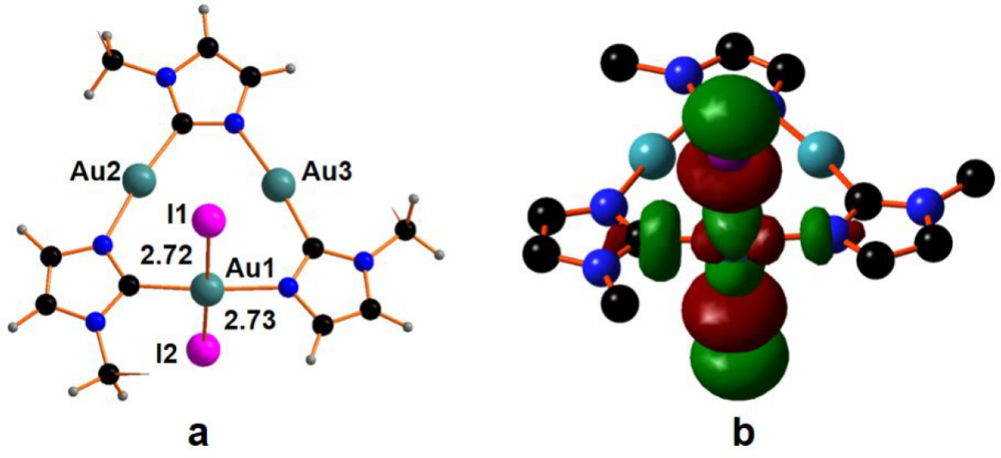
Optimized structure of compound: (a) CTC^Bz^I_2_ and (b) the lowest unoccupied molecular orbital (LUMO) of CTCI_2_ compounds.

As aforementioned, traditionally, the activation of a halogen molecule by a transition-metal complex has been accepted to be accompanied by an increasing oxidation state by two units, thus, precedent publications assigned a formal +III oxidation state to the Au center in compounds related to the square planar CTCI_2_, shown in Figure 3a.

According to the classic ligand field theory (LFT) description,^23,24^ the bonding in a square planar complex is assured by four electronic donations from populated combinations of the ligands into suitable empty metal orbitals. The classical LFT description is based on the assumption that the ligand-centered combinations lie lower in energy than those of the metal. Thus, for energy reasons, in a square planar d^8^ complex, the d_*x*^2^–*y*^2^_ metal orbital should be empty and the lowest unoccupied molecular orbital (LUMO) (antibonding feature), or one of the closer in energy molecular orbitals (MOs) of the system, should be an antibonding σ combination between the empty metal d_*x*^2^–*y*^2^_ and ligand’s orbitals with a stronger contribution from the metal. The corresponding bonding combination is filled at very low energy and mainly centered on the ligands. Some years ago, computational/experimental analysis of the electronic structure of a square planar complex of Cu“(III)”, [Cu(CF_3_)_4_]^−^,^25,26^ highlighted a reverse situation being one combination of the ligands empty and at higher in energy than the d-orbital; this suggests that the d_*x*^2^–*y*^2^_ metal orbital is populated and the metal electronic configuration could be better described as d^10^ more than a d^8^. The idea of Inverted Ligand Field (ILF) purposes an alternative bonding pattern in a square planar complex with three ligands to metal donations and the fourth interaction interpreted as a metal-to-ligands σ–donation. The presence of an empty ligand-centered combination influences the overall electronic structure with the LUMO now mainly centered on the ligands rather than on the metal. This description perfectly agrees with the bonding pattern in CTCI_2_ without any substantial variation due to the nature of the substituent at the imidazole rings. In this perspective, the LUMO, shown in Figure 3b, has a small contribution from the metal (28.5%) and 71.5% from the ligands, and 53.5% of this latter component comes from the iodide moieties. Thus, a formal +1 oxidation state could be reasonably assigned to the Au1 center, as well as in the Au2 and Au3 and the ILF description may be applied to all the products at any degree of iodinization, as well as to all tricoordinated intermediates encountered along the reaction pathway.

The ILF occurrence is also confirmed by the detailed NBO population analysis on compound CTCI_2_, revealing no significant difference in d-population of Au1, compared to the Au2 or Au3 center, with 9.42 d electrons associated with Au1, only 0.18 e^–^ less than those at Au2 or Au3, once again confirming the ILF occurrence.

Another confirmation of the ILF could be found through structural comparison between the experimental/optimized structures of the starting compound and the product after the substrate activation. In this regard, the X-ray structures did not point out a shortening of the metal–ligand distances, as expected by an oxidative process. The occurrence of the ILF might be imputed to the specific reaction with I_2_ molecules given the very close electronegativity of the gold and iodine (2.54 *vs.* 2.66). In this regard, a computational analysis on the simple square planar [AuCl_4_]^−^ anion revealed a similar behavior with the LUMO being an antibonding orbital mainly localized on the four Cl atoms (71%) and only 29% from the Au atoms. A reverse situation occurs for the σ-bonding counterpart (HOMO–15) well stabilized in energy and with a strong contribution (*ca.* 68%) from the metal. This allows concluding that the ILF is ubiquitous in the chemistry of square planar gold complexes also when associated with more electronegative elements, such as chlorine.

### The Reactions of CTC^Me^ and CTC^Bz^ with MeI

Afterward, the reactions of CTC^Me^ and CTC^Bz^ with MeI were performed under mild experimental conditions, using MeI as the solvent and as the reactant. Methyl iodide readily reacts with several types of Au(I) compounds^17−22^ and recently it has been proven that MeI can oxidize gold metal to form the [CH_3_AuI] derivative.^27^ In practice, an excess of MeI was added to the solid Au(I) cyclotrimers upon magnetic stirring at room temperature under an inert atmosphere. After the dissolution of the CTC^Me^ or CTC^Bz^ in MeI, the reaction mixtures were monitored by spectroscopies during the time. In the case of CTC^Me^, the starting cyclotrimer was largely persistent in the reaction mixture, even after 1 day of stirring; in fact, by monitoring the reaction mixture between CTC^Me^ and MeI by ^1^H NMR spectroscopy, recording the appearance of the signal at 1.78 ppm attributed to a methyl bound to the Au(I),^19^ compound **3** was present at 5%–8%, with respect to the starting CTC^Me^ after 3–5 h of mixing. However, the isolation of compound **3** as yellow crystals was obtained with a yield of 36% after 1 week at 5 °C by adding hexane to the reaction mixture. The crystals of compound **3** are not emissive upon irradiation at 366 nm, they exhibit rather good bench stability, which becomes low once in CDCl_3_ solution, with consequent return of the starting cyclotrimer and MeI in large extent (see Figure S8 in the Supporting Information). The ^1^H NMR and ^13^C NMR spectra of the mixture generated upon dissolution of compound **3** in CDCl_3_ are reported in Figures S8 and S9 in the Supporting Information. However, in the ^13^C NMR spectrum, three peaks were found in the region 160–170 ppm: the most intense due to CTC^Me^ (168 ppm) and two small signals at 165 and 167 ppm, which might be tentatively assigned to compound **3** (Figure S9). The solid-state characterization by elemental analysis and IR spectra (see Figure S10 in the Supporting Information) support the evidence that compound **3** is made of the CTC^Me^ and MeI, but the real nature of compound **3** was confirmed only after the results of the XRD analysis on the single crystals (see below).

**Scheme 2:**
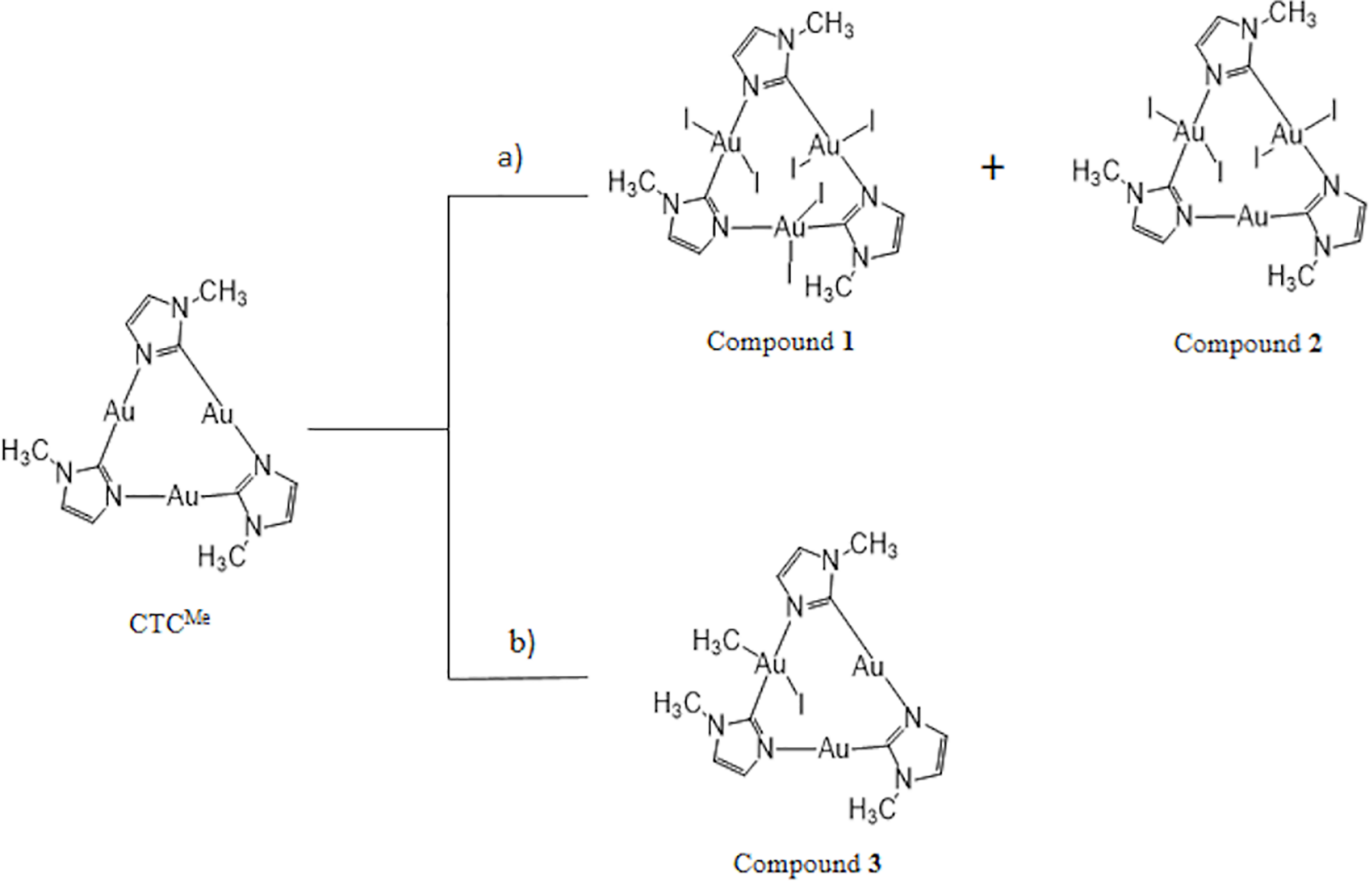
Schematic View of the Reactions of CTC^Me^ with (a) an Excess of Iodine, (b) an Excess of MeI, and the Corresponding Products

The reactivity between CTC^Me^ with different substrates is depicted in Scheme 2.

**Scheme 3:**
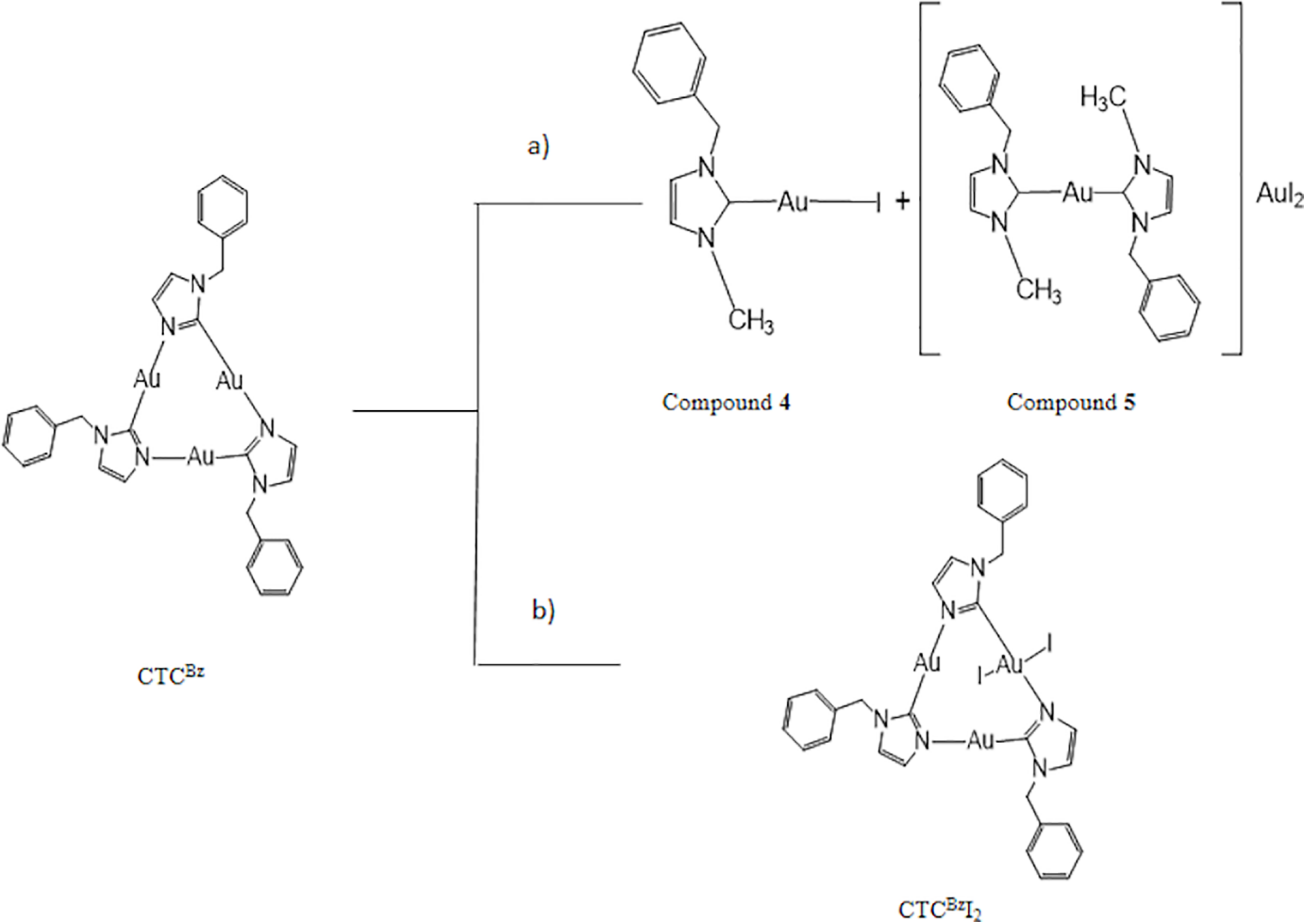
Schematic View of the Reactions of CTC^Bz^ with (a) 3 mol of Methyl Iodide, R = Me and X = I (Compound **4**), R = Me and X = AuI_2_ (Compound **5**) and (b) 3 mol of Iodine Data taken from ref (16).

In the case of the reaction of CTC^Bz^ and MeI, the monitoring of the reaction revealed the ready appearance of two new compounds, in addition to the starting cyclotrimer, one presumably being ionic. The crystallization of the reaction mixture of MeI and CTC^Bz^ provided a microcrystalline solid, intensively glowing in the yellow upon irradiation at 366 nm, consisting predominantly of two phases. The ^1^H NMR spectrum shows two sets of signals in a ratio that are dependent on the solvent used for the NMR (see Figure S11 in the Supporting Information). In contrast to what was observed for compound **3**, the microcrystalline solid was not completely soluble in CDCl_3_. The ^1^H NMR spectrum recorded in DMSO-d^6^ exhibits only two signals for the N–CH_3_, at 3.78 and 3.81 ppm in a 2:1 integral ratio. The striking feature useful for the attribution of these peaks was rendered by the ^13^C NMR spectrum (see Figure S12 in the Supporting Information); in fact, it displays two sets of signals in a 2:1 ratio at 181 and 184 ppm, attributable to the C atoms in position 2 of the imidazole in a typical range of chemical shifts for carbene species.^28,29^ The elemental analysis of the microcrystalline solid was interpreted for a mixture of a monocarbene-gold(I) compound (**4**) and a bis-carbene-gold(I) compound (**5**) in a 1:1 ratio (see Scheme 3, path a). The FIR spectrum, recorded in the microcrystalline solid, displays an intense band at 199.95 cm^–1^, which was attributed to the antisymmetric vibrational mode (ν_3_) of the AuI_2_^–^ anion in the microcrystalline solid, confirming the presence of this counterion (see Figure S13 in the Supporting Information).^30^

The formation of carbene species from the reaction of CTC^Bz^ with acyl or alkyl halides^16,31^ and with HCl is already known. Regarding the reaction with HCl, it was reported the formation of the cationic bis(l-benzylimidazolyl-2-yl)gold(I) chloride, by the treatment of the likely 1-benzyl-imidazolate-2yl-gold-triphenylphosphane derivative with an aqueous solution of HCl.^31^ Also, the straight reaction of CTC^Me^ or CTC^Bz^ with an aqueous solution of HCl results in the formation of carbene species (section 1.1 in the Supporting Information).

In the attempt to understand the different reactivity attained with the two starting cyclotrimers, the ^13^C NMR data were considered. Table 1 reports the ^13^C NMR chemical shifts for the C2 of the compounds **1**–**5**, compared with the respective starting cyclotrimers and carbene species (see section 1.1 in the Supporting Information). Compound **6**, [(Im^Me^-2yl)_2_Au]Cl (where Im^Me^ is the 1-methyl-2yl-imidazole), displays a ^13^C NMR signal for the C2 atom at 167 ppm in CDCl_3_, which is in the range reported for dialkyl-NHC mono- or bis-carbene gold(I) derivatives,^28^ and for the C2 of the corresponding CTC^Me^ (167 ppm), while the C2 of the free 1-methyl imidazole falls at 137 ppm in CDCl_3_; the C2 of compound [(Im^Bz^-2yl)_2_Au]Cl falls at 180 ppm in DMSO-*d*^6^,^8^ which is in the same range of those reported for compounds **4** and **5** and at rather higher frequencies, if compared to that of the starting CTC^Bz^ (167 ppm, while the free 1-benzyl imidazole falls at 137 ppm in CDCl_3_). Finally, although shifts of 30 ppm are observed for the ^13^C NMR signals of the C2 atoms upon cyclization and formation of the corresponding CTCs, only the C2 of the 1-benzylimidazole carbene species is additionally shifted to higher frequencies (180 ppm) after the formation of the carbene compound (see Table 1). Generally, the ^13^C NMR signal for the C2 atom in NHC-carbene compounds moves toward high frequencies to an extent that is dependent on the Lewis acidity of the metal center and on the ability of the positively polarized C2 to withdraw π-electron density from the imidazole ring.^32^ Hence, by considering the herein compounds the ^13^C chemical shift in compound **7**, [(Im^Bz^-2yl)_2_Au]Cl (where Im^Bz^ = 1-benzyl-2yl-imidazole), indicates that the C2 is slightly richer in electron density than those of compounds **4** and **5**, classified as carbenes.^8^ Moreover, the electron density of C2 in compound **6** seems to be comparable to that of the corresponding C2 in CTC^Me^; in fact, the ^13^C signals fall at 167 ppm for both compounds: the carbene **6** and the CTC^Me^. From these data, the 1-methylimidazole seems to supply more electron density on the C2 and, thus, on the gold center, conversely, the 1-benzyl-imidazole provides larger stability to the corresponding carbene gold(I) complexes, likely as an effect of the larger aromatic delocalization, despite a lower electron density on the metal center.

**Table 1:** ^13^C NMR Chemical Shifts for the C2-Imidazole for Compounds **1**–**7** and Relative Reference Compounds^a^

compound	^13^C chemical shift (ppm, DMSO-*d*^6^)
Im^Me^	137^b^
Im^Bz^	137^b^
CTC^Me^	167,^b^ 168^c^
CTC^Bz^	167^b^
compound **1**, CTC^Me^I_6_	166
compound **2**, CTC^Me^I_4_	166
compound **3**, CTC^Me^-MeI	165, 167^c^
compound **4**, Im^Bz^-2yl-AuI	184
compound **5**, [(Im^Bz^-2yl)_2_Au]AuI_2_	181
compound **6**, [(Im^Me^-2yl)_2_Au]Cl	167^d^
compound **7**, [(Im^Bz^-2yl)_2_Au]Cl	180^b^

a Legend of compounds: Im^Me^ = 1 methyl-imidazole; Im^Bz^ = 1-benzyl-imidazole; CTC^Me^ = [μ–Au-C^2^,N^3^-1-methyl-imidazolate]_3_; and CTC^Bz^ = [μ–Au-C^2^,N^3^-1-benzyl-imidazolate]_3_.

b Data taken from ref (8).

c Recorded in CDCl_3_ solution,

d See electronic Supporting Information.

Moreover, Table 1 highlights only one signal for the C2 for the imidazoles in **2**, even though in compound **2**, two different chemical environments are expected: one for the carbon attached to the square planar Au, N–AuI_2_-C, and another for the linear N–Au–C; actually, this evidence might be explained right as a consequence of the ILF, as the electronic populations in both Au centers are similar, as discussed above.

### Computational Studies on the Reaction of Methyl Iodide with CTC^Me^ and CTC^Bz^

The reactivity between the initial CTC and CH_3_I has highlighted the great influence of the substituents at the imidazole ring, since, with the CTC^Bz^, the main products are the monocarbene and bis-carbene species. Otherwise, the reaction with CTC^Me^ partially evolves to compound **3** featuring a Au center in a square planar arrangement bonded to the methyl and the iodide. The dissolution of the crystals of **3**, with the reformation of the starting CTC^Me^, underlines the instability of the square planar system in solution. In view to provide a reasonable overview of the electronic factors ruling the reactivity with the methyl iodide, a computational analysis has been conducted, similarly to the precedent I_2_ case.

#### Methyl Iodide Activation

**Figure 4:**
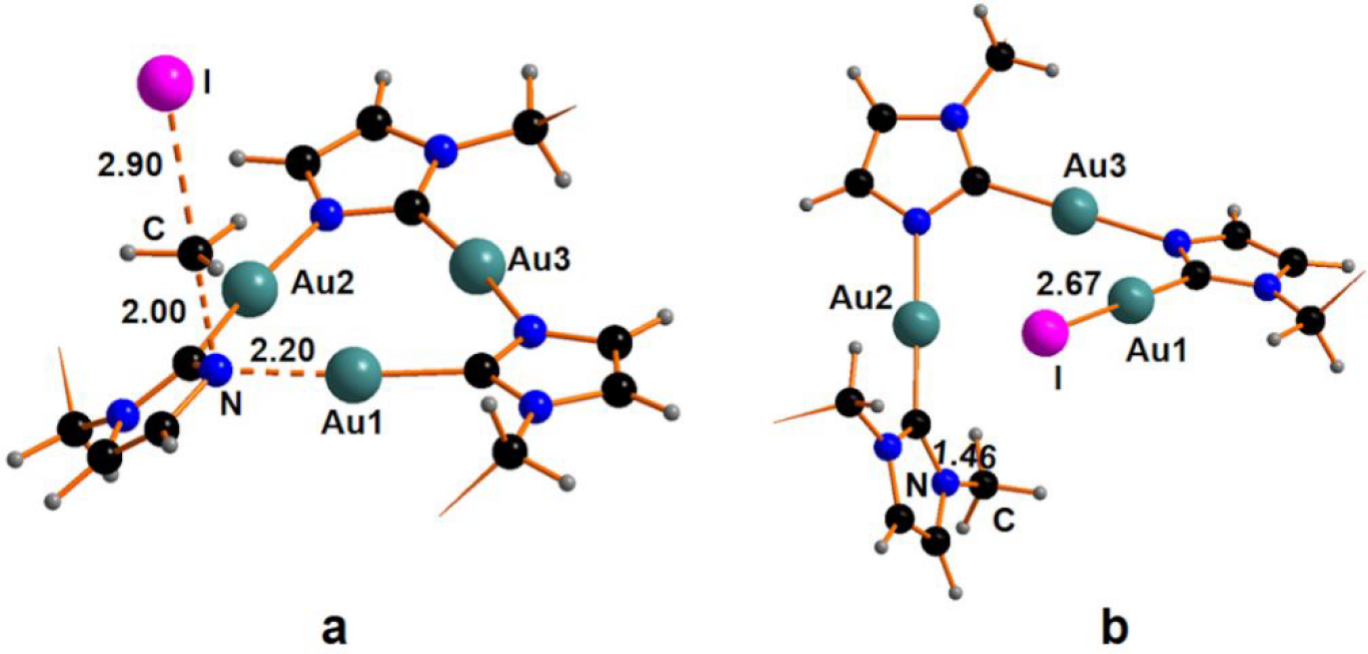
Optimized structures of (a) transition state **8**^**Bz**^_**TS**_ and (b) intermediate **8**^**Bz**^.

All the computational efforts to activate the CH_3_I molecule by the Au center, maintaining the cyclic structure of the CTC, failed, since all the relaxed scans, obtained via stepwise shortening of the Au–C or Au–I distances, provided no reasonable results or too high an energy barrier being higher than 40 kcal mol^–1^. Alternatively, the computational analysis revealed the non-innocent behavior of the imidazole in the activation of the methyl iodide, because of the presence near to the frontier of populated delocalized π-system of the imidazole able to interact with the incoming methyl iodide for the potential formation of a new N–C linkage. This is confirmed by the detection of a transition state, **8**^**Bz**^_**TS**_, shown in Figure 4a, featuring the initial formation of the N3–C bonding and the cleavage of the H_3_C**···**I one, being as large as 2.90 Å, 0.7 Å longer than in the pristine free substrate. This allows also the initial cleavage of the Au1–N bonding with a *ca.* 0.2 Å elongation. The associated free-energy barrier for the achievement of **8**^**Bz**^_**TS**_ is +21.2 kcal mol^–1^, featuring a planarization of the transferred methyl group (I–C–H angle being 95.7°), in some way, the aromatic electrophilic substitution. The elongation of the Au–N linkage by *ca.* 0.2 Å associates with a redistribution of the electronic density and the formation of a carbene ligand bonding the Au2 center.

**Figure 5:**
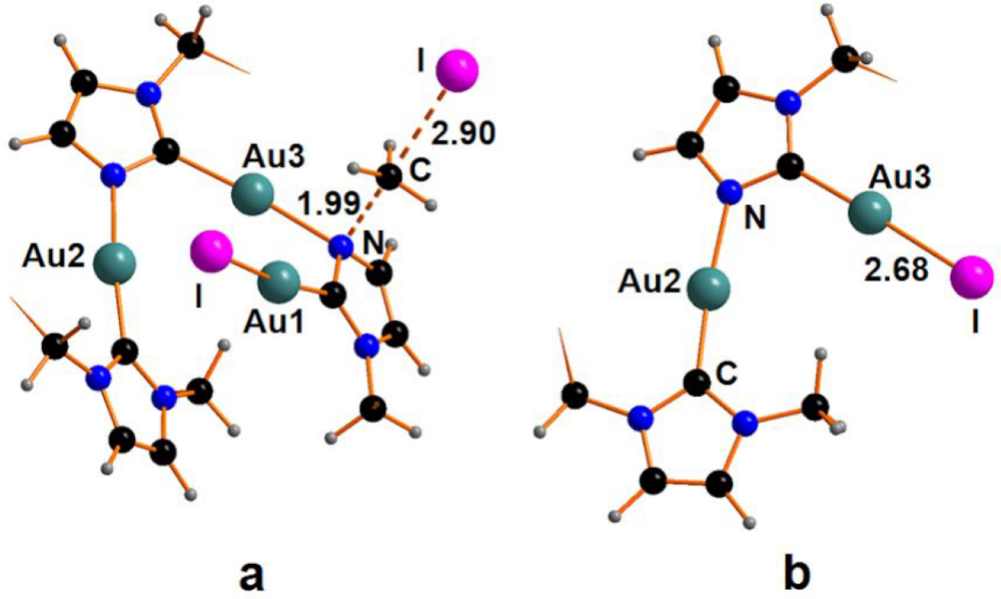
Optimized structures of (a) transition state **9**^**Bz**^_**TS**_ and (b) dinuclear intermediate **9**^**Bz**^.

After the transition state **8**^**Bz**^_**TS**_, compound **8**^**Bz**^ is obtained, featuring the new N–C bond and the N–Au1 one with the unsaturation at the Au1 immediately compensated by the iodide coordination. The overall free-energy gain, associated with the formation of **8**^**Bz**^ from **8**^**Bz**^_**TS**_, has been estimated to be as large as −44.5 kcal mol^–1^, double than the energy barrier required for the achievement **8**^**Bz**^_**TS**_. Once a side of the cycle is broken, the system may evolve toward the formation of three monocarbene Au(I) compounds **4**, by stepwise cleaving the still-present Au–N linkages. In particular, the interaction between **8**^**Bz**^ with a second CH_3_I molecule evolves through the formation of a transition state, namely, **9**^**Bz**^_TS_ (see Figure 5a) with a free-energy barrier of +23.7 kcal mol^–1^ after that the formation of a carbene/iodide linear gold(I) complex **4** occurs together with a dinuclear species **9**^**Bz**^, shown in Figure 5b with a free-energy gain of −31.9 kcal mol^–1^.

**Figure 6:**
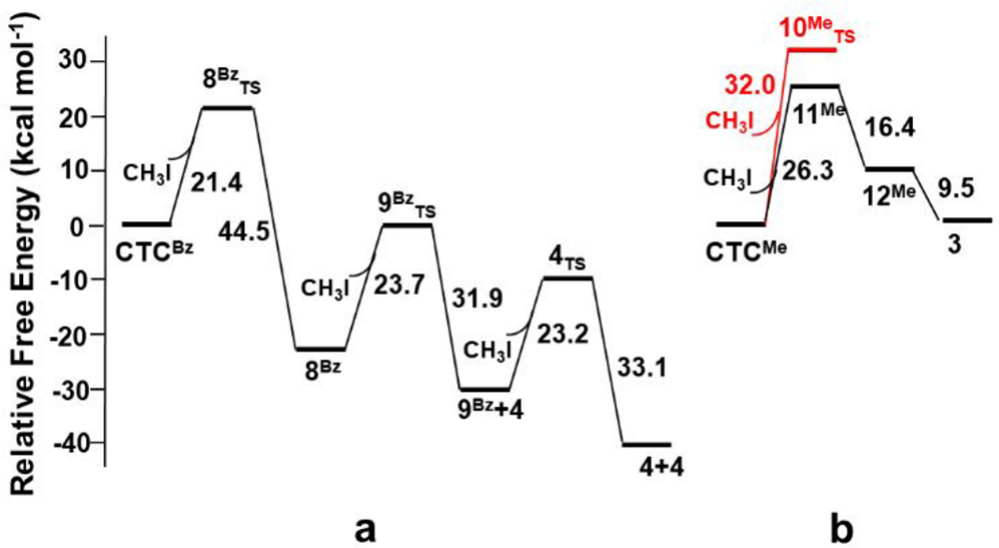
Energy diagrams showing the energy profiles for the proposed mechanisms for the electrophilic addition of MeI additions to (a) CTC^Bz^ and (b) CTC^Me^. The red pathway in panel (b) has been dismissed, given the too-high energy barrier.

The dinuclear unit **9**^**Bz**^ reacts with a third methyl iodide molecule and, after bypassing a free-energy barrier of 23.2 kcal mol^–1^ associated with the transition state **4**_**TS**_, two new units of **4** are exergonically formed (−33.1 kcal mol^–1^). An overall free energy of −41.4 kcal mol^–1^ has been estimated for the formation of three isolated molecules of **4**. The overall reaction pathway for the reaction between CTC^Bz^ and CH_3_I up to three molecules of **4** is summarized in Figure 6a.

A reasonable explanation for the experimental obtainment of a neutral compound **4** and the ionic pair compound [Au(1-benzyl-3-methyl-2-imidazolyl-2yl)_2_]AuI_2_, namely **5**, is also provided. In this regard, similarly to the recent work of Gust, Podewitz et al.,^33^ a scrambling ligand mechanism may occur involving the interaction between two molecules of **4**. In particular, compound **5** is originated with a total free energy gain of −13.8 kcal mol^–1^, after bypassing a free-energy barrier of 20.6 kcal mol^–1^. The pairing of two linear complexes of **4** through weak aurophilic interactions has been estimated to be exergonic by −12.5 kcal mol^–1^ with a calculated Au**···**Au distance of 3.70 Å.

Concerning the reaction between the CTC^Me^ and CH_3_I, the experiments have been revealed the formation of a very small amount of compound **3** featuring square planar coordination around one Au center due to the formation of two new bonds with methyl and an iodide moiety. The computational analysis of the reactivity between CTC^Me^ and CH_3_I highlights a striking difference with the benzyl analogue CTC^Bz^, given the too-high free-energy barrier for the achievement of the transition state, **10**^**Me**^_**TS**_ (32 kcal mol^–1^), red pathway in Figure 6b. Other reaction processes have been also explored by maintaining the cyclic planar structure of the CTC, but all pathways have been discarded given the high energy barriers. In particular, a relaxed scan for the extraction of the methyl group directly by the involved metal center through an S_N_2-type reaction has highlighted an energy barrier of >30 kcal mol^–1^, consistent with some already reported pathways requiring some drastic conditions, such as high temperature.^34^ The only remaining reasonable process should involve the cleavage or at least the weakening of the Au–N linkage, followed by the interaction between the metal center and the incoming methyl iodide through the iodine. Also, this process is not straightforward being the formation of compound **11**^**Me**^, shown in Figure S14 in the Supporting Information, endergonic by 26.3 kcal mol^–1^ compared to the isolated **CTC**^**Me**^ and CH_3_I. The high free-energy cost is due to the cleavage of the Au–N bonding only slightly compensated by the interaction between the gold and iodine. Possibly, intermediate **11**^**Me**^ may evolve to the final compound **3** after the achievement of the T-shape compound **12**^**Me**^, shown in Figure S15 in the Supporting Information, after the splitting of the C–I bonding. Compound **12**^**Me**^ is formed with a free-energy gain of −16.4 kcal mol^–1^ and then the system evolves toward the final compound **3** featuring square planar coordination around one Au center. The last process must involve an isomerization from the cis configuration to the trans one in **3**. The overall reaction from **CTC**^**Me**^ and the CH_3_I up to compound **3** has been estimated to be endergonic by +0.4 kcal mol^–1^ and the presence of high barriers along the pathway is consistent with the very low yield of the reaction of *ca.* 8% after 5 h in solution up to a maximum of 36% after a week as a solid.

**Figure 7:**
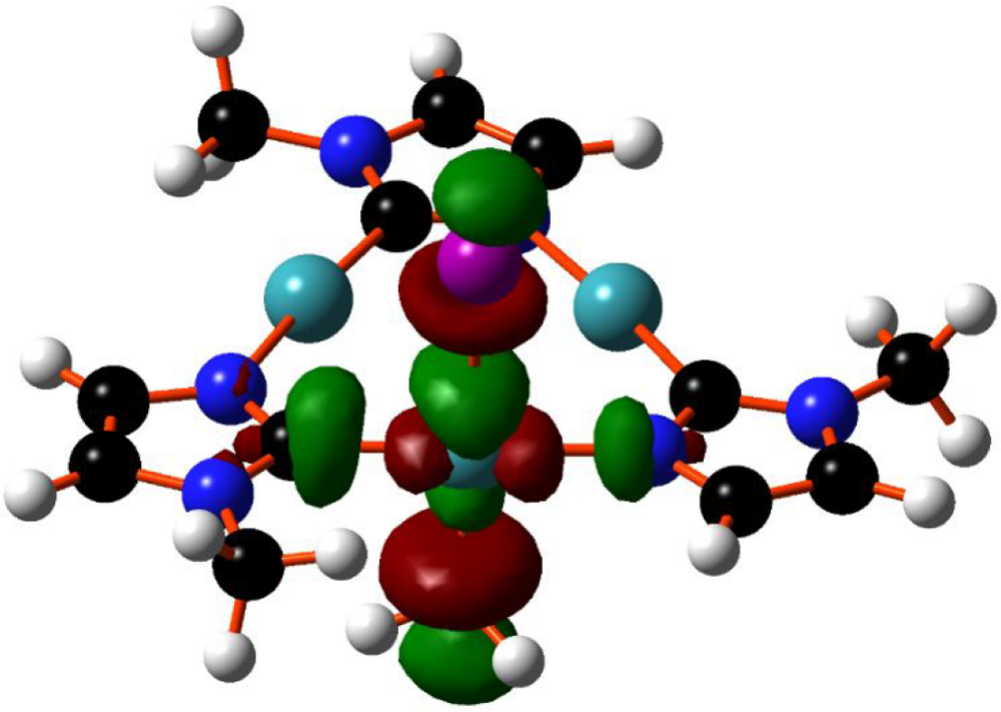
Plot of the LUMO orbital of compound **3**.

Similar to the case of **CTCI**_**2**_, also in compound **3**, the bonding pattern could be reasonably explained in the light of ILF theory. The LUMO, shown in Figure 7, is mainly localized on the ligands rather than on the metal (Au contribution is 34% vs. 66% from the ligands). Thus, also, in this case, it is reasonable to assign a d^10^ configuration to the metal rather than the expected d^8^.

As already described above, compound **3** displays experimental features needing an interpretation such as the dissociation of crystals of **3** to the MeI and the parent CTC in CHCl_3_ solution. The computational analysis revealed that the dissociation of the crystal to reform CTC could not be explained with a mechanism involving the ring opening. In this regard, ad-hoc relaxed scans, performed via stepwise weakening of the Au–N bonding, revealed too-high energy barriers (>25 kcal mol^–1^). Otherwise, the iodide ligand seems to be more easily displaced (energy cost of *ca.* 15 kcal mol^–1^), and, once freed, may move toward the methyl ligand for a nucleophilic attack, restoring the methyl iodide. Such a process is confirmed by a slight positive charge on the methyl ligand, with the calculated NBO charge being +0.17.^35^

### X-ray Crystal Structure Description of Compounds **2** and **3**

**Figure 8:**
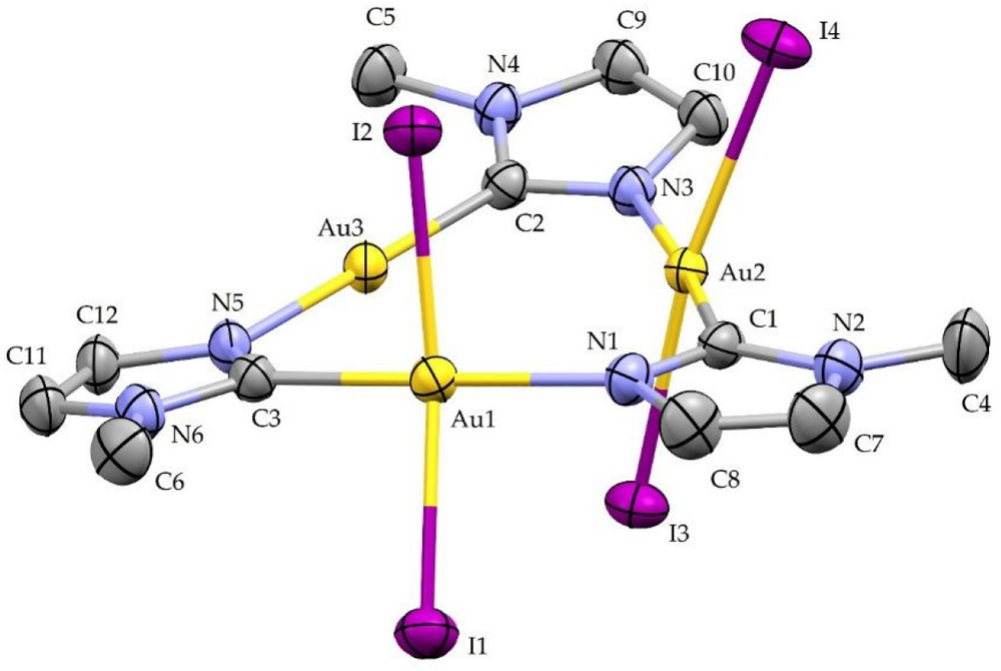
ORTEP style view of compound **2**. Ellipsoids are drawn at their 50% probability level. Hydrogen atoms and THF solvent molecule are omitted for the sake of clarity. Selected bond distances: C1–N1, 1.338(5) Å; C1–Au2, 2.006(3) Å; C2–N3, 1.350(4) Å; C2–Au3, 1.989(3) Å; C3–N5, 1.332(4) Å; C3–Au1, 2.005(3) Å; Au1–N1, 2.070(3) Å; Au1–I2, 2.6121(3) Å; Au1–I1, 2.6244(3) Å; Au2–N3, 2.051(3) Å; Au2–I3, 2.6119(3) Å; Au2–I4, 2.6235(3) Å; Au3–N5, 2.055(3) Å. Selected bond angles: N1–C1–Au2, 124.4(2)°; N3–C2–Au3, 123.1(2)°; N5–C3–Au1, 120.9(2)°; C3–Au1–N1, 172.12(13)°; I2–Au1–I1, 165.056(10)°; C1–Au2–N3, 174.39(13)°; I3–Au2–I4, 170.404(10)°; C2–Au3–N5, 174.87(12)°; C1–N1–Au1, 122.6(2)°; C2–N3–Au2, 120.0(2)°; C3–N5–Au3, 124.2(2)°.

**Figure 9:**
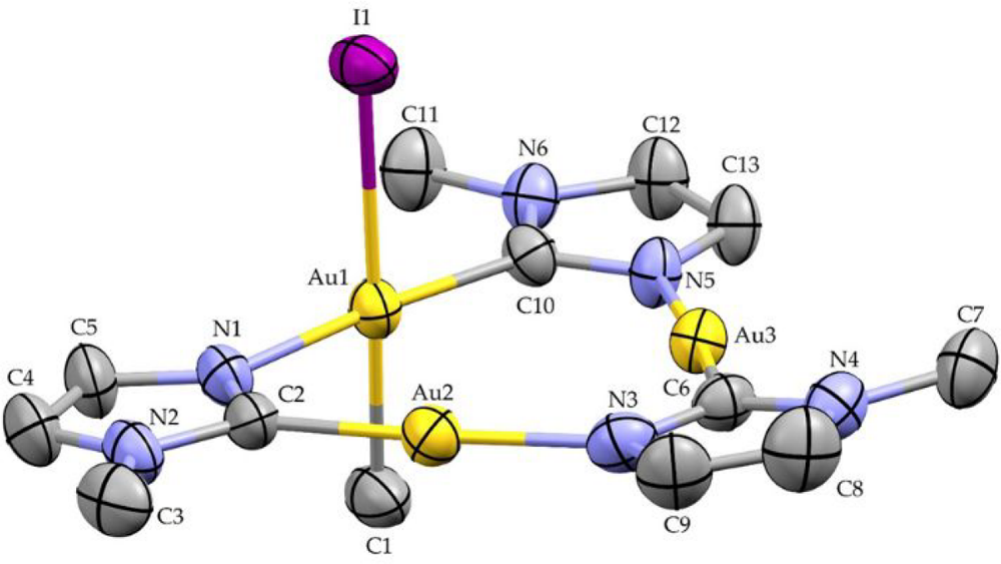
ORTEP style view of compound **3**. Ellipsoids are drawn at their 50% probability level. Hydrogen atoms are omitted for the sake of clarity. Selected bond distances: C1–Au1, 2.151(8) Å; C2–N1, 1.349(10) Å; C2–Au2, 1.977(8) Å; C6–N3, 1.352(10) Å; C6–Au3, 1.984(7) Å; C10–N5, 1.340(9) Å; C10–Au1, 1.994(6) Å; Au1–N1, 2.046(6) Å; Au1–I1, 2.6957(6) Å; Au2–N3, 2.034(7) Å; Au3–N5, 2.036(6) Å. Selected bond angles (deg): C10–Au1–N1, 175.3(3)°; C1–Au1–I1, 175.7(3)°; C2–Au2–N3, 175.3(3)°; C6–Au3–N5, 174.7(3)°; C2–N1–Au1, 120.0(5)°; C6–N3–Au2, 122.2(5)°; C10–N5–Au3, 121.1(5)°; N1–C2–Au2, 123.6(5)°; N3–C6–Au3, 122.7(5)°; and N5–C10–Au1, 123.2(5)°.

The ORTEP style view of compounds **2** and **3** are reported in Figures 8 and 9, respectively, together with their atomic labeling schemes. The most significant bond distances and angles are reported in the caption of the figures, while the crystal data are reported in Table 2.

**Table 2:** Crystal Data for Compounds **2** and **3**

	compound **2** · THF	compound **3**
empirical formula	C_12_H_15_Au_3_I_4_N_6_**·**C_4_H_8_O	C_13_H_18_Au_3_IN_6_
formula weight	1413.90	976.14
temperature	200 K	250 K
wavelength	0.71073 Å	0.71073 Å
crystal system	monoclinic	monoclinic
space group	*P*2_1_/*n*	*P*2_1_/*c*
Hall group	–*P*2*yn*	–*P*2*ybc*
unit-cell dimensions	*a* = 12.3839(4) Å	*a* = 13.7696(9) Å
	*b* = 14.7473(4) Å	*b* = 9.6146(6) Å
	*c* = 15.7657(5) Å	*c* = 14.6662(10) Å
	β = 107.066(1)°	β = 95.941(2)°
volume	2752.49(15) Å^3^	1931.2(2) Å^3^
*Z*	4	4
Density (calculated)	3.412 g/cm^3^	3.357 g/cm^3^
absorption coefficient, μ	20.457 mm^–1^	24.339 mm^–1^
F(000), F(000′)	2472.0, 2443.69	1712.0, 1689.36
*T*_min_, *T*_max_	0.4586, 0.7460	0.546, 0.746
Theta(max), data completeness	30.087, 0.998	29.257, 0.999
*R* (reflections)	0.0184 (7785)	0.0314(4666)
*wR*2 (reflections)	0.0434 (8079)	0.0877(5250)
S	1.008	1.006
Npar	289	208

The molecular structure of compound **2** displays a skeletal core analogous to that of compound **3**. Compound **2** consists of a trinuclear gold cyclic molecule, in which three imidazolate moieties bridge three metal centers through C and N atoms, forming an almost planar nine-membered ring. In compound **2**, the nine-membered ring is deviated from planarity, especially atoms C2 and N3, which are at a distance of 0.262(2) and 0.187(2) Å, respectively, from the plane defined by the three gold atoms Au1, Au2, and Au3. Similar to compound **3**, the imidazole rings also are sloped, with respect to the gold plane, with dihedral angles ranging from 7° to 15°. The Au(III) atoms are in a distorted square planar coordination environment. The I–Au–I bond angles significantly deviate from linearity with values of 165.06(1)° and 170.40(1)°. The Au···Au separations are 3.470(1), 3.532(1), and 3.594(1) Å, with the longer distance belonging to the oxidized Au(III) atoms and no aurophilic interactions, are observed. Noteworthy, regardless of what was observed for analogues, the crystal packing of compound **2** exhibits rather short intermolecular I···I interactions at 3.593 and 3.887 Å, resulting in the pairing of two by two molecules to form an extended chain of I···I–Au–I···I–Au–I···I· interactions with Au–I distances of 2.624 and 2.612 Å for the two Au–I bonds (see Figure S17 in the Supporting Information). This evidence may explain the lower solubility recorded for compound **2**, with respect to the similar compounds reported in Table 3 and prepared by Balch.

**Table 3:** Selected Au–N and Au–C Bond Distances for Pristine CTCs Obtained with Different Ligands^a^ and Au–N and Au–C Bond Distances of the Relative Products Obtained by the Reaction of the CTCs with Iodine

	CTC^Bz^I_2_^b^	CTC^carb^I_2_^c^	CTC^carb^I_4_^c^	CTC^carb^I_6_^c^	CTC^Me^I_4_
Au(III)–N	1.913 (Au1–I)	2.052 (Au1–I)	2.072 (Au1–I)	2.082 (Au1–I)	2.051 (Au1–I)
Au(I)–N	2.013 (Au2)	2.072 (Au2)	2.095 (Au2–I)	2.086 (Au2–I)	2.070 (Au2–I)
Au(I)–N	2.063 (Au3)	2.052 (Au3)	2.012 (Au3)	2.074 (Au3–I)	2.054 (Au3)
Au(III)–C	1.964 (Au1–I)	2.032 (Au1–I)	2.022 (Au1–I)	2.032 (Au1–I)	2.006 (Au1–I)
Au(I)–C	2.024 (Au2)	2.022 (Au2)	2.012 (Au2–I)	2.000 (Au2–I)	2.004 (Au2–I)
Au(I)–C	2.005 (Au3)	1.972 (Au3)	2.065 (Au3)	2.065 (Au3–I)	1.989 (Au3)
	**CTC**^**Bz**^^d^	**CTC**^**carb**^			**CTC**^**Me**^
Au(I)–N	2.042	2.032			nd^e^
	2.051				
	2.036				
Au(I)–C	1.978	2.000			nd^e^
	1.998				
	2.003				

a CTC^Me^ = [μ–Au-C^2^,N^3^-1-methyl-imidazolate]_3_, CTC^Bz^ = [μ–Au-C^2^,N^3^-1-benzyl-imidazolate]_3_, CTC^carb^ = [μ–Au-C^2^,N^3^- *N*-methyl-C-methoxy-carbonate]_3_.

b Data taken from ref (16).

c Data taken from ref (14).

d Data taken from ref (36)..

e nd = not determined.

The core of the molecule in compound **3** resembles that of compounds already described.^16,36,37^ The two Au(I) atoms, namely Au2 and Au3, are at a distance of 3.507(2) Å, which is longer than those observed in the parent 9-membered ring reported in the literature [3.349 Å mean value found in the CCDC 2020 database]. Au2 and Au3 both show a nearly linear coordination environment, with C–Au–N bond angles of 175.3(3)° and 174.7(3)°, respectively, while the Au1 atom, displaying the bonding with the methyl and the iodine ligands, features square planar coordination. The Au–I and Au–C1 bond distances are 2.6957(6) and 2.151(8) Å, respectively. The Au–I bond distance is slightly longer than those observed in the derivatives obtained by the addition of iodine to CTCs.^16,2^ In compound **3**, the intramolecular Au···Au separations are 3.471(1), 3.489(2), and 3.507 Å, respectively, with the longer distance belonging to the centers not involved in the bonding with the methyl and the iodine, the Au2···Au3, indicating a scalene triangular metal frame and no aurophilic interactions. The maximum deviation from the mean plane of the coordination around Au1 is observed for C1 atom [0.089(1) Å]. The 9-membered ring is almost planar. The dihedral angle between this mean plane and the coordination plane around Au1 is 85.36(1)°. The three imidazolate rings are slightly sloped, with respect to the mean plane of the 9-membered ring. The widest dihedral angle is observed for the imidazolate unit bridging the two Au(I) atoms: the dihedral angle between the C6,N3,C9,C8,N4 unit and the mean plane of the 9-membered ring is 7.04(1)°. Remarkably, the crystal packing exhibits neither aurophilic nor halogen interactions and it is built up through normal van der Waals interactions (see Figure S18 in the Supporting Information). The Au···Au separation between adjacent molecules is a minimum of 4.980(1) Å.

Notably, in compound **3**, the Au1–C10 and Au1–N1 bond distances (1.994(6) Å and 2.046(6) Å, respectively) are slightly longer than those observed for the other two metal centers not connected in the Me–Au-I unit: the Au2–C2 and Au2–N3 bond distances are 1.977(8) and 2.034(7) Å, respectively, while the Au3–C6 and Au3–N5 distances are 1.984(7) Å and 2.036(6)Å, respectively. This trend of bond distances is also adopted in the products obtained by the stepwise oxidation by iodine of the *N*-methyl-C-methoxy-carbeniate gold(I) CTC,^14^ and it was found also in the case of the product obtained from the reaction of iodine with the 1-benzylimidazolate gold(I) CTC.^16^ Some representative Au–C and Au–N bond lengths of these compounds fully or partially oxidized with iodine CTCs are shown in Table 3. For compound **3**, the crystal data of the starting cyclotrimer are not available, but by analyzing the bond lengths reported in Table 3, it indicates that the addition of iodine to the Au centers does not afford a shortening of the Au–C and Au–N bond distances, since it would be expected as a consequence of the oxidation of Au centers from a formal +1 oxidation state to a +3 oxidation state and the relative size contraction.

## Conclusions

The reactivity of two gold CTCs, consisting of Au(I) centers and imidazole as bridging ligands having methyl or benzyl substituents at the N1 of the imidazole, has been considered toward probing reactants such as iodine, methyl iodide, and hydrochloric acid. Despite the close similarity of these starting CTCs systems, different experimental outcomes have been obtained. The results can be summarized as (i) the breakage of the cycles and the formation of monoligated or bis-ligated NHC Au(I) structures and (ii) the obtaining of CTC units with square planar tetra-coordinated Au centers. Remarkably, relative to the reaction of CTCs with MeI, different outcomes were attained: although in the case of the methyl-imidazole CTC, the square planar Au center slowly forms, for the benzyl imidazole CTC, the mono- and bis-NHC-gold(I) carbene structures are the only compounds formed and isolated. Following the same experimental conditions, only the presence of the two different substituents differentiates the outcomes. Theoretical studies provided essential help to unravel the enigma fixing the substituents at the N1 of the imidazole, as the activator or less of the other nitrogen, the N3, affording to two alternative mechanisms of addition: by the metal activation or by the π-aromatic imidazole interaction.

Although fully worth it, these are not the only remarkable results obtained from this work. Beyond the comparison of the ^13^C NMR imidazole C2 signal chemical shifts, whose interpretation is not straightforward, the analysis of the experimental Au–N and Au–C bond distances in the crystal structure of compounds **2** and **3** highlights very slight bond length contractions for the square planar Au centers, if compared to those of the bicoordinated Au centers. Computational calculations on these products attained electronic populations that are closer to d^10^ than to d^8^ configurations, opening a controversial issue on the oxidation of the Au centers.

An appropriate description of the bonding in this family of compounds could be obtained by evoking the ILF theory.^25^ This model reverts the theoretical optics and inverts the starting energy of the ligands concerning the central metal since the four ligands act as a 6e^–^ donor rather than 8e^–^ to the gold, as occurs in compound **2** or **3**. In this regard, the fourth metal–ligand interaction could be better depicted as σ donation from the metal to the ligands. Thus, the metal constantly maintains unchanged its starting oxidation state of +1 never attains the classically expected Au(III). Even though the oxidation number is a formal assumption, in the case of CTCs, this new interpretation revises the reactivity toward oxidants of the linear gold(I) complexes in the perspective of the ILF theory.

## Experimental Section

### Materials

High-performance liquid chromatography (HPLC)-grade solvents, imidazoles, iodine (99%), and MeI (99%) were purchased from vendors. The reactions were led upon a flow of nitrogen and using dried solvents. The cyclotrimers were obtained by adding solid Ph_3_PAuCl to a −40 °C THF solution of the corresponding lithium imidazolate salt, followed by stirring, washing with water, with hexane, and crystallizing the raw product from CH_2_Cl_2_/hexane, following the procedure reported by Bonati et al.^8^

Crystals of HAuCl_4_·*n*H_2_O were obtained by storing, at 4 °C, a highly concentrated watery solution obtained by dissolving a chip of gold foil in aqua regia. The Ph_3_PAuCl was recovered as a microcrystalline solid from a suspension obtained by adding a double amount of PPh_3_ to a solution of HAuCl_4_ in ethanol.

### Characterization

Elemental analyses (C, H, N, S) were performed in-house with a Fisons Instruments Model 1108 CHNS-O elemental analyzer. Melting points were obtained using a Model SMP3 Stuart Scientific instrument. IR spectra were recorded from 4000 cm^–1^ to 600 cm^–1^ with a PerkinElmer SPECTRUM ONE System FT-IR instrument. The following IR annotations were used: br = broad, m = medium, s = strong, sh = shoulder, vs = very strong, w = weak and vw = very weak. ^1^H and ^13^C NMR spectra were recorded on an Oxford-400 Varian spectrometer (400.4 MHz for ^1^H and 100 MHz for ^13^C). Chemical shifts, in ppm, for ^1^H and ^13^C NMR spectra are relative to internal Me_4_Si. The following NMR annotations were used: br = broad, d = doublet, dd = double doublet, t = triplet, m = multiplet, s = singlet. Electrospray mass spectra (ESI-MS) were obtained in positive- or negative-ion mode on a Series 1100 MSD detector HP spectrometer, using an acetonitrile or methanol mobile phase. The compounds were added to reagent-grade acetonitrile to give solutions of an approximate concentration of 0.1 mM. These solutions were injected (1 μL) into the spectrometer via an HPLC HP 1090 Series II system that was fitted with an autosampler. The pump delivered the solutions to the mass spectrometer source at a flow rate of 300 μL min^–1^, and nitrogen was employed both as a drying gas and a nebulizing gas. Capillary voltages were typically 4000 and 3500 V for the positive- and negative-ion mode, respectively. Confirmation of all major species in this ESI-MS study was aided by a comparison of the observed and predicted isotope distribution patterns, the latter of which was calculated using the IsoPro 3.0 computer program.

### X-ray Structural Determination

The crystallographic data for compounds **2** and **3** were obtained by mounting a single crystal on glass fiber and transferring it to an APEX II Bruker CCD diffractometer. The APEX 3 program package^38^ was used to obtain the unit-cell geometrical parameters and for the data collection (30 s per frame scan time for a sphere of diffraction data). The raw frame data were processed using SAINT^38^ and SADABS^39^ to obtain the data file of the reflections. The structure was solved using SHELXT^39^ (intrinsic phasing method in the APEX 3 program). The refinement of the structures (based on F2 by full-matrix least-squares techniques) was performed using the SHELXTL-2014/7 program^40^ in the WinGX suite v.20142020.1.^41^ The H atoms were introduced in the refinement in defined geometry and refined “riding” on the corresponding carbon atoms. Crystallographic data were deposited with the Cambridge Crystallographic Data Centre as supplementary publication (CCDC reference code 2093399 for compound **2** and 2093397 for compound **3**). Copies of the data can be obtained free of charge on application to the CCDC, 12 Union Road, Cambridge CB2 1EZ, U.K. (fax, (+44) 1223 336033; e-mail, deposit@ccdc.cam.ac.uk).

### Computational Details

All the compounds were optimized at the DFT-B97D^42^ level of theory within the Gaussian16 package.^43^ All of the calculations were based on the CPCM model^44^ for the dichloromethane or iodomethane as the solvent, depending on the experimental conditions. The Triple Zeta basis set TZVP^45^ was used for all the atomic species, except for the Au and I atoms, for which the Stuttgart/Dresden (SDD) *pseudo*-potential^46^ was employed. All the optimized structures were validated as minima and/or transition states by computed vibrational frequencies. The contribution of each center to the molecular orbitals was estimated by using the AOMIX package.^47^ Cartesian coordinates, as well as the energetic features of all of the optimized structures, are reported in section 4 in the Supporting Information.

#### Reaction of [μ–Au-C^2^,N^3^-1-methylimidazolate]_3_ with Solid Iodine. Preparation of Compound **1** and **2**

The [Au(μ-C^2^,N^3^-1-methyl-imidazolate)]_3_ (30 mg; 0.036 mmol) was dissolved in 2 mL of dry CH_2_Cl_2_, under nitrogen atmosphere, and solid iodine (45 mg; 0.178 mmol) was added under magnetic stirring at room temperature. The initial colorless solution turned to brown within 10 min. The solution was evaporated to dryness, the tarry solid was washed with hexane (6 × 2 mL) and dissolved in hot THF (10 mL) to obtain an orange solution. Upon slow cooling of the THF solution at room temperature, orange needle-shaped crystals were fastly formed while, from the mother liquor, needles mixed with some platelets were obtained; both types of crystals are sparingly soluble in organic solvents.

#### Characterization Needles, Compound **1**·THF. Yield 38%

^1^H NMR (δ, room temperature, DMSO-*d*^6^): 7.74 (d, ^3^J_H–H_ = 1.5 Hz, compound **1**), 7.69 (m), 7.66 (d, ^3^J_H–H_ = 1.5 Hz, compound **2**), 7.62 (d, ^3^J_H–H_ = 1.5 Hz, compound **2**), 7.55 (d, ^3^J_H–H_ = 1.5 Hz, compound **2**), 7.40 (d,^3^J_H–H_ = 1.5 Hz, compound **1**), 7.38 (d, ^3^J_H–H_ = 1.5 Hz, compound **2**), 7.35 (d, ^3^J_H–H_ = 1.5 Hz, compound **2**), 7.07 (d, ^3^J_H–H_ = 1.5 Hz, compound **2**), 3.84 (s, compound **2**), 3.81 (s, compound **1**), 3.79 (s, compound **2**), 3.71 (s), 3.67 (s, compound **2**), 3.61 (m, 2H, THF), 1.77 (m, 2H, THF).

^13^C NMR (δ, room temperature, DMSO-*d*^6^): 166.1 (C2), 131.38, 130.88, 127.75, 125.91, 125.22, 122.95, 37.20, 36.78, 36.59.

MIR (cm^–1^): 3147 (w), 3126 (m), 2968 (m), 2936 (m), 2853 (m), 1554 (m), 1540 (m), 1461 (m), 1455 (m), 1436 (m, sh), 1409 (m), 1393 (m, sh), 1386 (m), 1361 (m), 1348 (m, sh), 1336 (w), 1323 (m), 1282 (m), 1159 (s), 1133 (m), 1084 (m), 1061 (m), 1028 (w), 965 (m), 903 (m), 861 (w), 825 (w), 725 (s).

FIR (cm^–1^): 692 (m), 667 (s), 648 (m, sh), 635 (m), 615 (m), 449 (s), 431 (m), 418 (w), 377 (w), 351 (w, sh), 338 (m), 311 (m), 301 (w, sh), 278 (m), 263 (w), 249 (m), 216 (w), 203 (m), 192 (s), 182 (m, sh), 174 (m), 164 (m), 154 (m), 149 (m), 140 (m), 133 (m), 116 (m).

Elemental analysis for C_12_H_15_Au_3_I_6_N_6_ + THF calcd %: C 11.52, H 1.39, N 5.04. Found %: C 12.00, H 1.32, N 5.24.

#### Characterization Platelets (Compound **2**·THF). Yield 24%

^1^H NMR (δ, room temperature, DMSO-*d*^6^): 7.66 (d, ^3^J_H–H_ = 1.5 Hz, 2H), 7.62 (d, ^3^J_H–H_ = 1.5 Hz, 2H), 7.55 (d, ^3^J_H–H_ = 1.5 Hz, 2H), 7.38 (d, ^3^J_H–H_ = 1.5 Hz, 2H), 7.35 (d, ^3^J_H–H_ = 1.5 Hz, 2H), 7.07 (d, ^3^J_H–H_ = 1.5 Hz, 2H), 3.84 (s, 3H), 3.79 (s, 3H), 3.67 (s, 3H), 3.61 (m, 2H, THF), 1.77 (m, 2H, THF).

^13^C NMR (δ, room temperature, DMSO-*d*^6^): 166.09 (C2), 131.39, 130.90, 127.76, 125.92, 125.23, 122.96, 67.50 (THF), 37.20, 36.79, 36.60, 25.60 (THF).

MIR (cm^–1^): 3153 (w), 3125 (m), 3112 (m), 3073 (w), 3064 (w), 2969 (m), 2939 (m), 2924 (m), 2862 (m), 1586 (w), 1562 (w), 1554 (w), 1541 (m), 1456 (s), 1445 (m), 1403(m), 1382 (m), 1371 (m), 1356 (w), 1337 (w), 1316 (m), 1302 (w), 1280 (m), 1157 (s), 1134 (m, sh), 1081 (m), 1061 (m), 1025 (w), 961 (m), 920 (m), 897 (m), 844 (w), 743 (m, sh), 732 (s), 718 (m, sh).

FIR (cm^–1^): 692 (s), 686 (s), 670 (s), 658 (m, sh), 645 (m, sh), 618 (m), 609 (m), 446 (s), 428 (m, sh), 418 (w), 397 (w), 337 (m), 307 (m), 278 (m), 253 (m), 249 (m), 244 (m), 227 (w), 201 (s), 192 (s), 182 (m, sh), 174 (m), 164 (m), 154 (m), 149 (m), 140 (m), 133 (m), 116 (m).

Elemental analysis for C_12_H_15_Au_3_I_4_N_6_ + THF calcd %: C 13.59, H 1.64, N 5.94. Found %: C 13.05, H 1.32, N 5.54.

#### Reaction of [μ–Au-C^2^,N^3^-1-methylimidazolate]_3_ with an Excess of Methyl Iodide. Preparation of Compound **3**

The [Au(μ-C^2^,N^3^-1-methyl-imidazolate)]_3_ (30 mg; 0.036 mmol) was dissolved in 2 mL of CH_3_I (excess), under a nitrogen atmosphere, and the solution was stirred at room temperature for 6 h in the darkness. After 3 h of stirring by monitoring the reaction by ^1^H NMR, the signal of compound **3** is 8% of those of the starting CTC. The pale yellow solution was layered with hexane and, upon storing at 5 °C for 1 week, yellow crystals were obtained. Yield = 36%.

MIR (cm^–1^): 3165 (w), 3141 (w), 3118 (w), 3007 (w), 2977 (w), 2933 (w), 2904 (w), 1657 (w), 1645 (w), 1628 (w), 1556 (w), 1536 (w), 1455 (m, sh), 1444 (s), 1400 (m), 1380 (m), 1317 (w), 1302 (w), 1282 (m), 1196 (m); 1154 (s), 1131 (m, sh), 1077 (m), 1024 (m), 968 (w), 828 (w), 816 (w), 733 (m, sh), 721 (s).

FIR (cm^–1^): 696 (s), 676 (s), 618 (w), 537 (w), 520 (w), 446 (s), 339 (m), 300 (m), 280 (m), 274 (m), 251 (m), 245 (m), 236 (m), 229 (m, sh), 218 (w), 205 (w), 199 (w), 192 (w), 177 (w), 152 (s), 141 (m, sh), 135 (m), 123 (m), 117 (w), 109 (m).

Elemental analysis for C_13_H_18_Au_3_IN_6_ calcd %: C 16.00, H 1.86, N 8.61. Found: C 16.51, H 1.79, N 8.51.

#### Reaction of [Au-μ-C^2^,N^3^-1-benzylimidazolate]_3_ with an Excess of Methyl Iodide. Preparation of Compounds **4** and **5**

The [Au(μ-C^2^,N^3^-1-benzyl-imidazolate)]_3_ (30 mg; 0.028 mmol) was dissolved in 2 mL of CH_3_I (excess), under a nitrogen atmosphere, and the solution was stirred at room temperature for 6 h in the darkness. The pale yellow solution was concentrated to 1 mL and layered with hexane. A microcrystalline solid was obtained after 12 h at 5 °C and the microscope inspection revealed the presence of at least two different types of crystals. The solid was strongly emissive in the yellow range upon 366 nm irradiation.

^1^H NMR (δ, room temperature, DMSO): 7.63 (d, ^3^J_H–H_ = 2 Hz, compound **4**), 7.56 (d, ^3^J_H–H_ = 2 Hz, compound **5**), 7.53 (d, ^3^J_H–H_ = 2 Hz, compound **4**), 7.48 (d, ^3^J_H–H_ = 2 Hz, 2H, compound **5**), 7.39 (s, broad, 2H, compound **5**), 7.38 (s, broad, 4H compound **5**), 7.36–7.28 (m, benzyl groups, compound **4** and compound **5**), 5.38 (s, 2H, compound **4**), 5.35 (s, 4H, compound **5**), 3.81 (s, N–CH_3_, compound **3**), 3.78 (s, NCH_3_, compound **5**).

^13^C NMR (δ, room temperature, acetone-*d*^6^): 184.4 (s, C2Im compound **4**), 181.0 (s, C2Im, compound **5**), 136.9 (s, Cypso, compound **4**) 136.6 (s, Cypso, compound **5**), 129 (s, compound **4**), 128.8 (s, compound **4**), 128.2, 127.9, 127.6, 123.5, 122.8, 122.2, 121, 54.1 (CH_2_–Bz, compound **4**), 53.9 (2CH_2_–Bz, compound **5**), 37.5 (NCH_3_, compound **4**), 37.1 (2NCH_3_, compound **5**).

MIR (cm^–1^): 3145 (w), 3114 (w), 3087 (w), 3069 (w), 3053 (w), 3028 (w), 3004 (w), 2954 (w), 2938 (w), 2886 (w), 2851 (w), 1687 (w), 1586 (w), 1557 (m), 1497 (m), 1468 (m), 1452 (m), 1404 (m), 1367 (m), 1330 (m), 1304 (m), 1216 (m), 1199 (m), 1155 (w), 1119 (m), 1078 (m), 1035 (w, sh), 1029 (m), 953 (w), 917 (w), 834 (m), 778 (m), 742 (s), 728 (s).

FIR (cm^–1^): 698 (s), 682 (w), 670 (w), 613 (m), 585 (m), 472 (m), 457 (m), 329 (w), 318 (w), 303 (w), 287 (w. sh), 278 (m), 259 (m), 250 (m, sh), 224 (w), 218 (w), 208 (m, sh), 201 (s), 185 (w), 174 (w), 168 (w), 160 (w), 151 (s) 131 (m), 124 (m), 113 (w).

ESI (−) (CH_3_OH, *m*/*z*, relative intensity): 450 (35) [AuI_2_]^−^. ESI (+) (CH_3_OH, *m*/*z*, relative intensity): 541 (100) [bis(1-benzyl-3-methyl-2yl-imidazolyl)-Au]^+^.

Elemental analysis for C_30_H_36_Au_3_I_3_N_6_ (compound **4** + compound **5** in 1:1 molar ratio) calcd %: C 26.63, H 2.44, N5.65. Found: C 26.82, H 2.50, N 5.51.

## Supporting Information Available

Supplementary figures, Cartesian coordinates, and energy features of optimized structures (PDF)

## Accession Codes

CCDC 2093397 and 2093399 contain the supplementary crystallographic data for this paper. These data can be obtained free of charge via www.ccdc.cam.ac.uk/data_request/cif, or by emailing data_request@ccdc.cam.ac.uk, or by contacting The Cambridge Crystallographic Data Centre, 12 Union Road, Cambridge CB2 1EZ, UK; fax: +44 1223 336033.
