# Supplementary material for: A Reinterpretation of the Imidazolate Au(I) Cyclic Trinuclear Compounds Reactivity with Iodine and Methyl Iodide with the Perspective of the Inverted Ligand Field Theory
Source: Inorg Chem. 2022 Feb 15;61(8):3527–39. doi: 10.1021/acs.inorgchem.1c03492 (PMC8889582; doi:10.1021/acs.inorgchem.1c03492)
Supplement: Supplementary file 1 — ic1c03492_si_001.pdf [file ic1c03492_si_001.pdf]

A reinterpretation of the Imidazolate Au(I) Cyclic Trinuclear Compounds reactivity with iodine and methyl iodide with the perspective of the Inverted Ligand Field Theory.

Rossana Galassi,[1,*] Lorenzo Luciani,[1] Claudia Graiff,[2] Gabriele Manca[3,*]

[1]School of Science and Technology, Chemistry Division, University of Camerino, Via Sant'Agostino, 1, I-62032, Italy

[2] Department of Chemistry, Life Sciences and Environmental Sustainability, Università degli Studi di Parma, Parco Area delle Scienze 17/A, 43124 Parma, Italy

[3]Istituto di Chimica dei Composti Organo-Metallici, CNR-ICCOM, 50019, Sesto Fiorentino, Italy

Supporting Info

List of contents

1 Preparations
2 IR Spectra
3 NMR spectra
4 Computational studies
5 Crystallographic files

1 Preparations

1.1 Reaction of [μ-Au-C^2,N^3-1-methylimidazolate]$_3$ with Hydrochloric Acid. Preparation of compound [(Im^{Me}-NHC-2yl)$_2$-Au]Cl. Compound 6.

The *[Au(μ-C^2,N^3-1-Methyl-imidazole)]$_3$* (40 mg; 0.048 mmol) was dissolved in 4 ml of CH_2Cl_2, under nitrogen atmosphere, and 1 mL of a 1M watery solution of HCl (0.5 mmol) was added under vigorous magnetic stirring at room temperature for 1.5 Hour. The suspension was extracted with water (3 times), dried under Na_2SO_4. The solution turned pale pink and after filtration, was dried under a vacuum. The white solid was washed with cyclohexane and evaporated to dryness to obtain a microcrystalline. Yield 56%

^{1}H NMR (δ, room temperature, $CDCl_3$): 11.12 (s, broad, 2H), 7.09 (t, $^3J_{H-H}$ = 1.6Hz, 2H), 7.00 (t, $^3J_{H-H}$ = 1.6Hz, 2H), 3.85 (s, 6H, NCH_3), 1.44 (s, trace of cyclohexane).

^{13}C NMR (δ, room temperature, $CDCl_3$): 167 (C2), 121(C4), 118 (C5), 38.4 (N-CH_3).

MIR (cm^{-1}): 3293 (m, br), 3174 (m), 3145 (w), 3130 (w), 3012 (w), 2957 (m, sh), 2922 (m), 2853 (m), 1665 (w), 1575 (m), 1559 (m, sh), 1489 (w), 1452 (s), 1443 (m, sh), 1394 (m), 1348 (m), 1332 (m), 1312 (m, sh), 1281 (m), 1235 (m), 1127 (m, sh), 1098 (s), 1072 (s), 1009 (m), 969 (m,sh), 946 (m, sh), 922 (m), 893 (m, sh), 875 (m, sh), 832 (m), 726 (s).

FIR (cm^{-1}): 699 (w), 682 (w), 662 (m), 637 (m), 613 (m), 415 (w), 329 (s), 322 (s), 303 (w), 265 (s), 256 (m, sh), 239 (m) 230 (m), 221 (m), 215 (m), 206 (m), 200 (m, sh), 185 (s), 177 (s), 166 (m), 147 (m, sh), 139 (s), 123 (s), 112 (s), 103 (s).

Elemental analysis for $C_8H_{12}AuClN_4$ calcd %: C 24.23, H 3.05, N 14.13. Found: C 24.68, H 3.43, N 13.52.

The reaction of [μ-Au-C^2,N^3-1-benzylimidazolate]$_3$ with Hydrochloric Acid. Preparation of compound [Im^{Bz}-NHC-2yl)$_2$-Au]Cl. Compound 7.

The *[Au(μ-C^2,N^3-1-Benzyl-imidazole)]$_3$* (40 mg; 0.038 mmol) was dissolved in 3.5 ml of CH_2Cl_2, under nitrogen atmosphere, and 1 mL of a 1M watery solution of HCl (0.5 mmol) was added under vigorous magnetic stirring at room temperature for 1.5 Hour. The suspension was extracted with water (3 times), dried under Na_2SO_4, and after filtration, evaporated to dryness. A microcrystalline solid was obtained. Yield 81%

^{1}H NMR (δ, room temperature, DMSO): 12.43 (s, broad, 2H), 7.65-7.42 (m, 14H), 5.41 (s, 4H).

^{13}C NMR (δ, room temperature, DMSO): 180 (C2), 137 (Ph), 128 (Ph), 127.92 (Ph), 127.50 (Ph), 121.50 (C4), 119.52 (C5), 53.4 (CH2-Ph).

Elemental analysis for $C_{20}H_{22}AuClN_4$ calcd %: C 43.61, H 4.03, N 10.17. Found: C 44.02, H 3.83, N 9.72.

2 IR spectra

Crystals of compounds 1 and 2 are simultaneously formed as needles and platelets, respectively. Manual separation of the crystals was performed, and the IR spectra were recorded.

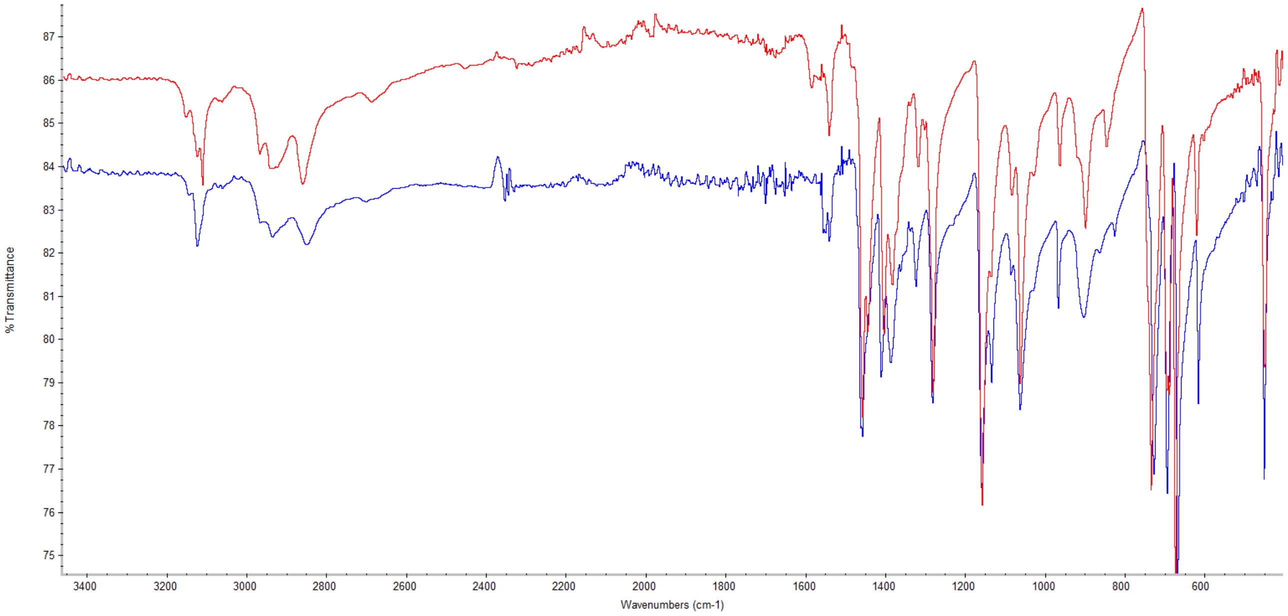

Figure S1. Overlapped IR spectra in the range 4000-400 cm^{-1} of compound **1** (red lines, needles) and compound **2** (blue line, platelets)

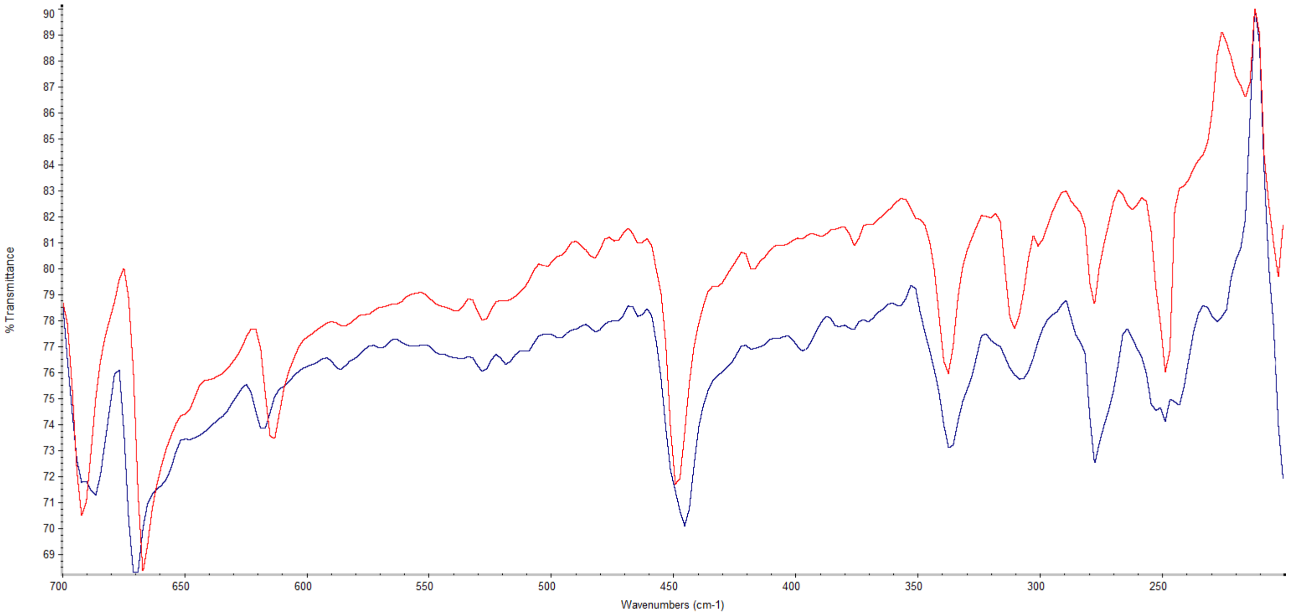

Figure S2. Overlapped IR spectra in the range 700-200 cm^{-1} of compound **1** (red line, needles) and **2** (blue line, platelets)

3 NMR spectra

Crystals of compounds **1** and **2** are simultaneously formed as needles and platelets, respectively. Manual separation of the crystals was performed and the ^{1}H and ^{13}C NMR spectra were recorded in DMSO-d^{6}. Both compounds are sparingly soluble in DMSO-d^{6}. Compound **1** is less soluble than compound **2**. The NMR spectra consist of several peaks but none of the presents can be attributed to the starting CTC^{Me}. The solution of compound **1** in DMSO-d^{6} displays signals for the imidazole protons at 7.74 and 7.40 ppm, and 125.27 and 122.99 ppm, respectively, attributed to compound **1** but, predominantly, the signals due to compound **2** were observed (see figure S2-S5). It is likely that the fully iodized compound **1**, upon the difficult dissolution, loses iodine to give compound **2**. Moreover the overnight acquisition for the ^{13}C NMR results in the rising of traces of additional compounds, likely due to the nine membered cycle rupture and the formation of carbene species (in example the signal at 13 ppm in ^{1}H NMR figure S4, acquired after 12 hours from the preparation of the solution, and signals at 113 and 118 ppm in figures S5 and S6). The ^{1}H NMR spectrum of compound **2** consists of six sharp doublets of the same intensity for the imidazole protons and three singlets of the same intensity for the N-methyl groups, none of them corresponds to the starting CTC^{Me} or to the free 1-methylimidazole; saturated solutions of compounds **1** and **2** exhibit nearby small singlets for the C2 atoms, 166.13 and 166.09 ppm, respectively (see inset image in figure S7).

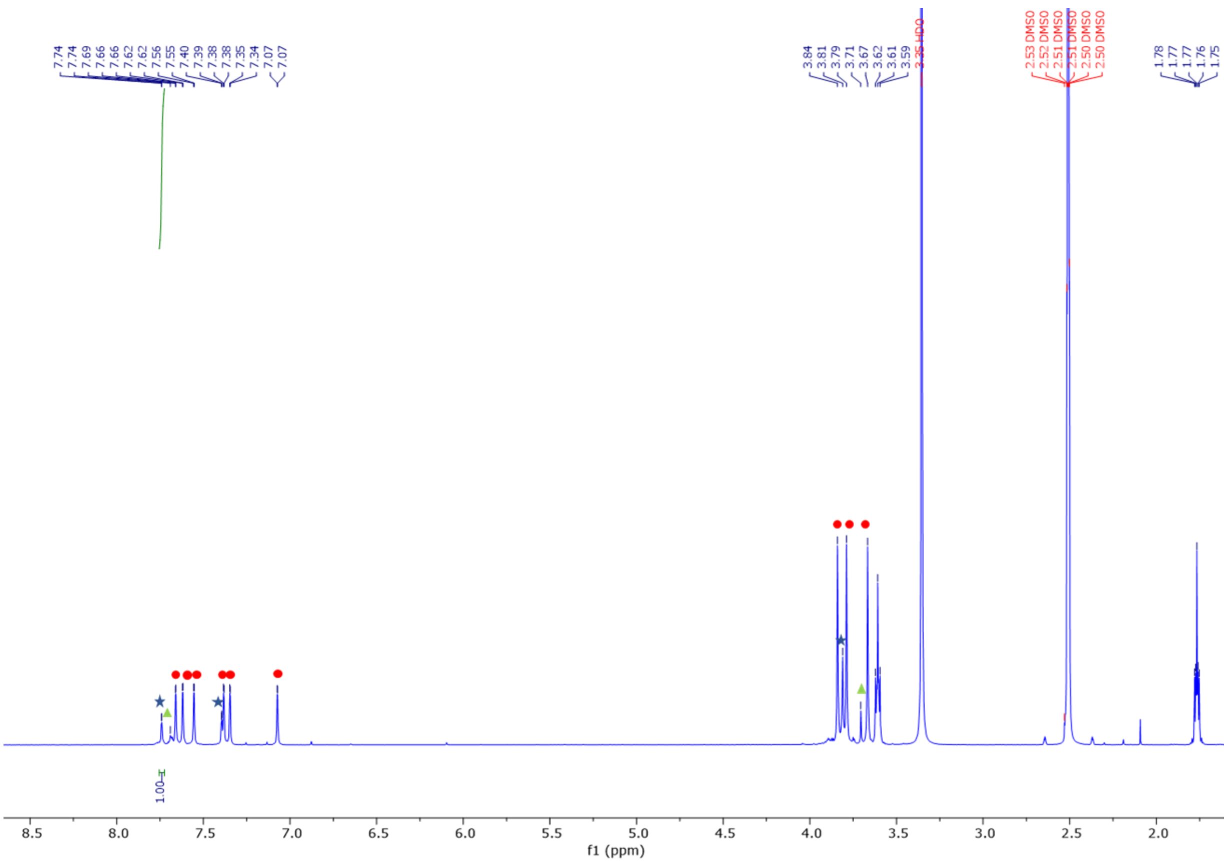

Figure S3. ^{1}HNMR spectrum of compound **1** in DMSO-d^{6}. The dissolution in DMSO-d^{6} was obtained by heating and sonication. Stars indicate peaks due to compound **1**, while red circles depict the formation of compound **2** in solution, due to the loss of an iodine molecule. Green triangles label peaks that are not assigned which rise upon a time. At 3.60 ppm and 1.76 ppm the signals of tetrahydrofuran.

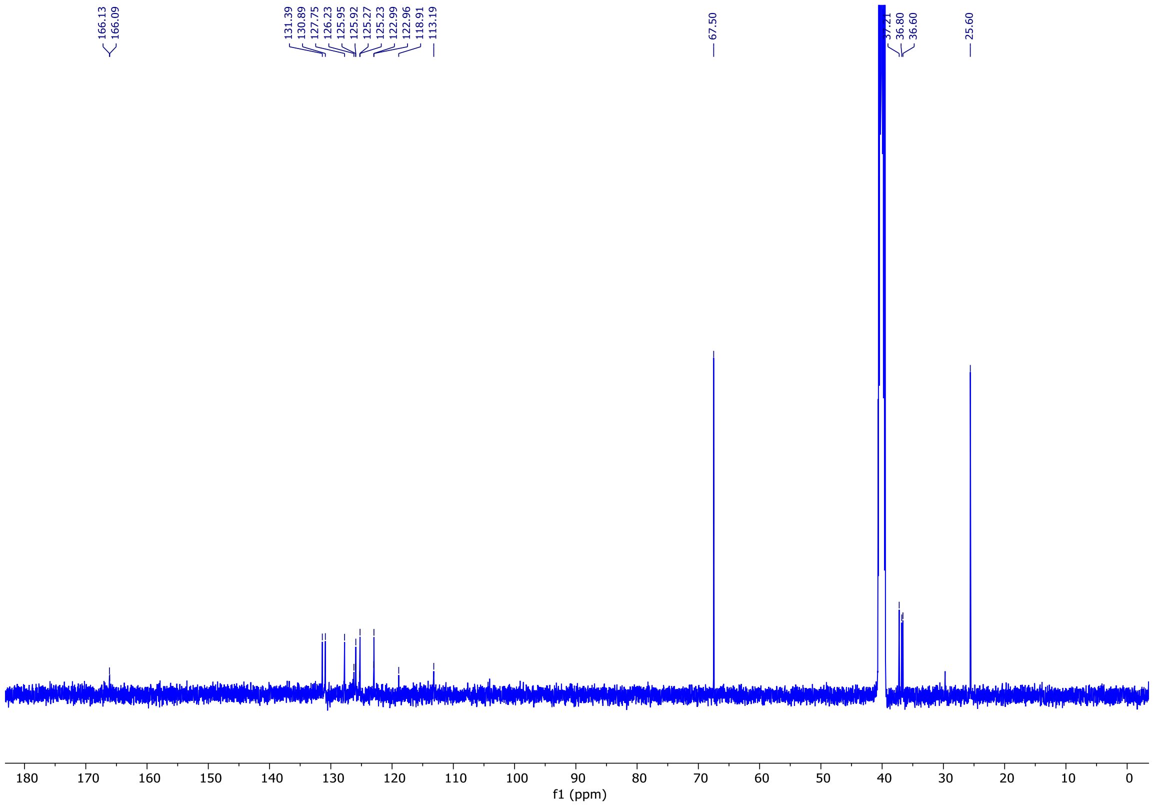

Figure S4. ^{13}C NMR spectra in DMSO-d^6 of compound **1** upon heating and sonication. The acquisition of the ^{13}C NMR spectrum needed an accumulation time of several hours. Most of the peaks are due to compound **2**.

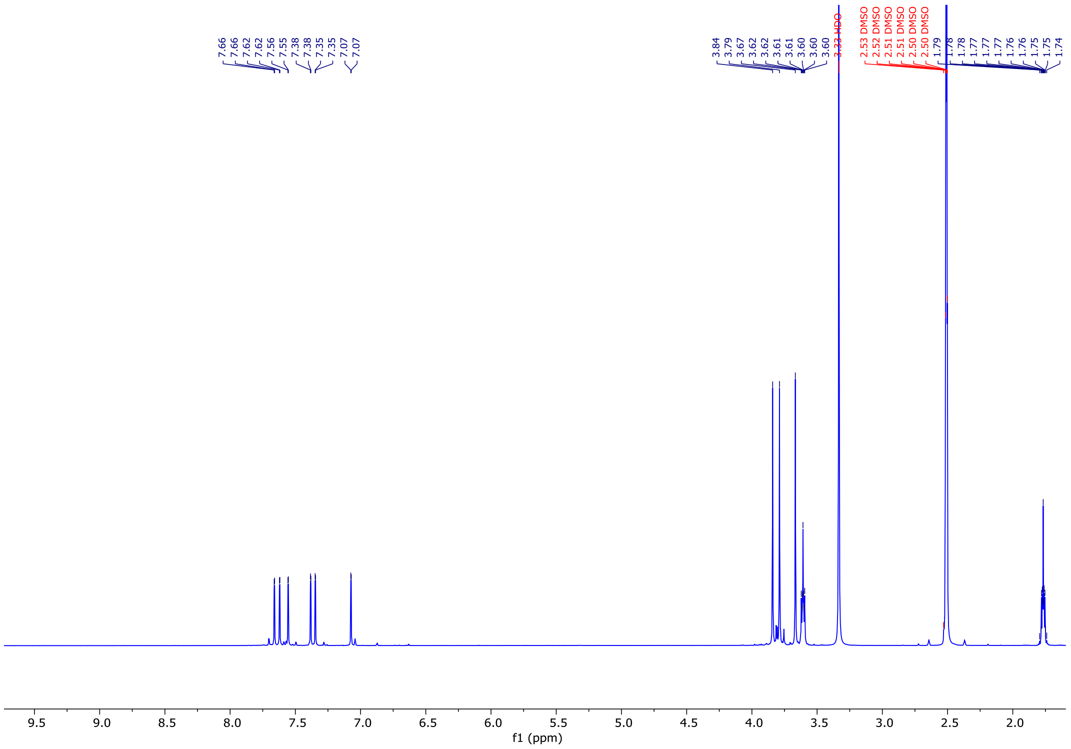

Figure S5. ^{1}HNMR spectrum of the sparingly soluble compound **2** in DMSO-d^6. The dissolution in DMSO-d^6 was obtained by heating and sonication. Small traces of compound **1** are present.

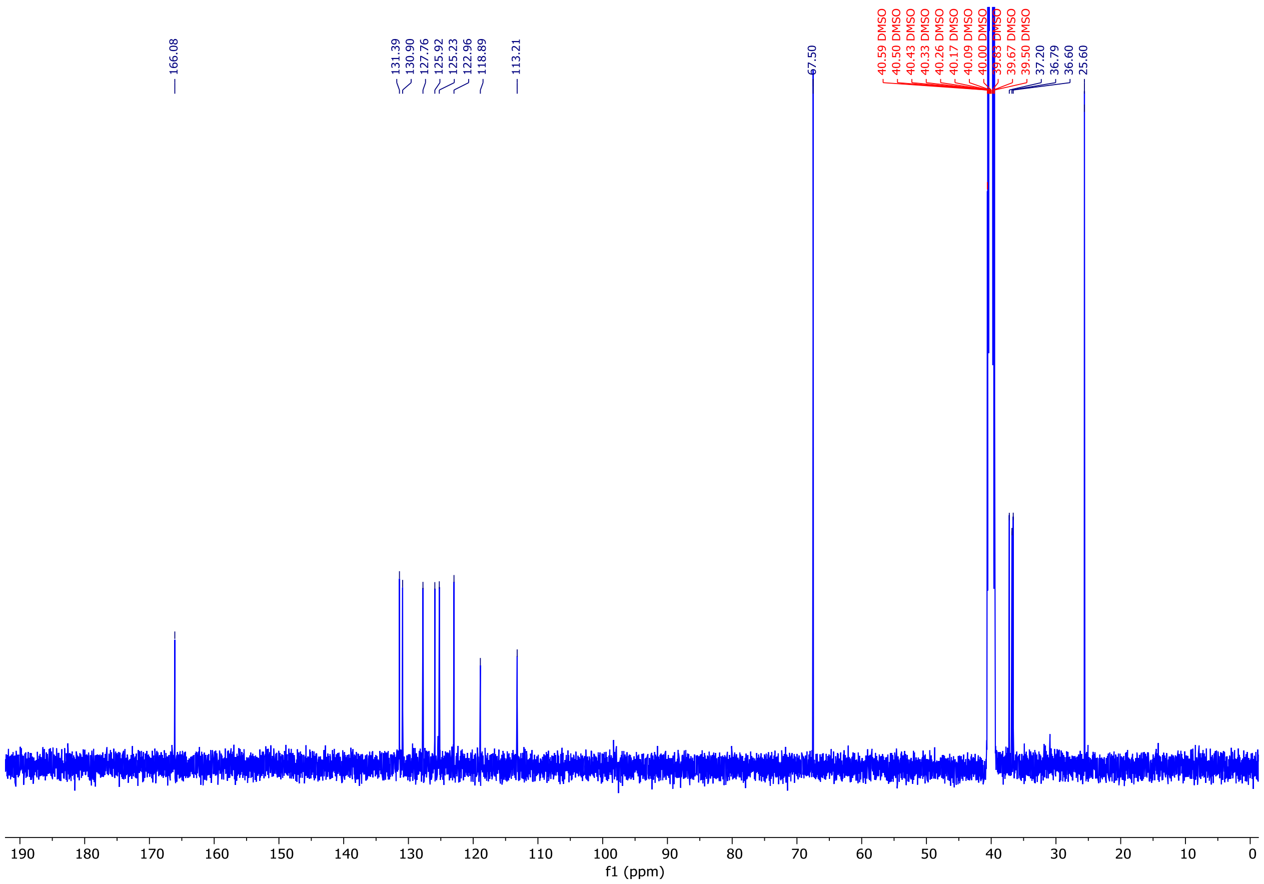

Figure S6. ^{13}C NMR spectrum in DMSO-d^6 of compound **2** upon heating and sonication. The acquisition of the ^{13}C NMR spectrum needed an accumulation time of several hours.

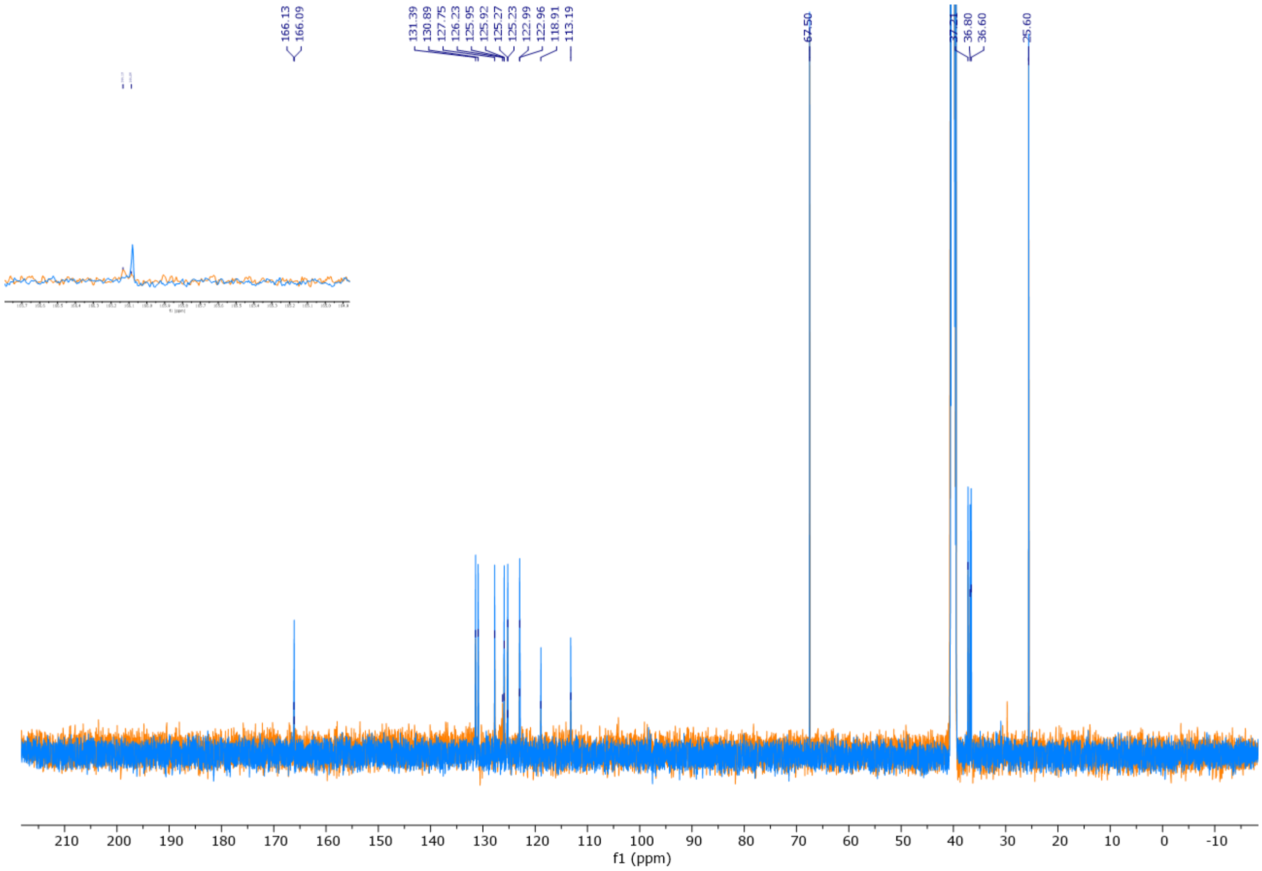

Figure S7. Overlapped ^{13}C NMR spectra in DMSO-d^6 of compounds **1** and **2**. In the inset image signals for the C2 of the imidazoles: in orange the signal due to the dissolution of compound **1** (166.13 ppm and 166.09 ppm) and the signal of the compound **2** (166.09 ppm). Compound **2** is present in the solution of compound **1** upon the dissociation of iodine from compound **1**.

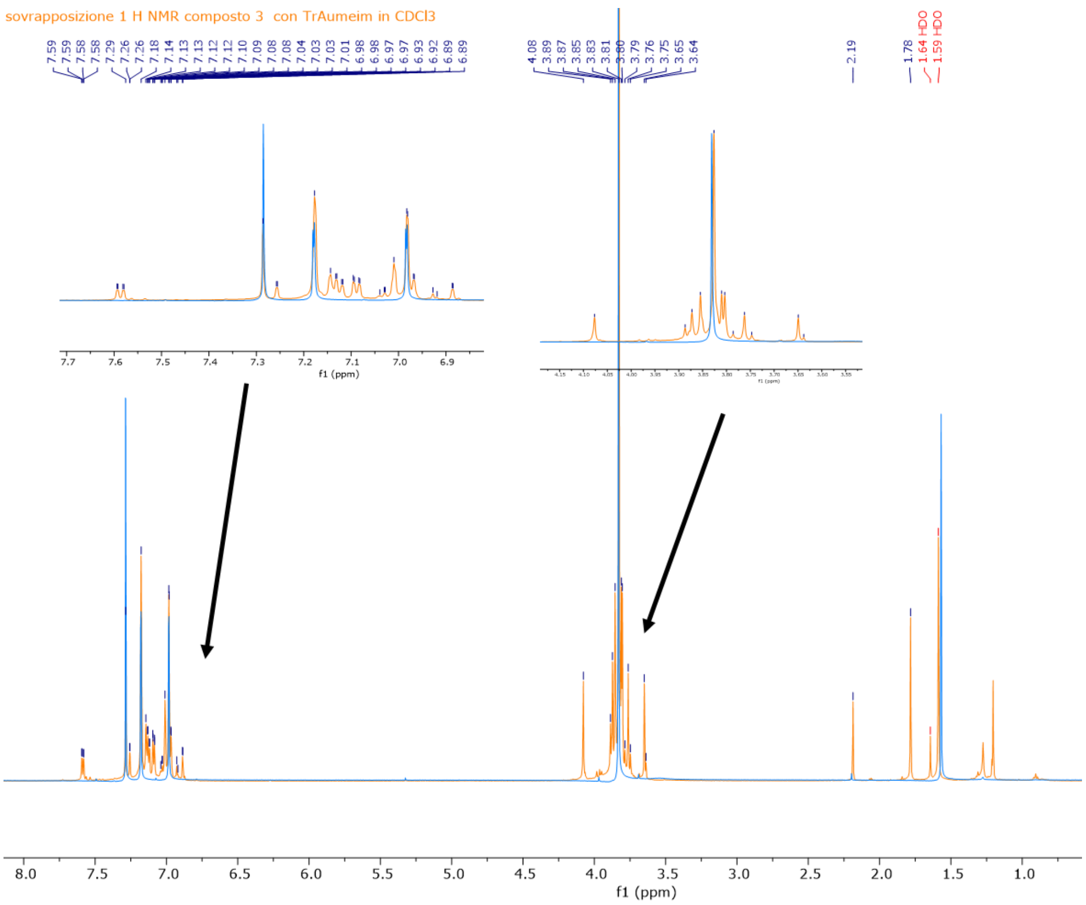

Figure S8. ^{1}HNMR spectrum in $CDCl_3$ of compound **3** (orange line) overlapped with the spectrum of CTC^{Me} (blue line). The signal at 2.19 ppm is the free MeI and the one at 1.78 ppm due to the CH_3-Au-I frame. Beyond the CTC^{Me} and compound **3**, also other species are generated in the solution. Inset images are enlargements of the chemical ranges indicated by the arrows. The attribution of the Me-Au-I at 1.78 ppm was made according to data reported in the literature.[1]

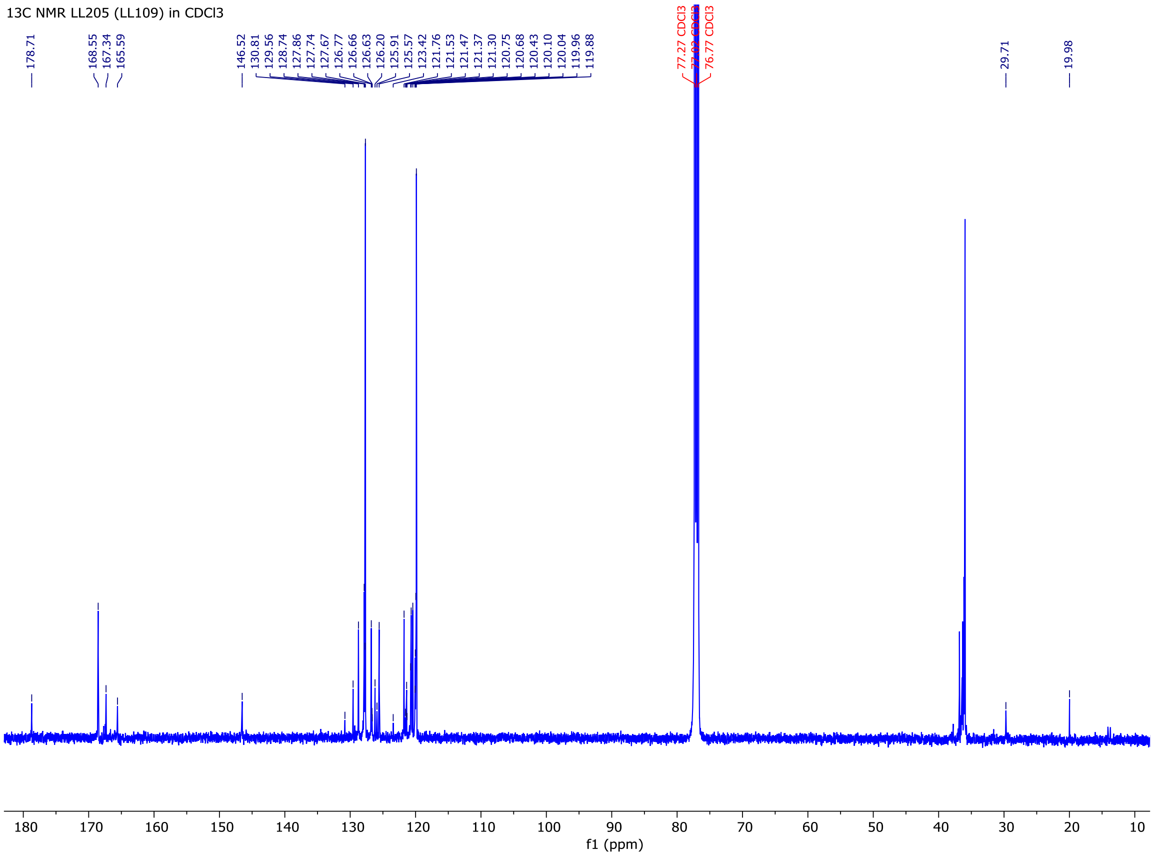

Figure S9. ^{13}C NMR spectrum in $CDCl_3$ of compound **3,** revealing the presence of CTC^{Me} and free MeI (29 ppm) in addition the compound 3, with methyl bound to Au at 20 ppm. Upon long accumulation, additional species are formed too.

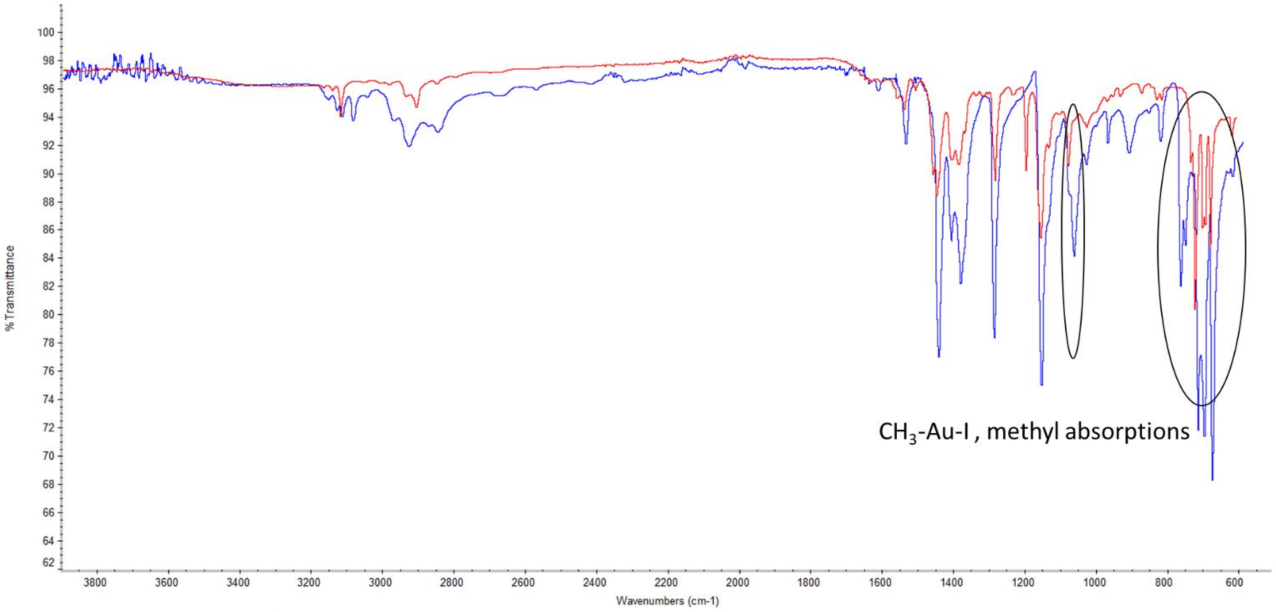

Figure S10. Overlapped IR spectra in the range 4000 - 600 cm^{-1} of the CTC^{Me} (red line) and compound **3** (blue line). Although a good overlap of the bands is mostly observed, additional absorptions are observed at 1154 cm^{-1} (CH_3 deformation) and 721 cm^{-1} (CH_3 rocking) in the spectrum

of compound **3,** likely due to the deformation and rocking modes of the Methyl of the Me-Au-I moiety. The attribution was made according to data reported in the literature.[2]

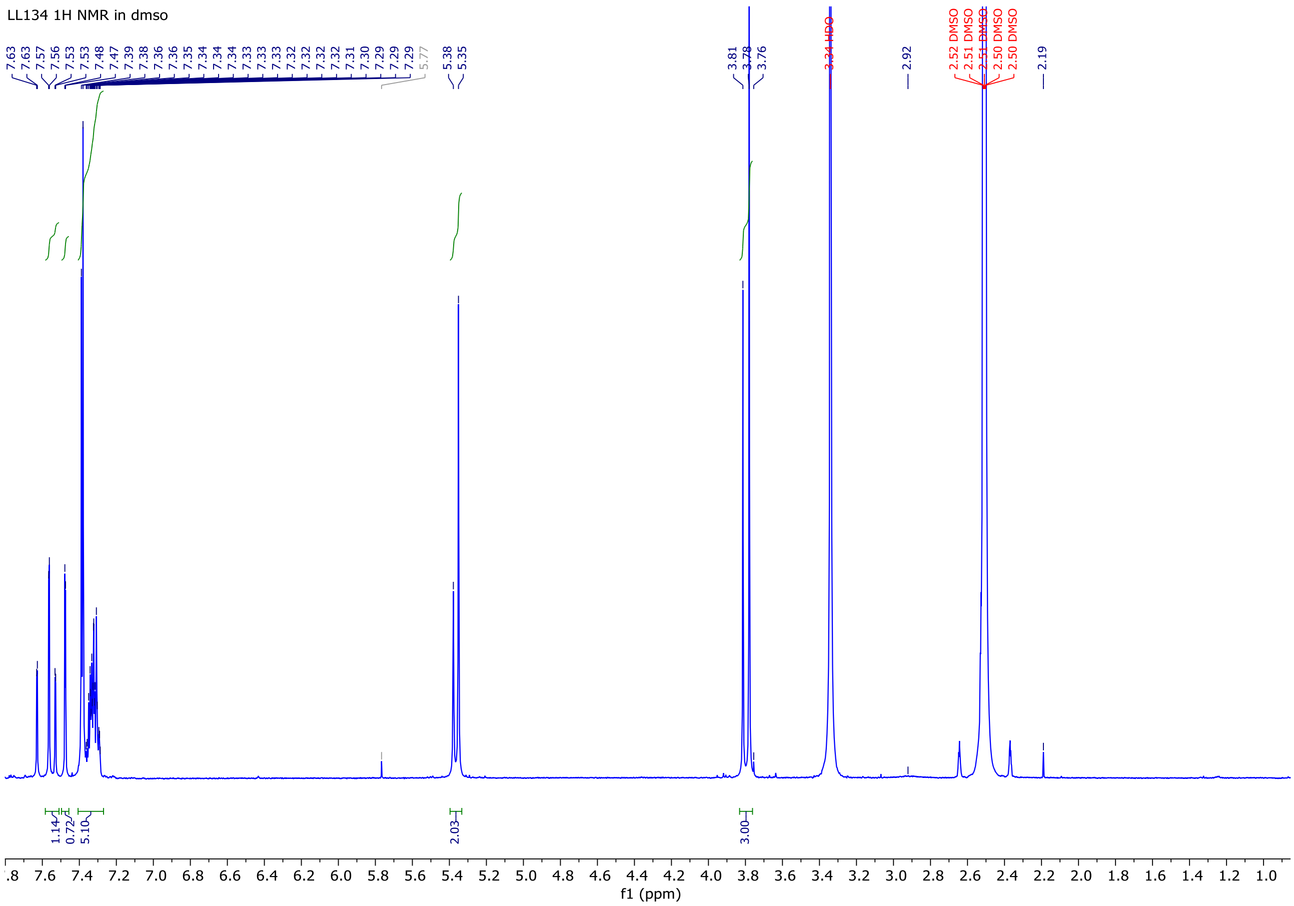

Figure S11. ^{1}H NMR spectrum of compounds **4** and **5** recorded in DMSO-d^6.

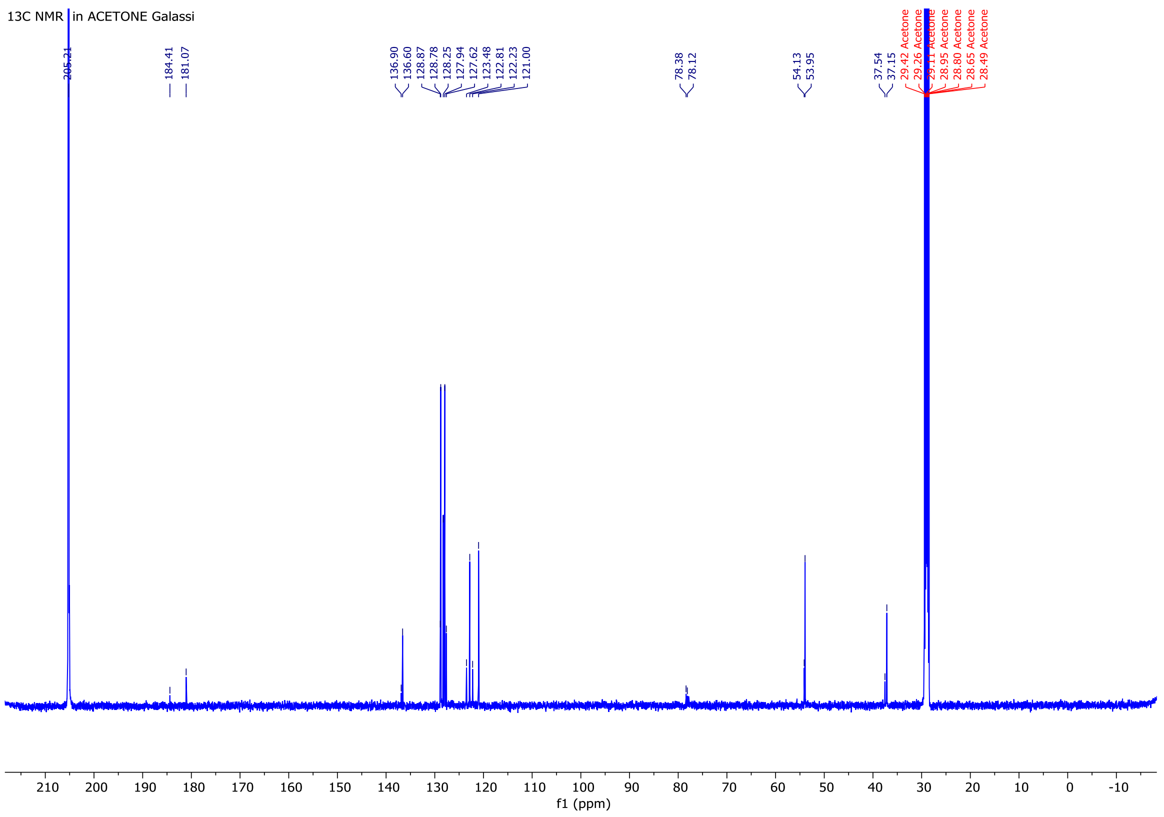

Figure S12. ^{13}C NMR spectrum recorded in acetone -d^6 for compounds **4** and **5**. The peaks at 28 and 206 ppm are due to DMSO, the peak at 77 is due to $CHCl_3$, the peak at 54 ppm is due to CH_2Cl_2.

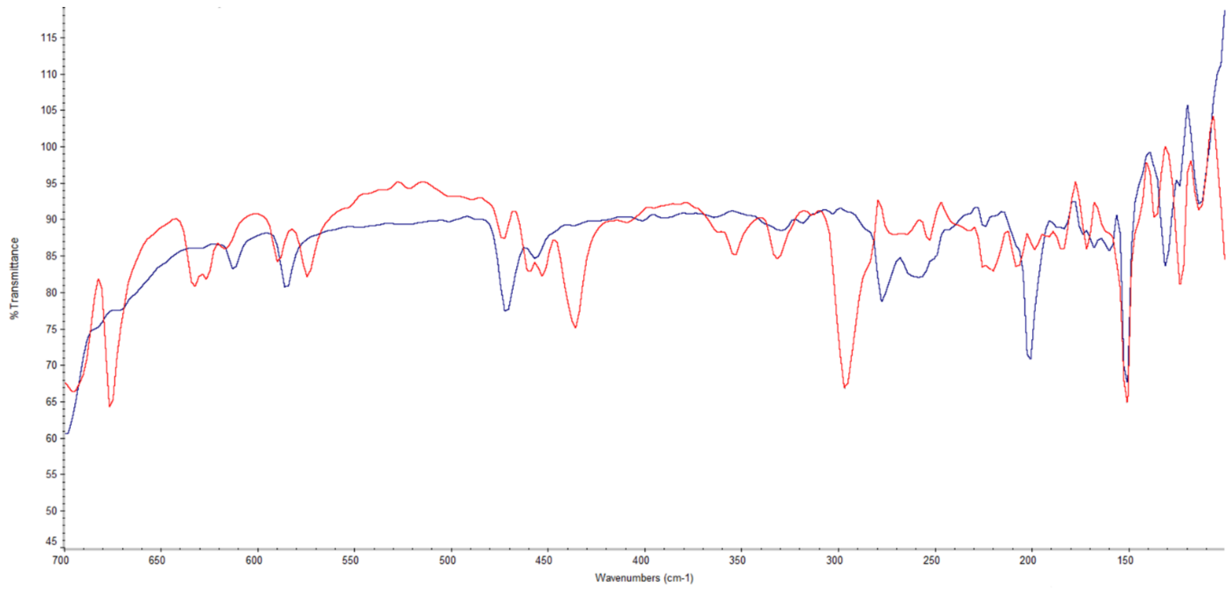

Figure S13. Overlapped FIR spectra of the solid consisting of compounds **4** and **5** (red line) and that of the starting CTC^{Bz} (blue line).

4 Computational studies

4.1 Reactivity of the CTC^{Me} with molecular I_2

As pointed out in the manuscript, the reactivity between **CTC^{Me}** and I_2 evolves through the formation of an initial adduct, **$CTC^{Me}*2I_2$**, held together with the trinuclear moiety and two I_2 molecules with a free energy gain of -11.8 kcal mol^{-1}. Then, a triiodide moiety is released with the achievement of cationic species **$CTC^{Me}I^+$** with a free energy cost +14.5 kcal mol^{-1} and, then, the triiodide species reacts with **$CTC^{Me}I^+$** to provide the first intermediate **$CTC^{Me}I_2$** together with a new I_2 molecule with a ΔG= -24.1 kcal mol^{-1}. **$CTC^{Me}I_2$** features a gold center in square planar coordination due to the formation of two new Au-I bonding. In the case of the methyl substituent at the imidazole ring, the process occurs stepwise for the two remaining metal centers. Briefly, a second adduct, **$CTC^{Me}I_2*2I_2$** is formed with a free energy gain of -9.2 kcal mol^{-1}, again a triiodide is released (ΔG= +12.2 kcal mol^{-1}) together with the formation of the cationic intermediate **$CTC^{Me}I_3^+$**. The complete iodination of the second gold, compound **2** is observed with a free energy gain of -19.3 kcal mol^{-1}. The third iodination follows similarly with a gain of -7.1 kcal mol^{-1} followed by +10.4 kcal mol^{-1} cost for the separation of the I_3^- and a further gain of -17.4 kcal mol^{-1} occurs for the achievement of the fully iodinated species, **1**. Scheme S1 summarizes the overall free energy pathway associated with the reactivity of **CTC^{Me}** with di-iodine up to the final species **1**.

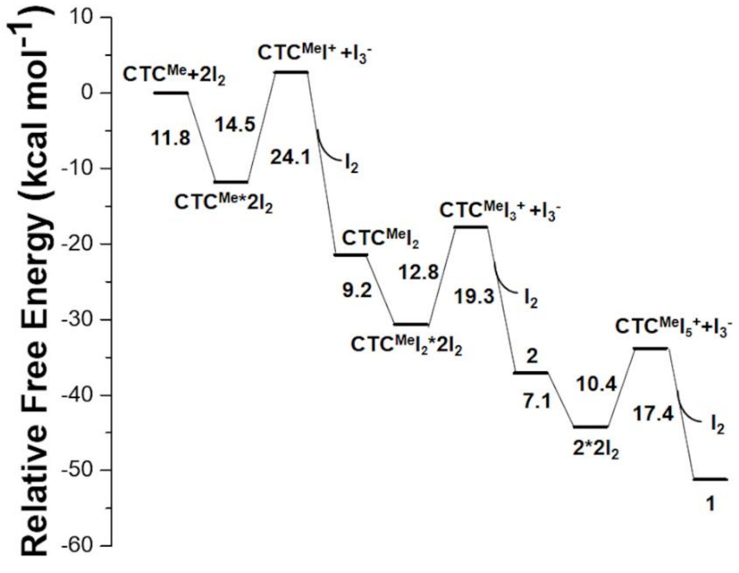

Scheme S1. Free Energy pathway for the reaction between the **CTC^{Me}** and di-iodine molecules up to the final complete iodinated species **1**.

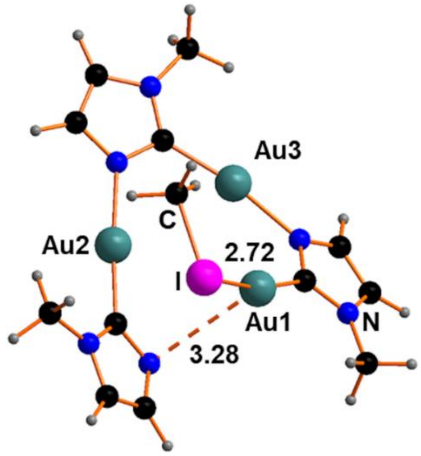

Figure S14. Optimized structure of compound **11^{Me}**.

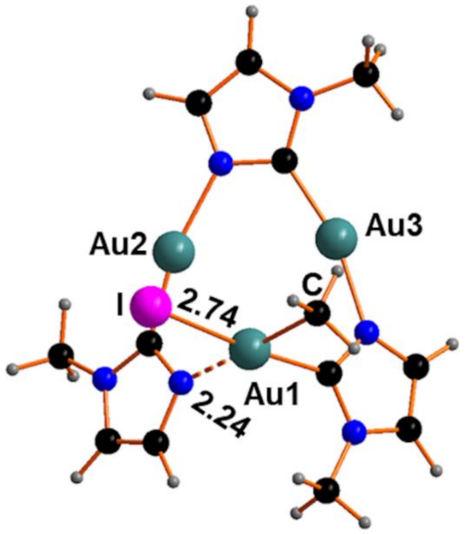

Figure S15. Optimized structure of compound **12^{Me}**.

4.2 Computational studies on the reaction of HCl with CTC^{Me} or CTC^{Bz}

The reactivity between the original CTCs with methyl iodide highlighted the not innocent behavior of the imidazole ligand and their ability to catch a methyl cation. Thus, we wonder if such a discrepancy is also operative for the reaction with the hydrochloric acid or not. Once again, the investigation of direct involvement in the reaction is excluded in both cases while the analysis revealed again that the incoming substrate reacts with the aromatic system of the imidazolyl ring. The computational analysis perfectly mirrors the experimental results since in both cases the formation of carbene and bis-carbene moieties is observed. Figure S16 shows the Transition State for the reaction of starting compound with benzyl, Figure S16a, and with methyl substituents, Figure S16b.

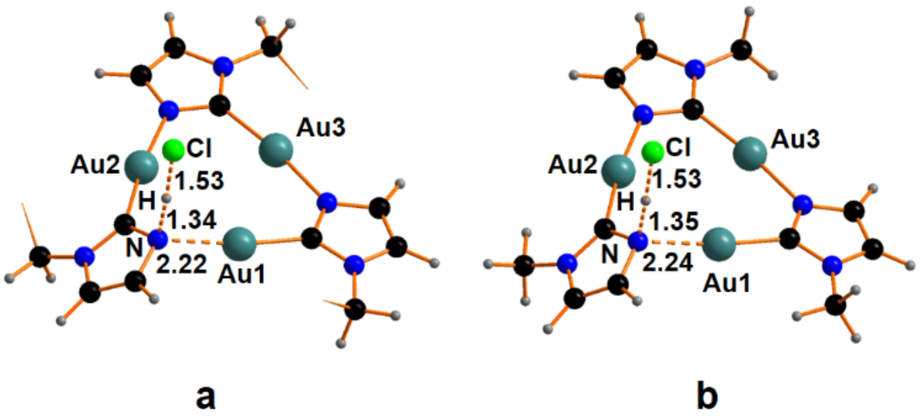

Figure S16. Optimized structure of Transition State for the interaction between HCl and: a) **CTC^{Bz}** and b) **CTC^{Me}**. The aryl rings are hidden for clarity in Figure S16a.

In both cases, the free energy barriers associated with the formation of the carbene and the cleavage of the Au1-N bonding are very small, being not higher than 7 kcal mol^{-1}. The structures feature an already stretched Au1-N bonding and the transferred proton in between the chlorine and the nitrogen center. In both cases, the H-Cl distance of the incoming hydrochloric acid is elongated by *ca.* 0.24 Å, suggesting an already advanced stage of the process. After such a Transition State the process evolves toward the formation of the N-H linkage of compound **CTC$_{HCl}$** with a free energy gain of -22.9 and -18.5 kcal mol^{-1} for **CTC^{Bz}** and **CTC^{Me}**, respectively. The somewhat higher energy gain in the case of the benzyl could be reasonably explained given a based on somewhat more pronounced stability of a benzyl carbene compared to the methyl one. The reaction proceeds in the same way with the final formation of three (NH-carbene)AuCl moieties with a total free energy gain of -26.3 and -23.2 kcal mol^{-1}.

4.3 Cartesian coordinates and free energies of all the structures optimized in the computational analysis (B97D-DFT level of theory).

Compound **CTC^{Me}** in dichloromethane solution

Cartesian Coordinates

Au -0.406798 3.325178 4.579262
Au -1.550532 1.905008 1.426366
Au 1.988424 2.602075 1.932564
N -3.438684 3.089687 5.027069
C -2.352157 2.911461 4.212505
N -2.816913 2.384612 3.044612
C -4.190218 2.241204 3.141064
C -4.587434 2.679064 4.374367
C -3.408463 3.648018 6.376832
N 3.785814 3.746060 4.147349
C 2.536750 3.391331 3.711717
N 1.670147 3.673655 4.725057
C 2.387658 4.203574 5.783646
C 3.708539 4.253095 5.432320

C 5.021247 3.628842 3.376359
N -0.317410 1.001130 -1.238427
C -0.153075 1.531148 0.013499
N 1.177803 1.782900 0.164291
C 1.831227 1.410090 -0.997785
C 0.906156 0.922683 -1.879502
C -1.593119 0.613997 -1.836131
H -4.775266 1.838471 2.325994
H -5.560596 2.735403 4.841360
H -3.873339 2.947133 7.079197
H -3.947049 4.602554 6.401210
H 1.903110 4.502144 6.702834
H 4.584968 4.593630 5.965529
H 5.747903 3.021737 3.927909
H 5.442436 4.623242 3.187917
H 2.901784 1.516578 -1.104436
H 1.001014 0.531624 -2.882663
H -2.356465 0.624115 -1.053490
H -1.511383 -0.393630 -2.257895
H 4.786689 3.145167 2.424435
H -1.871313 1.321061 -2.626714
H -2.364455 3.810264 6.657577

HF=-1202.8418163

Zero-point vibrational energy 689445.4 (Joules/Mol)

Zero-point correction= 0.262596 (Hartree/Particle)

Thermal correction to Energy= 0.285927

Thermal correction to Enthalpy= 0.286871

Thermal correction to Gibbs Free Energy= 0.205070

Sum of electronic and zero-point Energies= -1202.579220

Sum of electronic and thermal Energies= -1202.555889

Sum of electronic and thermal Enthalpies= -1202.554945

Sum of electronic and thermal Free Energies= -1202.636747

Compound **CTC**^{Bz} in dichloromethane solution

Cartesian Coordinates

N 5.041979 1.862971 8.672419
N 4.402015 -0.216667 8.938355
N 3.737431 -5.310583 8.768541
N 5.083677 -4.788132 7.119828
N 8.041032 -2.461059 3.625587
N 7.270020 -0.889526 4.945628
C 5.134925 0.605362 8.138912
C 4.268181 1.832349 9.819669
H 4.076822 2.722177 10.402378
C 3.868631 0.533673 9.973129
H 3.250434 0.079711 10.734738
C 5.782289 3.032832 8.190918
H 5.129210 3.908725 8.267743
H 5.995170 2.850533 7.131221
C 7.067007 3.260767 8.964908
C 8.047531 2.258055 9.026252
H 7.873236 1.308725 8.523242
C 9.234501 2.473975 9.731135

H 9.989067 1.690487 9.771722
C 9.454468 3.695037 10.383795
H 10.378685 3.861777 10.933842
C 8.480323 4.697292 10.327026
H 8.642299 5.646668 10.834105
C 7.290456 4.477981 9.621030
H 6.529233 5.256058 9.579599
C 4.340915 -4.258312 8.131014
C 4.095395 -6.500317 8.157843
H 3.723868 -7.453170 8.507242
C 4.935280 -6.164974 7.132451
H 5.442565 -6.795634 6.415791
C 2.788566 -5.198048 9.890274
H 2.646345 -6.213888 10.277002
H 1.825257 -4.844275 9.504419
C 3.278564 -4.268910 10.980540
C 4.554711 -4.432109 11.536878
H 5.186487 -5.250764 11.197436
C 5.032833 -3.524111 12.483717
H 6.030637 -3.650155 12.899490
C 4.233483 -2.448151 12.892708
H 4.609624 -1.735720 13.624205
C 2.953583 -2.290056 12.352492
H 2.329061 -1.454103 12.661597
C 2.481514 -3.195027 11.395765
H 1.501761 -3.051955 10.943777
C 7.273069 -2.234377 4.736264
C 8.508159 -1.259832 3.121750
H 9.130169 -1.214647 2.239124
C 8.029093 -0.287455 3.955282
H 8.174848 0.783391 3.926968
C 8.221393 -3.766121 2.986082
H 8.093715 -4.518710 3.773263
H 9.250099 -3.827114 2.615187
C 7.238287 -4.006877 1.855292
C 5.859394 -3.838165 2.056314
H 5.496748 -3.508073 3.027636
C 4.959152 -4.089608 1.017472
H 3.891738 -3.956108 1.183901
C 5.426641 -4.512625 -0.234364
H 4.724459 -4.706810 -1.042889
C 6.800040 -4.680981 -0.440605
H 7.171847 -5.004221 -1.411148
C 7.700764 -4.426047 0.601104
H 8.770702 -4.551630 0.439653
Au 6.212494 -0.003366 6.541202
Au 4.269961 -2.293166 8.605819
Au 6.251148 -3.583069 5.840126

HF=-1895.6943855

Zero-point vibrational energy 1313604.8 (Joules/Mol)

Zero-point correction= 0.500326 (Hartree/Particle)

Thermal correction to Energy= 0.536682

Thermal correction to Enthalpy= 0.537626

Thermal correction to Gibbs Free Energy= 0.423831

Sum of electronic and zero-point Energies= -1895.194060

Sum of electronic and thermal Energies= -1895.157704

Sum of electronic and thermal Enthalpies= -1895.156760

Sum of electronic and thermal Free Energies= -1895.270555

Compound **I_2** in dichloromethane solution

Cartesian Coordinates

I 0.000000 0.000000 -0.006327

I 0.000000 0.000000 2.73632

HF=-22.9138694

Zero-point vibrational energy 1168.4 (Joules/Mol)

Zero-point correction= 0.000445 (Hartree/Particle)

Thermal correction to Energy= 0.003374

Thermal correction to Enthalpy= 0.004318

Thermal correction to Gibbs Free Energy= -0.025389

Sum of electronic and zero-point Energies= -22.913424

Sum of electronic and thermal Energies= -22.910496

Sum of electronic and thermal Enthalpies= -22.909552

Sum of electronic and thermal Free Energies= -22.939259

Compound **CTC^{Me}*$2I_2$** in dichloromethane solution

Cartesian Coordinates

Au 0.380262 3.145327 4.594098

Au -0.806617 1.357367 1.644557

Au 2.328499 3.201509 1.527372

I -0.848385 5.082026 2.923366

N -2.276938 1.991087 5.577468

C -1.351985 2.097936 4.578472

N -1.795578 1.372237 3.520120

C -2.985730 0.776567 3.882902

C -3.293495 1.155093 5.165518

N 4.122929 4.749334 3.468997

C 2.969080 4.057313 3.240852

N 2.265316 4.076440 4.409563

C 2.975460 4.804385 5.348071

C 4.138567 5.228393 4.767775

N 0.040555 1.018406 -1.286926

C 0.294932 1.524012 -0.040781

N 1.459633 2.225432 -0.127393

C 1.920373 2.153189 -1.430607

C 1.040436 1.403661 -2.161691

H -3.527742 0.134962 3.202690

H -4.123247 0.905853 5.811585
H 2.604811 4.952620 6.352591
H 4.963907 5.810499 5.151888
H 2.837738 2.637591 -1.734841
H 1.031936 1.111834 -3.202351
I -1.337900 7.260988 0.933994
I 1.759973 8.194634 1.688824
I 4.380216 9.011245 2.510951
C 5.164251 4.995118 2.472424
H 6.146602 4.894466 2.944817
H 5.058137 4.254178 1.675733
H 5.057322 6.002920 2.056641
C -2.165277 2.602055 6.902112
H -1.731389 1.890458 7.613552
H -1.520579 3.481619 6.823991
H -3.160650 2.904267 7.240158
C -1.129486 0.227513 -1.661596
H -1.670706 -0.038286 -0.749660
H -1.784731 0.811052 -2.318897
H -0.808600 -0.683399 -2.178639

HF=-1248.7201185

Zero-point vibrational energy 694659.9 (Joules/Mol)

Zero-point correction= 0.264582 (Hartree/Particle)

Thermal correction to Energy= 0.297625

Thermal correction to Enthalpy= 0.298569

Thermal correction to Gibbs Free Energy= 0.186018

Sum of electronic and zero-point Energies= -1248.455537

Sum of electronic and thermal Energies= -1248.422494

Sum of electronic and thermal Enthalpies= -1248.421550

Sum of electronic and thermal Free Energies= -1248.534101

Compound **CTC^{Bz}*$2I_2$** in dichloromethane solution

Cartesian Coordinates

Au 0.636646 3.210322 4.426554
Au -0.741821 1.340623 1.621606
Au 2.610162 2.757836 1.406682
I -0.388855 5.089044 2.560439
N -2.084725 2.322952 5.528919
C -1.181544 2.315486 4.500376
N -1.719281 1.592159 3.485504
C -2.949387 1.122427 3.896704
C -3.185200 1.570922 5.170549
C -1.869592 2.906922 6.869713
C -1.272655 4.293446 6.809144
C -1.938536 5.324137 6.133276
C -1.349668 6.583436 6.016459
C -0.090855 6.828194 6.580558
C 0.572588 5.808070 7.267466
C -0.015582 4.542505 7.378817
N 4.546769 4.269525 3.252262
C 3.316759 3.709402 3.044376
N 2.604575 3.914369 4.189243
C 3.384993 4.615426 5.091560

C 4.601940 4.840561 4.513770
C 5.626533 4.367128 2.250236
C 5.829782 3.081851 1.479334
C 5.737641 3.082732 0.081815
C 5.843251 1.886828 -0.636344
C 6.033962 0.679345 0.042212
C 6.136842 0.674816 1.439964
C 6.040344 1.871007 2.153640
N -0.034082 0.919962 -1.314744
C 0.368655 1.297494 -0.062792
N 1.634007 1.779335 -0.181275
C 2.010286 1.703741 -1.512670
C 0.971457 1.178164 -2.230145
C -1.399846 0.523275 -1.642183
C -2.324650 1.698910 -1.922935
C -2.136373 2.958871 -1.336190
C -3.050047 3.991917 -1.572465
C -4.160476 3.779334 -2.397144
C -4.350749 2.525106 -2.989681
C -3.435206 1.493826 -2.754924
H -3.564976 0.510065 3.253181
H -4.014729 1.420414 5.846624
H -1.215251 2.236803 7.438559
H -2.851273 2.921823 7.355321
H -2.903174 5.127095 5.669340
H -1.859801 7.372973 5.470573
H 0.365193 7.810893 6.478057
H 1.549194 5.992201 7.710346
H 0.507744 3.736578 7.890192
H 3.012923 4.887931 6.068300
H 5.490259 5.339526 4.874004
H 6.533207 4.640832 2.802084
H 5.388842 5.183925 1.558858
H 5.549448 4.019487 -0.439568
H 5.758377 1.896910 -1.721148
H 6.099223 -0.254581 -0.512451
H 6.284965 -0.262933 1.971997
H 6.089619 1.863873 3.240943
H 2.990404 2.022566 -1.837910
H 0.855998 0.956524 -3.281530
H -1.777092 -0.048161 -0.784283
H -1.366537 -0.147698 -2.507090
H -1.281600 3.137951 -0.690007
H -2.888644 4.964236 -1.110639
H -4.869058 4.584785 -2.580510
H -5.206627 2.351129 -3.639307
H -3.582927 0.520355 -3.221438
I -1.201028 7.227467 0.597739
I -0.701511 9.351744 3.056545
I -0.220933 10.979180 5.378231

HF=-1941.5787287

Zero-point vibrational energy 1319176.1 (Joules/Mol)

Zero-point correction= 0.502448 (Hartree/Particle)

Thermal correction to Energy= 0.548497

Thermal correction to Enthalpy= 0.549442

Thermal correction to Gibbs Free Energy= 0.405533

Sum of electronic and zero-point Energies= -1941.076281

Sum of electronic and thermal Energies= -1941.030231

Sum of electronic and thermal Enthalpies= -1941.029287

Sum of electronic and thermal Free Energies= -1941.173195

Compound **I_3^-** in dichloromethane solution

Cartesian Coordinates

I 0.292200 5.559900 2.404018

I 0.292200 5.559900 5.469950

I 0.292200 5.559900 8.537532

HF=-34.5770036

Zero-point vibrational energy 1715.2 (Joules/Mol)

Zero-point correction= 0.000653 (Hartree/Particle)

Thermal correction to Energy= 0.006179

Thermal correction to Enthalpy= 0.007123

Thermal correction to Gibbs Free Energy= -0.032343

Sum of electronic and zero-point Energies= -34.576350

Sum of electronic and thermal Energies= -34.570824

Sum of electronic and thermal Enthalpies= -34.569880

Sum of electronic and thermal Free Energies= -34.609347

Compound **$CTC^{Me}I^+$** in dichloromethane solution

Cartesian Coordinates

Au 1.052164 3.498161 3.892975

Au -1.117762 2.168457 1.315735

Au 2.409885 2.726866 0.641648

I 0.368542 5.792604 2.639990

N -1.634952 3.408191 5.335000

C -0.902215 3.107197 4.222872

N -1.738081 2.565780 3.293792

C -2.972141 2.431983 3.874773

C -2.918044 2.955697 5.150911

C -1.099379 3.999044 6.565594

N 4.909243 3.581490 2.197604

C 3.569189 3.331609 2.170178

N 3.100499 3.598996 3.436456

C 4.138775 4.057018 4.211989

C 5.278022 4.040185 3.448062

C 5.827305 3.429534 1.066570

N -0.897309 1.411243 -1.641968

C -0.298560 1.853919 -0.494332

N 1.022520 2.066438 -0.791309

C 1.226988 1.756778 -2.116595

C 0.032703 1.348072 -2.657878

C -2.318853 1.097807 -1.793600

H -3.803316 1.984897 3.348597
H -3.663900 3.020814 5.930492
H -0.300384 4.695402 6.297693
H -0.704518 3.213975 7.218668
H 4.004920 4.323707 5.250750
H 6.303187 4.284730 3.687945
H 6.258870 4.402402 0.808578
H 5.261823 3.039529 0.217091
H 2.200602 1.842723 -2.577969
H -0.229662 1.022987 -3.654671
H -2.764247 1.030092 -0.797957
H -2.426217 0.140125 -2.312474
H -1.903736 4.538313 7.071539
H 6.625003 2.728583 1.333441
H -2.817764 1.887254 -2.366795

HF=-1214.1030962

Zero-point vibrational energy 691479.2 (Joules/Mol)

Zero-point correction= 0.263371 (Hartree/Particle)

Thermal correction to Energy= 0.288924

Thermal correction to Enthalpy= 0.289868

Thermal correction to Gibbs Free Energy= 0.201614

Sum of electronic and zero-point Energies= -1213.839726

Sum of electronic and thermal Energies= -1213.814172

Sum of electronic and thermal Enthalpies= -1213.813228

Sum of electronic and thermal Free Energies= -1213.901483

Compound **CTC**^{Bz}**I**$^{+}$ in dichloromethane solution

Cartesian Coordinates

Au 0.247718 3.509765 4.012141
Au -0.832506 1.598641 1.119797
Au 2.482189 3.057748 1.187985
I -0.667113 5.410531 2.277560
N -2.520995 2.503233 4.905828
C -1.530605 2.534242 3.957364
N -1.977038 1.855601 2.868106
C -3.211577 1.334716 3.166184
C -3.559569 1.732011 4.436123
C -2.412566 2.971585 6.303365
C -1.648654 4.265439 6.425815
C -2.130604 5.437969 5.827988
C -1.394905 6.619158 5.911991
C -0.182294 6.647266 6.618220
C 0.294713 5.487663 7.231876
C -0.429300 4.294079 7.127247
N 4.206377 4.674144 3.133528
C 3.023589 4.049343 2.852170
N 2.227461 4.224384 3.956368
C 2.894140 4.997650 4.877671
C 4.137391 5.278155 4.374099
C 5.344565 4.827943 2.200940

C 5.653424 3.560070 1.439344
C 5.650450 3.569357 0.038415
C 5.870364 2.387952 -0.677445
C 6.081280 1.186346 0.005546
C 6.088751 1.173234 1.406899
C 5.882086 2.355613 2.119891
N 0.253900 0.869186 -1.652522
C 0.450776 1.447099 -0.426236
N 1.715703 1.967063 -0.434725
C 2.294382 1.701753 -1.656664
C 1.388749 1.015401 -2.425079
C -0.955591 0.133599 -2.074282
C -2.234208 0.831334 -1.664909
C -3.185272 0.150991 -0.893333
C -4.334822 0.809362 -0.443141
C -4.533282 2.158452 -0.751986
C -3.588390 2.840815 -1.530032
C -2.448524 2.178651 -1.989502
H -3.754004 0.728320 2.455282
H -4.425965 1.515362 5.044923
H -1.928123 2.183644 6.890287
H -3.439219 3.090641 6.665838
H -3.064013 5.412379 5.270175
H -1.762975 7.521238 5.429494
H 0.384813 7.572869 6.686352
H 1.227900 5.506136 7.790363
H -0.057365 3.382394 7.589966
H 2.454559 5.254903 5.828651
H 4.973535 5.820865 4.791820
H 6.198716 5.136868 2.814200
H 5.113006 5.640624 1.503413
H 5.449094 4.499777 -0.489019
H 5.862983 2.404735 -1.765400
H 6.236723 0.263201 -0.548998
H 6.252433 0.239914 1.941808
H 5.864518 2.341405 3.208003
H 3.301255 2.019660 -1.886305
H 1.446007 0.628505 -3.432587
H -0.922311 -0.872929 -1.641904
H -0.891486 0.038124 -3.164186
H -3.008175 -0.888280 -0.622690
H -5.065474 0.272864 0.159030
H -5.417692 2.677710 -0.388513
H -3.739146 3.890710 -1.772940
H -1.701082 2.716719 -2.569277

HF=-1906.9671695

Zero-point vibrational energy 1314823.5 (Joules/Mol)

Zero-point correction= 0.500790 (Hartree/Particle)

Thermal correction to Energy= 0.539484

Thermal correction to Enthalpy= 0.540428

Thermal correction to Gibbs Free Energy= 0.421706

Sum of electronic and zero-point Energies= -1906.466380

Sum of electronic and thermal Energies= -1906.427686

Sum of electronic and thermal Enthalpies= -1906.426742

Sum of electronic and thermal Free Energies= -1906.545463

Compound **CTC^{Me}I$_2$** in dichloromethane solution

Cartesian Coordinates

Au -0.414276 3.255373 4.549721
Au -1.560087 1.874382 1.412014
Au 1.981272 2.574521 1.936879
I -0.901529 5.856406 3.932661
N -3.414215 3.034016 5.039862
C -2.378075 2.840400 4.188651
N -2.841195 2.349826 3.028140
C -4.218538 2.236849 3.148751
C -4.584402 2.663047 4.396587
C -3.304763 3.627664 6.372071
N 3.763307 3.775133 4.144858
C 2.529874 3.378700 3.706616
N 1.664443 3.642991 4.718068
C 2.348235 4.201215 5.779075
C 3.667318 4.286030 5.427675
C 5.003860 3.684337 3.377440
N -0.313956 0.993103 -1.248543
C -0.153748 1.509428 0.008894
N 1.176122 1.755507 0.170002
C 1.834621 1.393326 -0.992498
C 0.912611 0.917993 -1.883868
C -1.588137 0.619199 -1.858331
H -4.823413 1.854966 2.339036
H -5.544743 2.730780 4.887070
H -4.198426 3.371425 6.946194
H -3.208625 4.715743 6.286267
H 1.846448 4.494695 6.690117
H 4.530994 4.654768 5.962557
H 5.730557 3.066215 3.916305
H 5.418787 4.686166 3.219054
H 2.905946 1.497936 -1.092171
H 1.011564 0.536925 -2.890420
H -2.352835 0.604891 -1.077184
H -1.501989 -0.375869 -2.307656
I -0.000677 0.615942 5.039185
H 4.775398 3.225337 2.412157
H -1.867042 1.347331 -2.629190
H -2.416985 3.223605 6.867257

HF=-1225.8087469

Zero-point vibrational energy 694360.1 (Joules/Mol)

Zero-point correction= 0.264468 (Hartree/Particle)

Thermal correction to Energy= 0.292264

Thermal correction to Enthalpy= 0.293208

Thermal correction to Gibbs Free Energy= 0.198773

Sum of electronic and zero-point Energies= -1225.544279

Sum of electronic and thermal Energies= -1225.516483

Sum of electronic and thermal Enthalpies= -1225.515539

Sum of electronic and thermal Free Energies= -1225.609974

Compound **CTC^{Bz}I$_2$** in dichloromethane solution

Cartesian Coordinates

Au 0.617887 3.327555 4.433440
Au -0.829147 1.615095 1.591386
Au 2.514829 3.045505 1.364985
I -0.437540 5.362229 2.964597
I 1.663434 1.129880 5.661557
N -2.039814 2.340732 5.587849
C -1.219454 2.438428 4.508795
N -1.793965 1.817544 3.465264
C -3.011700 1.313835 3.896941
C -3.172232 1.632625 5.216685
C -1.713403 2.769342 6.960806
C -1.212243 4.194468 7.023984
C -2.006227 5.251702 6.561024
C -1.517350 6.558774 6.580065
C -0.230804 6.821969 7.067676
C 0.563670 5.771641 7.536331
C 0.073669 4.461286 7.513953
N 4.509946 4.437763 3.244865
C 3.254902 3.941082 3.017882
N 2.565829 4.120416 4.171326
C 3.377771 4.721901 5.112808
C 4.599961 4.928961 4.536504
C 5.594978 4.485035 2.248042
C 5.758609 3.173101 1.510519
C 5.721295 3.149279 0.110889
C 5.784610 1.934031 -0.579556
C 5.874844 0.732185 0.129123
C 5.921692 0.751685 1.529633
C 5.869640 1.966441 2.215712
N -0.066431 1.041069 -1.311013
C 0.285647 1.545583 -0.088738
N 1.531950 2.071515 -0.220915
C 1.945713 1.899205 -1.531525
C 0.950661 1.264422 -2.222523
C -1.382774 0.491058 -1.630281
C -2.357544 1.532094 -2.153392
C -2.430677 2.813687 -1.586900
C -3.372809 3.737092 -2.050397
C -4.251562 3.391013 -3.084941
C -4.180645 2.115255 -3.655650
C -3.235233 1.192742 -3.192214
H -3.666532 0.776631 3.226088
H -3.968953 1.435287 5.919349
H -0.953938 2.084781 7.355365
H -2.630903 2.647172 7.545829
H -2.996231 5.044584 6.159067
H -2.134368 7.372908 6.205871
H 0.151368 7.840729 7.074778
H 1.564594 5.969761 7.914290
H 0.697223 3.635436 7.849005
H 3.022609 4.947568 6.107404
H 5.509187 5.373404 4.915436
H 6.507138 4.745821 2.797211
H 5.384717 5.290035 1.534506
H 5.607347 4.083788 -0.435552
H 5.742379 1.925801 -1.666892
H 5.904276 -0.215783 -0.404429
H 5.989855 -0.181408 2.085535
H 5.870928 1.978978 3.303951
H 2.919116 2.232533 -1.862210

H 0.872523 0.947654 -3.252779
H -1.767036 0.044175 -0.704675
H -1.251617 -0.309900 -2.365546
H -1.750403 3.087113 -0.784005
H -3.419670 4.728590 -1.603557
H -4.982836 4.111813 -3.445700
H -4.854250 1.839785 -4.465015
H -3.176205 0.201923 -3.641224

HF=-1918.6679655

Zero-point vibrational energy 1318513.6 (Joules/Mol)

Zero-point correction= 0.502195 (Hartree/Particle)

Thermal correction to Energy= 0.542994

Thermal correction to Enthalpy= 0.543939

Thermal correction to Gibbs Free Energy= 0.417562

Sum of electronic and zero-point Energies= -1918.165770

Sum of electronic and thermal Energies= -1918.124971

Sum of electronic and thermal Enthalpies= -1918.124027

Sum of electronic and thermal Free Energies= -1918.250404

Compound **$CTC^{Me}I_2*2I_2$**

Cartesian Coordinates

Au -0.455140 3.515768 4.368687
Au -1.620602 1.796115 1.388810
Au 1.910812 2.480591 1.812283
I -1.156883 6.121680 4.076836
N -3.429554 3.167558 4.966317
C -2.408490 3.006995 4.089958
N -2.879029 2.474431 2.950128
C -4.247053 2.305318 3.109541
C -4.598054 2.735998 4.359226
C -3.327100 3.765683 6.297416
N 3.647953 4.261246 3.610234
C 2.463075 3.620507 3.400063
N 1.619402 4.003867 4.387874
C 2.286833 4.860966 5.235215
C 3.560356 5.023825 4.757069
C 4.839589 4.132378 2.771190
N -0.381776 0.433230 -1.069406
C -0.223148 1.143658 0.084966
N 1.119228 1.329273 0.245158
C 1.784480 0.745647 -0.818329
C 0.853412 0.176692 -1.641070
C -1.658706 -0.034716 -1.605907
H -4.854209 1.879493 2.323868
H -5.547539 2.770580 4.873689
H -4.097540 3.329220 6.938487
H -3.458222 4.851229 6.232679
H 1.804886 5.299257 6.096760
H 4.392674 5.609892 5.118106
H 5.514520 3.373843 3.183084
H 5.348756 5.099429 2.729907

H 2.861437 0.780011 -0.902882
H 0.951488 -0.379949 -2.562154
H -2.463060 0.478416 -1.072619
H -1.754849 -1.116992 -1.460975
I 0.113745 0.887061 4.850513
H 4.526134 3.839311 1.765755
H -1.713885 0.200153 -2.673888
H -2.337916 3.543913 6.706194
I 0.931576 4.873071 0.649112
I 0.034491 7.611067 -0.152251
I 2.082590 8.547291 2.309289
I 3.876318 9.110197 4.465969

HF=-1271.6848733
Zero-point vibrational energy 700282.5 (Joules/Mol)
Zero-point correction= 0.266724 (Hartree/Particle)
Thermal correction to Energy= 0.304049
Thermal correction to Enthalpy= 0.304994
Thermal correction to Gibbs Free Energy= 0.181700
Sum of electronic and zero-point Energies= -1271.418150
Sum of electronic and thermal Energies= -1271.380824
Sum of electronic and thermal Enthalpies= -1271.379880
Sum of electronic and thermal Free Energies= -1271.503173

Compound **$CTC^{Me}I_3^+$**

Cartesian Coordinates

Au -0.485352 3.463850 4.497157
Au -1.697534 2.285443 1.265618
Au 1.875002 2.473650 2.057303
I -1.263328 6.000277 4.836643
N -3.435154 2.923942 5.067848
C -2.435424 2.973455 4.152270
N -2.936962 2.743320 2.926430
C -4.296674 2.521857 3.078200
C -4.614727 2.637152 4.404879
C -3.302622 3.187551 6.502618
N 3.721776 3.853592 4.129598
C 2.480005 3.499977 3.725323
N 1.588852 3.966075 4.621097
C 2.281142 4.636280 5.611550
C 3.613655 4.568937 5.311568
C 4.979572 3.551742 3.441074
N -0.378133 1.063577 -1.223983
C -0.261780 1.621742 0.014665
N 1.051986 1.520259 0.357341
C 1.750318 0.916580 -0.673028
C 0.857078 0.617716 -1.662227
C -1.634215 0.892205 -1.954072
H -4.922436 2.279347 2.231617
H -5.548717 2.527273 4.936849

H -4.092085 2.647334 7.030713
H -3.386792 4.262501 6.692659
H 1.771482 5.094264 6.446442
H 4.486534 4.947314 5.823473
H 5.736295 3.304187 4.190457
H 5.299806 4.416235 2.851342
H 2.817831 0.758863 -0.622139
H 0.983877 0.136826 -2.621545
H -2.384299 1.547395 -1.503890
H -1.968952 -0.149279 -1.888019
I 0.505474 0.856416 3.936502
H 4.821187 2.696591 2.781328
H -1.482595 1.165803 -3.002951
H -2.325130 2.832578 6.839263
I 2.853138 4.410474 0.482328

HF=-1237.0734475

Zero-point vibrational energy 698765.0 (Joules/Mol)

Zero-point correction= 0.266146 (Hartree/Particle)

Thermal correction to Energy= 0.295717

Thermal correction to Enthalpy= 0.296661

Thermal correction to Gibbs Free Energy= 0.199060

Sum of electronic and zero-point Energies= -1236.807302

Sum of electronic and thermal Energies= -1236.777731

Sum of electronic and thermal Enthalpies= -1236.776787

Sum of electronic and thermal Free Energies= -1236.874387

Compound **$CTC^{Me}I_4$**

Cartesian Coordinates

Au -0.420151 3.281441 4.578650
Au -1.539328 2.040847 1.357840
Au 2.016523 2.501656 1.954959
I -0.883721 5.964994 4.753852
N -3.442068 3.111959 4.994085
C -2.388523 2.981887 4.152260
N -2.828951 2.617277 2.937158
C -4.212176 2.535635 3.010473
C -4.601722 2.842818 4.285035
C -3.369829 3.522921 6.395949
N 3.788964 3.734696 4.125563
C 2.556671 3.319766 3.744512
N 1.693357 3.568829 4.743989
C 2.396482 4.148664 5.784918
C 3.705442 4.258047 5.405803
C 4.980933 3.729515 3.276548
N -0.292424 0.924295 -1.217772
C -0.134365 1.500849 0.011189
N 1.203508 1.648980 0.193130
C 1.875246 1.176901 -0.916404
C 0.943181 0.723738 -1.808295
C -1.575888 0.619518 -1.848108

H -4.803027 2.252833 2.151350
H -5.574002 2.891252 4.753793
H -4.213274 3.082442 6.933956
H -3.400499 4.615007 6.469239
H 1.905416 4.439666 6.701684
H 4.575319 4.644955 5.916427
H 5.867065 3.731771 3.916135
H 4.980928 4.614759 2.631095
H 2.953850 1.189287 -0.976186
H 1.042978 0.279804 -2.788485
H -2.353581 0.660283 -1.081087
H -1.538729 -0.384178 -2.283885
I -0.175486 0.584957 4.801329
H 4.970142 2.824826 2.663100
H -1.795602 1.354106 -2.631626
H -2.430585 3.157242 6.819173
I 1.140096 4.959143 1.181104
I 3.313329 0.175914 2.548146

HF=-1248.7700773

Zero-point vibrational energy 699663.4 (Joules/Mol)

Zero-point correction= 0.266488 (Hartree/Particle)

Thermal correction to Energy= 0.298570

Thermal correction to Enthalpy= 0.299514

Thermal correction to Gibbs Free Energy= 0.194821

Sum of electronic and zero-point Energies= -1248.503590

Sum of electronic and thermal Energies= -1248.471508

Sum of electronic and thermal Enthalpies= -1248.470563

Sum of electronic and thermal Free Energies= -1248.575256

$CTC^{Me}I_4*2I_2$

Cartesian Coordinates

Au -0.250304 3.154259 4.831731
Au -1.770844 2.553964 1.608567
Au 1.851101 2.600136 1.881689
I -0.352024 5.794903 5.477338
N -3.232651 3.277603 5.514629
C -2.269638 3.156168 4.572187
N -2.850126 3.025429 3.366822
C -4.222430 3.093568 3.550964
C -4.468277 3.249453 4.886220
C -3.008432 3.461803 6.949533
N 3.921639 3.267363 4.029968
C 2.624313 3.038918 3.714449
N 1.882094 3.173476 4.826570
C 2.730723 3.488296 5.873660
C 4.005052 3.550737 5.383649
C 5.028661 3.318206 3.073438

N -0.908971 1.663267 -1.203266
C -0.565651 2.059096 0.055765
N 0.785078 2.125517 0.102456
C 1.291864 1.758035 -1.124687
C 0.235838 1.474319 -1.949846
C -2.270321 1.483912 -1.710467
H -4.904657 3.016008 2.717156
H -5.387713 3.336243 5.446688
H -3.859287 3.041258 7.491482
H -2.900598 4.527075 7.177458
H 2.354715 3.648219 6.873214
H 4.951718 3.760047 5.860110
H 5.964714 3.153142 3.612429
H 5.046595 4.295943 2.579681
H 2.355128 1.710317 -1.308200
H 0.194419 1.160933 -2.982870
H -2.955372 1.442697 -0.861263
H -2.320420 0.546268 -2.271983
I -0.356391 0.459009 4.447693
H 4.887267 2.530650 2.328963
H -2.537480 2.325295 -2.358278
H -2.093740 2.935116 7.232958
I 1.194921 5.214987 1.600002
I 2.941303 0.099100 1.954581
I -2.805192 4.735605 0.129981
I -3.824261 6.864052 -1.739529
I -2.155304 5.045875 -3.985332
I -0.675858 3.274532 -5.676011

HF=-1294.6411914

Zero-point vibrational energy 705370.3 (Joules/Mol)

Zero-point correction= 0.268661 (Hartree/Particle)

Thermal correction to Energy= 0.310390

Thermal correction to Enthalpy= 0.311334

Thermal correction to Gibbs Free Energy= 0.176146

Sum of electronic and zero-point Energies= -1294.372530

Sum of electronic and thermal Energies= -1294.330802

Sum of electronic and thermal Enthalpies= -1294.329858

Sum of electronic and thermal Free Energies= -1294.465046

Compound **$CTC^{Me}I_5^+$**

Cartesian Coordinates

Au -0.375024 3.161765 4.560471
Au -1.502570 2.145661 1.351219
Au 2.078265 2.789426 1.824502
I -0.580981 5.711998 5.388210
N -3.441555 3.315425 4.934030
C -2.374119 3.122756 4.127291
N -2.813758 2.877337 2.877879
C -4.195032 2.928650 2.892408
C -4.593321 3.201698 4.171417
C -3.408471 3.585557 6.374235

N 3.801764 3.764953 4.148393
C 2.591088 3.364706 3.705252
N 1.749284 3.276770 4.752498
C 2.458708 3.626350 5.892875
C 3.739165 3.923575 5.524000
C 4.993317 3.939920 3.314760
N -0.261076 0.782530 -1.090136
C -0.079992 1.551791 0.010517
N 1.203407 1.949587 0.056180
C 1.853484 1.409309 -1.037635
C 0.945176 0.687055 -1.761100
C -1.525948 0.186676 -1.528710
H -4.774348 2.758918 1.996882
H -5.573667 3.319600 4.609874
H -4.204046 3.009337 6.854341
H -3.548897 4.654854 6.557585
H 1.986120 3.657020 6.863122
H 4.599696 4.236696 6.096990
H 5.644439 4.677219 3.790687
H 4.680180 4.302226 2.332026
H 2.912058 1.557132 -1.190520
H 1.042585 0.107759 -2.667659
H -2.145714 -0.009448 -0.650650
H -1.307408 -0.753014 -2.042376
I -0.463587 0.560745 3.455794
H 5.516874 2.984571 3.205126
H -2.048685 0.874285 -2.200868
H -2.438561 3.272051 6.764536
I 0.982611 5.227918 1.392150
I 3.506407 0.494774 2.225131
I -2.590370 3.696162 -0.540027

HF=-1260.0336785

Zero-point vibrational energy 704020.9 (Joules/Mol)

Zero-point correction= 0.268147 (Hartree/Particle)

Thermal correction to Energy= 0.302055

Thermal correction to Enthalpy= 0.302999

Thermal correction to Gibbs Free Energy= 0.194536

Sum of electronic and zero-point Energies= -1259.765531

Sum of electronic and thermal Energies= -1259.731623

Sum of electronic and thermal Enthalpies= -1259.730679

Sum of electronic and thermal Free Energies= -1259.839143

Compound **$CTC^{Me}I_6$**

Cartesian Coordinates

Au -0.425866 3.294618 4.559554
Au -1.539209 1.905580 1.376858
Au 2.009966 2.553483 1.950999
I -0.858005 5.893967 3.898365
N -3.434824 3.105149 4.983537
C -2.378628 2.897494 4.163642

N -2.823636 2.394142 3.003369
C -4.200557 2.282788 3.085023
C -4.589912 2.726925 4.318791
C -3.346690 3.697760 6.318629
N 3.791122 3.637728 4.172134
C 2.543200 3.324357 3.753403
N 1.672356 3.619118 4.729379
C 2.384248 4.138666 5.796104
C 3.708600 4.155668 5.454393
C 5.001770 3.488153 3.363569
N -0.311113 1.049650 -1.276861
C -0.119161 1.534768 -0.027988
N 1.190626 1.759735 0.151353
C 1.848753 1.414398 -1.015973
C 0.916758 0.972226 -1.913718
C -1.614755 0.733639 -1.862105
H -4.782845 1.893757 2.262927
H -5.561613 2.804491 4.784378
H -4.284451 3.511534 6.846914
H -3.166825 4.774616 6.230624
H 1.889871 4.448532 6.704908
H 4.588672 4.476340 5.992516
H 5.872142 3.605455 4.012964
H 5.012858 4.247300 2.574222
H 2.921073 1.506112 -1.105531
H 1.006013 0.609139 -2.927214
H -2.226207 0.233277 -1.105835
H -1.462849 0.068905 -2.715675
I -0.240969 0.929356 5.882916
H 5.002570 2.490677 2.913575
H -2.110216 1.656221 -2.183149
H -2.515903 3.229958 6.855519
I 2.071881 5.060331 0.905871
I 2.663289 0.077615 2.854520
I -1.463356 -0.706494 2.109150
I -2.070178 4.268265 0.144239

HF=-1271.7231554

Zero-point vibrational energy 703321.8 (Joules/Mol)

Zero-point correction= 0.267881 (Hartree/Particle)

Thermal correction to Energy= 0.304722

Thermal correction to Enthalpy= 0.305667

Thermal correction to Gibbs Free Energy= 0.186179

Sum of electronic and zero-point Energies= -1271.455274

Sum of electronic and thermal Energies= -1271.418433

Sum of electronic and thermal Enthalpies= -1271.417489

Sum of electronic and thermal Free Energies= -1271.536976

Compound **CTC^{Bz}** in iodomethane solution

Cartesian Coordinates

N 5.041562 1.862889 8.673338
N 4.401782 -0.216831 8.938648
N 3.738126 -5.310451 8.767958
N 5.085346 -4.787984 7.120155

N 8.040909 -2.459496 3.625484
N 7.268974 -0.888294 4.945277
C 5.134064 0.605440 8.139094
C 4.268686 1.831641 9.821248
H 4.078145 2.721074 10.404832
C 3.869299 0.532877 9.974183
H 3.251869 0.078308 10.736032
C 5.781814 3.032612 8.191962
H 5.128981 3.908737 8.269264
H 5.994070 2.850482 7.132080
C 7.067097 3.260354 8.965090
C 8.046023 2.256240 9.028444
H 7.869864 1.305914 8.528027
C 9.233674 2.472154 9.732080
H 9.986957 1.687523 9.774275
C 9.455995 3.694498 10.381458
H 10.380795 3.861161 10.930565
C 8.483446 4.698133 10.322716
H 8.647218 5.648552 10.827282
C 7.292872 4.478859 9.617954
H 6.532886 5.258105 9.574949
C 4.341869 -4.258106 8.130627
C 4.096665 -6.500247 8.157621
H 3.725181 -7.453175 8.506886
C 4.937243 -6.164757 7.132861
H 5.445188 -6.795336 6.416613
C 2.788425 -5.197384 9.888612
H 2.644757 -6.213242 10.274966
H 1.825749 -4.842592 9.501994
C 3.277848 -4.269017 10.979863
C 4.554141 -4.431666 11.535895
H 5.186873 -5.248959 11.194970
C 5.031472 -3.524353 12.483722
H 6.029564 -3.649756 12.898973
C 4.231212 -2.449655 12.894013
H 4.606852 -1.737630 13.626161
C 2.951221 -2.292018 12.353958
H 2.326031 -1.456860 12.663929
C 2.479951 -3.196248 11.396209
H 1.500282 -3.053214 10.944017
C 7.272870 -2.233065 4.736269
C 8.507084 -1.258021 3.121282
H 9.128586 -1.212484 2.238311
C 8.027417 -0.285931 3.954765
H 8.172293 0.785021 3.926253
C 8.221729 -3.764475 2.986286
H 8.094493 -4.516884 3.773731
H 9.250471 -3.825204 2.615235
C 7.238669 -4.006199 1.855675
C 5.859843 -3.837075 2.056625
H 5.497372 -3.505921 3.027636
C 4.959619 -4.089457 1.018054
H 3.892242 -3.955598 1.184426
C 5.426961 -4.513769 -0.233360
H 4.724706 -4.708622 -1.041666
C 6.800278 -4.682530 -0.439525
H 7.171999 -5.006765 -1.409779
C 7.701016 -4.426693 0.601911
H 8.770962 -4.552637 0.440536
Au 6.211165 -0.002664 6.540904
Au 4.270140 -2.293157 8.605507
Au 6.252101 -3.582250 5.840439

HF=-1895.6935902

Zero-point vibrational energy 1313599.9 (Joules/Mol)

Zero-point correction= 0.500324 (Hartree/Particle)
Thermal correction to Energy= 0.536679
Thermal correction to Enthalpy= 0.537623
Thermal correction to Gibbs Free Energy= 0.423836
Sum of electronic and zero-point Energies= -1895.193266
Sum of electronic and thermal Energies= -1895.156911
Sum of electronic and thermal Enthalpies= -1895.155967
Sum of electronic and thermal Free Energies= -1895.269754

Compound **CH_3I** in iodomethane solution

Cartesian Coordinates

C -4.031762 1.708550 -0.036100
H -3.644824 0.689174 0.006458
H -3.645154 2.254498 -0.898057
H -5.121720 1.732925 0.006088
I -3.297140 2.746553 1.762511

HF=-51.353084

Zero-point vibrational energy 93398.3 (Joules/Mol)
Zero-point correction= 0.035574 (Hartree/Particle)
Thermal correction to Energy= 0.038783
Thermal correction to Enthalpy= 0.039727
Thermal correction to Gibbs Free Energy= 0.009761
Sum of electronic and zero-point Energies= -51.317510
Sum of electronic and thermal Energies= -51.314301
Sum of electronic and thermal Enthalpies= -51.313357
Sum of electronic and thermal Free Energies= -51.343323

Transition State $\mathbf{8^{Bz}}_{\mathbf{TS}}$

Imaginary Frequency at -424.5 cm^{-1}

Cartesian Coordinates

C 3.422185 10.523594 3.022435
C 3.257487 12.656449 3.748212
H 3.330046 13.731044 3.659389
C 2.885618 11.857672 4.774729
H 2.561200 12.103082 5.774840
C 0.026970 7.602002 4.961547

C -1.733615 6.360332 5.610640
H -2.605159 6.104573 6.196244
C -1.099312 5.718824 4.582889
H -1.327522 4.778353 4.102083
C 2.850578 5.987719 1.265462
C 4.314186 5.230617 -0.275008
H 4.760449 4.514806 -0.950674
C 4.626464 6.528179 0.019745
H 5.417934 7.160193 -0.357710
Au 3.666815 8.847160 1.936468
Au 1.391973 9.075242 4.774366
Au 1.385690 6.119411 2.654578
N 3.587300 11.816061 2.679046
N 2.983881 10.518816 4.352180
N -1.025661 7.529024 5.833016
N -0.018372 6.486952 4.185875
N 3.215552 4.909145 0.504659
N 3.725599 6.988419 0.965304
C 2.508913 3.616169 0.459475
H 1.592559 3.734726 -0.130650
H 3.169130 2.923286 -0.075199
C -1.313827 8.492979 6.909926
H -0.601271 8.330888 7.727002
H -2.316580 8.250457 7.280627
C 4.037415 12.306797 1.355006
H 3.177966 12.294500 0.675868
H 4.341426 13.348530 1.505881
I 6.647879 8.262860 6.709571
C 4.475776 9.607551 5.335224
H 4.387595 8.673575 4.797667
H 4.026806 9.695168 6.315098
H 5.236922 10.316132 5.036547
C -1.241499 9.931519 6.443865
C -0.420789 10.843790 7.118977
C -1.939411 10.350071 5.302361
C -0.291793 12.158077 6.657427
H 0.140394 10.512878 7.990961
C -1.803575 11.658229 4.833241
H -2.559408 9.637708 4.761490
C -0.975917 12.565496 5.508258
H 0.355605 12.855962 7.184825
H -2.337754 11.970213 3.937881
H -0.863547 13.582200 5.137019
C 5.168838 11.480625 0.786373
C 6.335736 11.263006 1.532448
C 5.020813 10.857447 -0.458579
C 7.332448 10.414748 1.047226
H 6.444697 11.727001 2.511029
C 6.023964 10.016678 -0.952367
H 4.102763 11.004072 -1.024278
C 7.177021 9.786724 -0.196041
H 8.227079 10.236427 1.640272
H 5.893690 9.526829 -1.915135
H 7.950240 9.117966 -0.568787
C 2.169819 3.086064 1.836089
C 0.845112 2.762683 2.154284
C 3.158810 2.981040 2.824091
C 0.507427 2.346641 3.446631
H 0.072736 2.868756 1.394622
C 2.823085 2.575773 4.117345
H 4.184675 3.256772 2.587260
C 1.494479 2.261102 4.433147
H -0.527144 2.108874 3.686245
H 3.594328 2.513756 4.882577
H 1.231782 1.955885 5.444085

HF=-1947.0282326

Zero-point vibrational energy 1409786.2 (Joules/Mol)

Zero-point correction= 0.536959 (Hartree/Particle)

Thermal correction to Energy= 0.577689

Thermal correction to Enthalpy= 0.578633

Thermal correction to Gibbs Free Energy= 0.453015

Sum of electronic and zero-point Energies= -1946.491273

Sum of electronic and thermal Energies= -1946.450543

Sum of electronic and thermal Enthalpies= -1946.449599

Sum of electronic and thermal Free Energies= -1946.579217

Compound **8**^{Bz}

Cartesian Coordinates

C 3.470880 10.334076 3.490580

C 3.302486 12.384017 4.429716

H 3.388942 13.459537 4.474054

C 2.823355 11.482865 5.329247

H 2.421916 11.612848 6.323388

C -0.419914 7.890076 4.302572

C -1.375714 6.674191 5.944505

H -1.988707 6.493914 6.816267

C -0.614290 5.842471 5.170216

H -0.440042 4.778488 5.251573

C 2.904244 5.961081 1.373347

C 4.372385 5.301486 -0.204435

H 4.830976 4.624559 -0.911299

C 4.652275 6.595732 0.137369

H 5.421987 7.263052 -0.223317

Au 3.681191 8.778347 2.225856

Au 0.052034 9.442119 3.058165

Au 1.411713 6.078643 2.730558

N 3.709502 11.664169 3.318098

N 2.942192 10.236071 4.744454

N -1.242715 7.938456 5.395557

N -0.031466 6.595430 4.164564

N 3.290072 4.921089 0.571988

N 3.748268 6.996337 1.105961

C 2.610915 3.615666 0.481951

H 1.706577 3.728035 -0.127753

H 3.297976 2.946463 -0.049052

C -1.865551 9.164005 5.913702

H -1.075957 9.827871 6.284558

H -2.486243 8.864306 6.766240

C 4.173107 12.339509 2.082309

H 3.282309 12.650028 1.525202

H 4.708547 13.235389 2.417116

C -2.688969 9.887678 4.868008

C -2.510131 11.261682 4.669439

C -3.603614 9.190822 4.066656

C -3.238057 11.937145 3.684505
H -1.776010 11.796341 5.269757
C -4.325560 9.860586 3.076431
H -3.725497 8.117522 4.200291
C -4.144807 11.236749 2.882886
H -3.080186 13.002426 3.529000
H -5.025213 9.309819 2.450535
H -4.700520 11.755865 2.104506
C 5.059488 11.484734 1.213465
C 6.316317 11.062898 1.669491
C 4.602108 11.057804 -0.039943
C 7.098886 10.210654 0.888017
H 6.666697 11.379834 2.650333
C 5.388239 10.210983 -0.827941
H 3.610839 11.358437 -0.372537
C 6.635480 9.782948 -0.363188
H 8.068704 9.878048 1.252709
H 5.018408 9.872337 -1.793543
H 7.244918 9.116095 -0.970160
C 2.251555 3.054552 1.840661
C 0.917748 2.760230 2.148278
C 3.234148 2.899845 2.828490
C 0.564209 2.326365 3.430771
H 0.151125 2.905691 1.389597
C 2.883777 2.474913 4.111480
H 4.268526 3.150003 2.599500
C 1.545695 2.191082 4.417279
H -0.477773 2.115086 3.662768
H 3.651082 2.371292 4.876319
H 1.271704 1.869856 5.420339
I 0.535590 11.335518 1.227329
C 2.599347 8.981745 5.421139
H 3.458724 8.625535 6.000562
H 1.748006 9.165285 6.080991
H 2.312865 8.240370 4.673022

HF=-1947.1089215

Zero-point vibrational energy 1418388.0 (Joules/Mol)

Zero-point correction= 0.540235 (Hartree/Particle)

Thermal correction to Energy= 0.580980

Thermal correction to Enthalpy= 0.581924

Thermal correction to Gibbs Free Energy= 0.458734

Sum of electronic and zero-point Energies= -1946.568686

Sum of electronic and thermal Energies= -1946.527941

Sum of electronic and thermal Enthalpies= -1946.526997

Sum of electronic and thermal Free Energies= -1946.650187

Transition State $\mathbf{9^{Bz}_{TS}}$

Imaginary Frequency at -426.8 cm^{-1}

Cartesian Coordinates

Au 0.482863 3.116169 4.493269

Au -0.023174 0.816463 1.801748
Au 3.372607 1.273403 3.356501
N -2.366743 2.100473 5.073701
C -1.241608 2.092537 4.293012
N -1.453036 1.188778 3.298464
C -2.709780 0.634810 3.462450
C -3.285499 1.194505 4.568219
C -2.559790 2.785091 6.373082
C -1.793507 4.079305 6.502037
C -2.061992 5.154813 5.643011
C -1.280396 6.310415 5.695469
C -0.233083 6.408625 6.621258
C 0.023132 5.349699 7.497668
C -0.753813 4.187972 7.435381
N 4.137946 4.255513 3.119204
C 3.378280 3.287140 3.670266
N 2.502046 3.929881 4.552235
C 2.754105 5.313365 4.481603
C 3.763518 5.514461 3.604250
C 5.216257 4.060624 2.121670
C 6.050550 2.830181 2.384734
C 6.707197 2.665418 3.612241
C 7.415871 1.492443 3.876615
C 7.485248 0.479734 2.910415
C 6.841618 0.645616 1.680999
C 6.122556 1.816327 1.422569
N 1.899083 -0.362072 -0.310218
C 1.423046 0.691827 0.407222
N 2.176297 1.754649 0.007608
C 3.117259 1.370421 -0.929431
C 2.948448 0.033684 -1.124227
C 1.476322 -1.778501 -0.192799
C 0.018534 -1.945830 0.154846
C -0.343376 -2.502148 1.388468
C -1.690759 -2.602764 1.749899
C -2.683545 -2.142052 0.879894
C -2.326505 -1.588865 -0.357114
C -0.981242 -1.497163 -0.719703
H -3.088994 -0.109392 2.776904
H -4.245516 1.038899 5.039732
H -2.259573 2.093462 7.169112
H -3.637645 2.963488 6.464639
H -2.858358 5.066674 4.906317
H -1.483897 7.133645 5.013596
H 0.376914 7.309148 6.660435
H 0.833152 5.420519 8.220869
H -0.530889 3.347360 8.089742
H 2.189844 6.009257 5.085004
H 4.265318 6.413344 3.275286
H 5.828847 4.968076 2.160475
H 4.760209 4.003041 1.128111
H 6.635125 3.441985 4.371579
H 7.910120 1.363836 4.837332
H 8.030734 -0.437543 3.121842
H 6.878954 -0.142028 0.931521
H 5.580391 1.927489 0.486543
H 3.801648 2.074737 -1.378348
H 3.468805 -0.664446 -1.762869
H 2.103930 -2.248473 0.571426
H 1.699960 -2.236499 -1.162994
H 0.437072 -2.816875 2.077792
H -1.961815 -3.022594 2.716350
H -3.732221 -2.210031 1.163156
H -3.097194 -1.229540 -1.036119
H -0.701410 -1.051461 -1.672794
C 1.947341 3.128373 0.462278
H 1.858352 3.129841 1.551365
H 2.792869 3.744804 0.149537

H 1.023570 3.514429 0.019660
I 3.260114 -1.378856 3.240159
C 3.008051 3.272124 6.365755
H 4.025378 3.641197 6.337917
H 2.251793 3.843236 6.885692
H 2.825735 2.227625 6.151330
I 3.651356 2.330799 9.034552

HF=-1998.4427635

Zero-point vibrational energy 1516864.1 (Joules/Mol)

Zero-point correction= 0.577743 (Hartree/Particle)

Thermal correction to Energy= 0.622627

Thermal correction to Enthalpy= 0.623571

Thermal correction to Gibbs Free Energy= 0.489122

Sum of electronic and zero-point Energies= -1997.865020

Sum of electronic and thermal Energies= -1997.820137

Sum of electronic and thermal Enthalpies= -1997.819193

Sum of electronic and thermal Free Energies= -1997.955642

Compound **9**$^{\mathbf{Bz}}$

Cartesian Coordinates

Au 0.542193 3.687912 3.996405
Au -0.019695 0.909337 1.663715
N -1.358897 1.853043 5.523992
C -0.632325 2.068695 4.386481
N -0.885361 1.022702 3.557253
C -1.779574 0.165148 4.178098
C -2.084024 0.679927 5.408072
C -1.422868 2.803270 6.644955
C -2.191804 4.055670 6.276888
C -3.558394 3.987643 5.969932
C -4.254075 5.137648 5.585556
C -3.587828 6.368213 5.507039
C -2.225250 6.441988 5.815368
C -1.531864 5.288406 6.197558
N 0.871821 0.071997 -1.144806
C 0.872528 0.992691 -0.139432
N 1.581308 2.046408 -0.631497
C 2.010923 1.791261 -1.923039
C 1.568001 0.544327 -2.245403
C 0.293013 -1.284960 -1.048610
C -1.095881 -1.291328 -0.449389
C -1.371273 -2.101377 0.659247
C -2.633696 -2.071636 1.261553
C -3.624018 -1.217918 0.766321
C -3.353009 -0.407463 -0.344020
C -2.097363 -0.449531 -0.952969
H -2.124396 -0.735933 3.691676
H -2.727859 0.326679 6.200897
H -0.395158 3.061852 6.919699
H -1.891169 2.278454 7.484472
H -4.075843 3.030781 6.022391

H -5.314197 5.075501 5.346857
H -4.128908 7.262764 5.204568
H -1.698773 7.391991 5.748559
H -0.465579 5.335742 6.408657
H 2.582251 2.511963 -2.489263
H 1.686438 -0.045093 -3.142636
H 0.965175 -1.904909 -0.445272
H 0.284559 -1.683306 -2.069469
H -0.585609 -2.735134 1.066247
H -2.836012 -2.701506 2.125536
H -4.600419 -1.178553 1.244835
H -4.120035 0.260174 -0.731246
H -1.880415 0.198263 -1.800180
C 1.796421 3.318310 0.071521
H 1.057722 4.055407 -0.261136
H 1.685860 3.160114 1.147194
H 2.805574 3.677428 -0.149113
I 2.084882 5.797038 3.412342

HF=-1315.1836076
Zero-point vibrational energy 978633.3 (Joules/Mol)
Zero-point correction= 0.372742 (Hartree/Particle)
Thermal correction to Energy= 0.401136
Thermal correction to Enthalpy= 0.402080
Thermal correction to Gibbs Free Energy= 0.304368
Sum of electronic and zero-point Energies= -1314.810866
Sum of electronic and thermal Energies= -1314.782472
Sum of electronic and thermal Enthalpies= -1314.781528
Sum of electronic and thermal Free Energies= -1314.885239

Compound **4**
Cartesian Coordinates
N 3.579676 -5.440281 8.663932
N 5.070938 -4.848672 7.222370
C 4.346422 -4.384668 8.276114
C 3.825668 -6.548842 7.872764
H 3.318374 -7.489217 8.030089
C 4.762992 -6.173913 6.957546
H 5.231380 -6.721427 6.152897
C 2.682222 -5.407531 9.836471
H 2.066754 -6.311469 9.784115
H 2.034455 -4.532181 9.730915
C 3.458174 -5.334341 11.134372
C 4.304240 -6.385123 11.516821
H 4.381774 -7.272474 10.890196
C 5.056983 -6.291961 12.690608
H 5.712722 -7.110757 12.980346
C 4.968707 -5.145588 13.492311
H 5.558717 -5.071671 14.403694
C 4.123515 -4.096515 13.116218

H 4.056971 -3.198933 13.727450
C 3.370895 -4.192909 11.940900
H 2.732091 -3.368270 11.632288
Au 4.388362 -2.543504 9.138271
C 6.008188 -4.046597 6.431416
H 5.537993 -3.745746 5.488762
H 6.275383 -3.158992 7.009341
H 6.903015 -4.642011 6.226957
I 4.432787 -0.134707 10.266337

HF=-683.279163
Zero-point vibrational energy 540469.8 (Joules/Mol)
Zero-point correction= 0.205854 (Hartree/Particle)
Thermal correction to Energy= 0.221314
Thermal correction to Enthalpy= 0.222258
Thermal correction to Gibbs Free Energy= 0.157890
Sum of electronic and zero-point Energies= -683.073309
Sum of electronic and thermal Energies= -683.057849
Sum of electronic and thermal Enthalpies= -683.056905
Sum of electronic and thermal Free Energies= -683.121273

Transition State $\mathbf{4}_{\mathbf{TS}}$
Imaginary Frequency at -390.2 cm^{-1}
Cartesian Coordinates
Au 0.597803 2.466993 5.071967
Au -0.148862 0.982542 1.900719
N -2.200268 1.317247 4.741187
C -0.872516 1.163067 4.510130
N -0.734494 -0.005824 3.779271
C -2.005374 -0.592954 3.654171
C -2.914277 0.240270 4.222257
C -2.780898 2.614445 5.134396
C -2.417596 3.662741 4.095268
C -2.568388 3.389110 2.727892
C -2.124562 4.306779 1.773827
C -1.531580 5.509535 2.176727
C -1.381943 5.788200 3.539481
C -1.818519 4.863279 4.493629
N -0.012876 2.110719 -0.925681
C 0.389813 2.156072 0.369786
N 1.122858 3.294082 0.481642
C 1.161208 3.965059 -0.728152
C 0.444977 3.218906 -1.617582
C -0.906183 1.062792 -1.460292
C -2.302872 1.180909 -0.887286
C -2.787948 0.199243 -0.014200
C -4.050963 0.332696 0.571540
C -4.837463 1.453181 0.286345

C -4.361362 2.433786 -0.593641
C -3.100132 2.296364 -1.179208
H -2.140188 -1.529351 3.132719
H -3.989889 0.182785 4.309834
H -2.375952 2.893943 6.112119
H -3.862926 2.471556 5.225014
H -2.992177 2.441398 2.402610
H -2.236194 4.074937 0.718297
H -1.174782 6.218038 1.431792
H -0.904416 6.711309 3.861288
H -1.658491 5.054702 5.552542
H 1.698376 4.893975 -0.849513
H 0.237040 3.364739 -2.667162
H -0.468762 0.094287 -1.201115
H -0.903606 1.173150 -2.549215
H -2.161927 -0.658826 0.221955
H -4.412281 -0.430106 1.257321
H -5.816909 1.564358 0.747008
H -4.970363 3.307082 -0.817634
H -2.728964 3.065054 -1.855245
C 1.688283 3.789507 1.744934
H 2.503889 4.480311 1.518118
H 0.907537 4.295519 2.320374
H 2.060226 2.942950 2.326180
I 2.520744 4.173722 5.755218
C 0.957517 -1.135728 4.256240
H 0.896471 -1.688485 3.330204
H 1.538252 -0.225018 4.304534
H 0.476425 -1.513879 5.146755
I 3.208287 -2.548769 4.913336

HF=-1366.5178347

Zero-point vibrational energy 1077087.1 (Joules/Mol)

Zero-point correction= 0.410241 (Hartree/Particle)

Thermal correction to Energy= 0.442523

Thermal correction to Enthalpy= 0.443467

Thermal correction to Gibbs Free Energy= 0.337941

Sum of electronic and zero-point Energies= -1366.107594

Sum of electronic and thermal Energies= -1366.075312

Sum of electronic and thermal Enthalpies= -1366.074367

Sum of electronic and thermal Free Energies= -1366.189894

Compound **5**

Cartesian Coordinates

N 3.878842 -5.287332 7.773446
N 2.723881 -4.170239 6.340201
C 5.272471 1.327209 10.273034
H 5.118417 1.945659 11.144899
C 3.610884 -4.018190 7.358119
C 3.182483 -6.213855 7.021097

H 3.270229 -7.275771 7.196165
C 2.456534 -5.507084 6.110248
H 1.788858 -5.831728 5.326682
C 4.848670 -5.608312 8.831505
H 5.833922 -5.275337 8.489421
H 4.862243 -6.700163 8.920890
C 4.503992 -4.958581 10.154025
C 5.470035 -4.199211 10.825770
H 6.446822 -4.060532 10.367565
C 5.168522 -3.596385 12.051432
H 5.923037 -3.000099 12.560806
C 3.893025 -3.739169 12.606936
H 3.652527 -3.261348 13.554677
C 2.918795 -4.485305 11.930425
H 1.922287 -4.592440 12.354779
C 3.224778 -5.094695 10.710802
H 2.464869 -5.666215 10.180477
Au 4.353439 -2.270501 8.151776
C 5.013090 -0.493170 8.952605
N 5.856870 0.398672 8.364282
N 4.664686 0.090173 10.130671
C 6.026930 1.521516 9.154861
H 6.667574 2.338938 8.858289
C 3.704346 -0.472665 11.082357
H 3.615590 -1.543259 10.892028
H 4.071157 -0.311523 12.100271
H 2.729645 0.012403 10.957042
C 6.471977 0.180766 7.047665
H 7.090827 -0.720928 7.108676
H 7.129055 1.038253 6.861700
C 2.254911 -3.081962 5.484569
H 2.365116 -2.138037 6.022501
H 2.867692 -3.055009 4.578013
H 1.204525 -3.251069 5.228419
C 5.454729 0.022831 5.935235
C 5.767992 -0.809150 4.854025
C 4.219928 0.685624 5.956451
C 4.863836 -0.974903 3.801458
H 6.713410 -1.347888 4.848870
C 3.314564 0.521994 4.904546
H 3.958429 1.313881 6.805060
C 3.636194 -0.306438 3.822044
H 5.108995 -1.651916 2.986977
H 2.355220 1.035362 4.933147
H 2.925100 -0.444722 3.009907
Au 6.509539 -4.097952 5.776268
I 5.242270 -4.935027 3.597237
I 8.156841 -3.424374 7.765451

HF=-1366.6065089

Zero-point vibrational energy 1085538.7 (Joules/Mol)

Zero-point correction= 0.413460 (Hartree/Particle)

Thermal correction to Energy= 0.445945

Thermal correction to Enthalpy= 0.446889

Thermal correction to Gibbs Free Energy= 0.341884

Sum of electronic and zero-point Energies= -1366.193049

Sum of electronic and thermal Energies= -1366.160564

Sum of electronic and thermal Enthalpies= -1366.159620

Sum of electronic and thermal Free Energies= -1366.264625

Adduct between two molecules of **4**

Cartesian Coordinates

N -2.660267 12.944409 18.884830
N -2.294043 10.819340 18.821942
C -1.920098 11.959604 19.470846
C -3.492642 12.427020 17.906976
C -3.263990 11.087697 17.872595
C -2.567656 14.375661 19.176720
H -1.733760 14.540473 19.861991
H -2.387369 14.921884 18.245388
C -1.865814 9.438871 19.131687
H -2.076188 8.854794 18.228722
H -2.506730 9.063198 19.935863
C -1.815702 10.609006 23.794821
C -0.243501 10.141658 25.359029
C -0.472407 11.480944 25.393532
N -1.442191 11.749215 24.443981
N -1.075527 9.624263 24.380872
C -1.870353 13.129651 24.133998
H -1.229319 13.505204 23.329873
H -1.660119 13.713844 25.036917
C -1.167907 8.192987 24.089066
H -2.000729 8.028177 23.402502
H -0.234832 7.844448 23.635049
Au -0.460777 12.209708 20.881590
Au -3.275051 10.358702 22.384159
I -5.374920 9.860659 20.815273
I 1.638859 12.707494 22.450848
H -4.155597 13.052512 17.327676
H -3.699795 10.306175 17.268857
H -0.036893 12.262473 25.997478
H 0.419431 9.516203 25.938404
H -3.500046 14.723998 19.632310
H -1.349801 7.646911 25.020179
C -3.321089 13.250845 23.748760
C -4.340410 12.889951 24.640823
C -3.653999 13.704301 22.467876
C -5.677460 12.973493 24.247702
H -4.082629 12.521003 25.632105
C -4.991446 13.796126 22.075070
H -2.850920 13.940610 21.776731
C -6.005279 13.427317 22.963442
H -6.465129 12.681261 24.939303
H -5.243928 14.127022 21.069896
H -7.047003 13.480203 22.654282
C -0.415007 9.317583 19.516665
C -0.081866 8.864307 20.797560
C 0.604176 9.678346 18.624355
C 1.255648 8.772421 21.190105
H -0.884845 8.628172 21.488876
C 1.941294 9.594743 19.017247
H 0.346200 10.047185 17.633072
C 2.269314 9.141049 20.301498
H 1.508272 8.441620 22.195257
H 2.728848 9.886846 18.325460
H 3.311088 9.088118 20.610440

HF=-1366.6055784

Zero-point vibrational energy 1085559.2 (Joules/Mol)

Zero-point correction= 0.413468 (Hartree/Particle)

Thermal correction to Energy= 0.445998

Thermal correction to Enthalpy= 0.446943

Thermal correction to Gibbs Free Energy= 0.343003

Sum of electronic and zero-point Energies= -1366.192111

Sum of electronic and thermal Energies= -1366.159580

Sum of electronic and thermal Enthalpies= -1366.158636

Sum of electronic and thermal Free Energies= -1366.262575

Compound **CTC^{Me}** in iodomethane solution

Cartesian Coordinates

C 4.068288 10.253720 1.745247
C 4.966916 12.254239 2.262589
H 5.595425 13.124086 2.133728
C 4.086190 11.902109 3.247786
H 3.808345 12.428478 4.150287
C 0.766724 8.298622 4.853119
C -0.743737 7.352268 6.231507
H -1.380641 7.270018 7.100949
C -0.539284 6.518012 5.167310
H -0.980685 5.556308 4.945689
C 2.030949 5.867888 0.744110
C 2.653606 4.810762 -1.145862
H 2.667516 4.021060 -1.883781
C 3.324481 6.000008 -1.068265
H 4.043791 6.436686 -1.747014
Au 3.591679 8.509857 0.840177
Au 2.111541 9.545708 4.001960
Au 1.159628 6.365678 2.499882
N 4.945538 11.224113 1.339079
N 3.535836 10.673157 2.927486
N 0.071239 8.450830 6.023377
N 0.388696 7.101742 4.322271
N 1.856243 4.742730 -0.017116
N 2.940165 6.647001 0.093287
C 0.969652 3.627099 0.303893
H 1.552246 2.704946 0.412380
H 0.463008 3.852245 1.246094
H 0.226177 3.499121 -0.491196
C 0.164632 9.594879 6.926752
H 0.879123 10.306935 6.505349
H -0.815985 10.074466 7.025589
H 0.512753 9.266038 7.912712
C 5.745417 11.191664 0.117258
H 6.812086 11.226056 0.367078
H 5.523815 10.261612 -0.412849
H 5.490724 12.046557 -0.519659

HF=-1202.8411966

Zero-point vibrational energy 688383.6 (Joules/Mol)

Zero-point correction= 0.262191 (Hartree/Particle)

Thermal correction to Energy= 0.284911

Thermal correction to Enthalpy= 0.285855

Thermal correction to Gibbs Free Energy= 0.203121

Sum of electronic and zero-point Energies= -1202.579005

Sum of electronic and thermal Energies= -1202.556286

Sum of electronic and thermal Enthalpies= -1202.555341

Sum of electronic and thermal Free Energies= -1202.638076

Compound $\mathbf{10^{Me}_{TS}}$

Imaginary frequency -421.6 cm^{-1}

Cartesian Coordinates

C 3.370087 10.554086 2.992277
C 3.141510 12.678399 3.721094
H 3.162179 13.755135 3.629756
C 2.850723 11.866079 4.763855
H 2.566417 12.101100 5.779281
C 0.089709 7.561468 5.023574
C -1.579089 6.242427 5.748021
H -2.423275 5.952498 6.357450
C -0.929708 5.606892 4.725570
H -1.119229 4.641536 4.277601
C 2.890867 5.986342 1.276552
C 4.246425 5.308170 -0.390286
H 4.662330 4.622432 -1.114778
C 4.537967 6.610848 -0.095797
H 5.273598 7.273078 -0.530251
Au 3.627197 8.884475 1.901317
Au 1.409189 9.066946 4.790566
Au 1.486029 6.077898 2.727729
N 3.458032 11.850479 2.639234
N 2.982994 10.530641 4.337899
N -0.937001 7.455414 5.920714
N 0.097255 6.421616 4.281510
N 3.226392 4.935535 0.467457
N 3.703840 7.024968 0.928779
C 2.602949 3.613995 0.485751
H 3.366064 2.846187 0.654795
H 1.872738 3.589812 1.298858
C -1.289103 8.444025 6.937697
H -2.355583 8.683154 6.865430
H -0.697232 9.345851 6.760352
C 3.843346 12.349136 1.317268
H 4.744479 12.964617 1.408849
H 4.041853 11.490150 0.672323
I 6.692196 8.311223 6.648779
C 4.504404 9.635811 5.297445
H 4.423510 8.706569 4.750559
H 4.059668 9.708574 6.280320
H 5.248854 10.361122 4.997163
H 3.025879 12.947885 0.902066
H -1.066946 8.051261 7.936623
H 2.096585 3.424766 -0.467804

HF=-1254.1710488

Zero-point vibrational energy 787049.5 (Joules/Mol)

Zero-point correction= 0.299771 (Hartree/Particle)

Thermal correction to Energy= 0.327332

Thermal correction to Enthalpy= 0.328276

Thermal correction to Gibbs Free Energy= 0.234390

Sum of electronic and zero-point Energies= -1253.871277

Sum of electronic and thermal Energies= -1253.843717

Sum of electronic and thermal Enthalpies= -1253.842772

Sum of electronic and thermal Free Energies= -1253.930956

Compound **11**^{Me}

Cartesian Coordinates

C 3.323617 10.568418 1.851020

C 2.808222 12.723219 1.481060

H 2.794846 13.664679 0.950394

C 2.254969 12.336824 2.671041

H 1.660085 12.900349 3.375983

C 1.321190 8.177347 5.591231

C 0.639019 6.899923 7.312793

H 0.343760 6.675009 8.328049

C 0.749713 6.114753 6.196865

H 0.562306 5.056864 6.074040

C 1.965879 5.917077 1.190077

C 2.465014 6.184907 -0.921185

H 2.541174 6.686557 -1.880158

C 2.855390 4.903719 -0.603641

H 3.304496 4.107784 -1.183442

Au 4.120617 8.703380 1.689877

Au 1.925060 9.687178 4.402113

Au 1.546586 6.327315 3.122032

N 3.473426 11.615751 0.984787

N 2.577841 11.009734 2.895410

N 0.999024 8.177388 6.921860

N 1.169325 6.903652 5.140357

N 1.910354 6.802050 0.178731

N 2.524040 4.736647 0.725540

C 0.999427 9.350138 7.791886

H 1.442796 10.183079 7.239276

H -0.026410 9.607889 8.080937

H 1.592798 9.146373 8.690171

C 4.168424 11.565803 -0.300043

H 4.757812 12.479238 -0.429354

H 4.831408 10.696311 -0.300752

H 3.445719 11.474059 -1.119434

I 5.473687 6.353280 1.960881

C 4.850098 6.193101 4.078142

H 4.217220 5.307587 4.129836

H 5.764662 6.112212 4.666935

H 4.283528 7.103311 4.273949

C 2.804565 3.539501 1.505877

H 2.459233 2.650280 0.965660
H 3.881442 3.447885 1.701069
H 2.272039 3.615328 2.458629

HF=-1254.1742508

Zero-point vibrational energy 784362.2 (Joules/Mol)

Zero-point correction= 0.298748 (Hartree/Particle)

Thermal correction to Energy= 0.327070

Thermal correction to Enthalpy= 0.328014

Thermal correction to Gibbs Free Energy= 0.233413

Sum of electronic and zero-point Energies= -1253.875503

Sum of electronic and thermal Energies= -1253.847181

Sum of electronic and thermal Enthalpies= -1253.846237

Sum of electronic and thermal Free Energies= -1253.940838

Compound **12**$^{\mathbf{Me}}$

Cartesian Coordinates

C 4.469980 9.969286 2.054398
C 5.721192 11.747746 2.629040
H 6.461924 12.524475 2.500705
C 4.905031 11.428017 3.680797
H 4.813425 11.894455 4.651934
C 1.297341 7.888978 5.018107
C -0.514250 6.877179 5.889643
H -1.419599 6.789225 6.473780
C 0.168579 5.973680 5.119869
H -0.042100 4.930952 4.926843
C 2.053644 5.826855 1.621730
C 0.808147 5.383347 -0.177593
H 0.066054 4.866858 -0.769666
C 1.620300 6.448790 -0.451845
H 1.708804 7.041549 -1.351100
Au 3.591643 8.393520 1.137466
Au 2.675785 9.218347 4.390448
Au 2.933465 5.681168 3.482527
N 5.438577 10.833950 1.628695
N 4.136321 10.335522 3.325605
N 0.198870 8.060796 5.817012
N 1.273939 6.604757 4.586769
N 1.097841 4.998985 1.122403
N 2.398561 6.710938 0.665598
C 0.430319 3.923053 1.854563
H 0.191696 3.113977 1.158616
H 1.106784 3.550299 2.628381
H -0.489466 4.295337 2.319541
C -0.185953 9.311914 6.464510
H 0.634160 10.024198 6.339647
H -1.094770 9.715433 6.002244
H -0.364000 9.136973 7.531280
C 6.087716 10.794559 0.321198
H 7.161448 10.609471 0.440517
H 5.637353 9.982090 -0.255771
H 5.935441 11.745896 -0.201209

I 4.320560 5.546370 5.839126
C 4.555732 4.879077 2.387155
H 4.187709 4.115411 1.693899
H 4.978527 5.722407 1.827813
H 5.283369 4.458263 3.085723

HF=-1254.2017774

Zero-point vibrational energy 786112.2 (Joules/Mol)

Zero-point correction= 0.299414 (Hartree/Particle)

Thermal correction to Energy= 0.327461

Thermal correction to Enthalpy= 0.328405

Thermal correction to Gibbs Free Energy= 0.234814

Sum of electronic and zero-point Energies= -1253.902363

Sum of electronic and thermal Energies= -1253.874317

Sum of electronic and thermal Enthalpies= -1253.873372

Sum of electronic and thermal Free Energies= -1253.966964

Compound **3**

Cartesian Coordinates

C 4.052124 10.289342 1.769221
C 4.933083 12.297235 2.290620
H 5.558390 13.169896 2.166509
C 4.032265 11.952978 3.260986
H 3.728675 12.485287 4.151459
C 0.754943 8.314959 4.889688
C -0.702913 7.406366 6.314459
H -1.334095 7.351872 7.189756
C -0.479413 6.530023 5.288352
H -0.890902 5.546164 5.113851
C 2.045300 5.878091 0.810470
C 2.651076 4.830528 -1.089255
H 2.662160 4.042334 -1.828819
C 3.309941 6.026911 -1.019776
H 4.014236 6.472481 -1.708285
Au 3.577711 8.537755 0.880725
Au 2.047093 9.588655 3.982619
Au 1.189150 6.364688 2.574812
N 4.936334 11.257670 1.377298
N 3.497478 10.723997 2.930094
N 0.087272 8.515428 6.055591
N 0.429163 7.099506 4.408855
N 1.872043 4.751661 0.051455
N 2.935778 6.667915 0.148300
C 0.992676 3.631538 0.377348
H 1.573331 2.703226 0.423724
H 0.535328 3.826513 1.350954
H 0.208955 3.536143 -0.383021
C 0.117750 9.748796 6.839775
H 1.117147 10.186729 6.767198
H -0.619070 10.461285 6.451591
H -0.106737 9.511726 7.883285

C 5.746817 11.221451 0.162125

H 6.805467 11.342547 0.417322

H 5.593593 10.253075 -0.321200

H 5.439006 12.023531 -0.518607

I 0.047586 10.325134 2.143644

C 3.576486 8.949157 5.320286

H 3.148597 8.413231 6.171303

H 4.219707 8.294579 4.721620

H 4.111769 9.851100 5.635863

HF=-1254.2147396

Zero-point vibrational energy 785715.7 (Joules/Mol)

Zero-point correction= 0.299263 (Hartree/Particle)

Thermal correction to Energy= 0.327458

Thermal correction to Enthalpy= 0.328402

Thermal correction to Gibbs Free Energy= 0.234015

Sum of electronic and zero-point Energies= -1253.915476

Sum of electronic and thermal Energies= -1253.887282

Sum of electronic and thermal Enthalpies= -1253.886338

Sum of electronic and thermal Free Energies= -1253.983724

Compound **HCl**

Cartesian Coordinates

H 0.000000 0.000000 0.004528

Cl 0.000000 0.000000 1.295472

HF=-460.8463191

Zero-point vibrational energy 17047.5 (Joules/Mol)

Zero-point correction= 0.006493 (Hartree/Particle)

Thermal correction to Energy= 0.008854

Thermal correction to Enthalpy= 0.009798

Thermal correction to Gibbs Free Energy= -0.011402

Sum of electronic and zero-point Energies= -460.839826

Sum of electronic and thermal Energies= -460.837466

Sum of electronic and thermal Enthalpies= -460.836521

Sum of electronic and thermal Free Energies= -460.857721

Transition State for the interaction of **CTC^{Bz}** and **HCl**

Imaginary Frequency at -665.4 cm^{-1}

Cartesian Coordinates

C 3.067100 10.870700 2.663000
C 2.892800 13.085600 3.018600
H 3.092500 14.135100 2.855200
C 2.090200 12.433800 3.913300
H 1.449300 12.825600 4.690300
C 0.450000 7.802500 5.495700
C -0.798700 6.358000 6.696200
H -1.494800 6.049500 7.463200
C -0.111500 5.654400 5.746800
H -0.109000 4.596100 5.526400
C 3.107200 6.067300 1.758700
C 4.099200 5.587900 -0.213700
H 4.235000 5.041400 -1.136100
C 4.782100 6.619500 0.336000
H 5.646200 7.164700 -0.015000
Au 3.653700 9.055600 2.008700
Au 1.262700 9.481500 4.713900
Au 1.893500 6.167100 3.360800
N 3.495800 12.103800 2.250700
N 2.201600 11.071900 3.691900
N -0.443000 7.685600 6.527100
N 0.654200 6.545000 5.013000
N 3.072800 5.260500 0.677800
N 4.177400 6.940300 1.566700
C 2.077300 4.193000 0.419900
H 1.179900 4.657500 -0.003600
H 2.520700 3.544500 -0.343700
C -0.907600 8.794000 7.379200
H -0.097800 9.078500 8.061100
H -1.733300 8.396200 7.980600
C 4.395900 12.373300 1.113000
H 3.826900 12.273800 0.181300
H 4.706100 13.420100 1.210700
C -1.353600 9.998800 6.578300
C -0.787000 11.254700 6.828900
C -2.283300 9.860600 5.538300
C -1.138500 12.360800 6.047500
H -0.044900 11.357000 7.618400
C -2.628600 10.960600 4.750200
H -2.706500 8.881100 5.323600
C -2.054200 12.214100 5.001000
H -0.684900 13.329900 6.245800
H -3.339800 10.840900 3.935300
H -2.316700 13.068600 4.380500
C 5.597100 11.453800 1.082600
C 6.418200 11.313300 2.210500
C 5.853900 10.677000 -0.053900
C 7.467400 10.392700 2.208200
H 6.206600 11.898000 3.103800
C 6.913900 9.763500 -0.063600
H 5.200900 10.766700 -0.920200
C 7.716600 9.612600 1.071100
H 8.086200 10.274600 3.095300
H 7.099900 9.159700 -0.949800
H 8.527200 8.886800 1.074800
C 1.725900 3.409100 1.663100
C 0.401300 3.370500 2.115000
C 2.729800 2.783100 2.415400
C 0.080900 2.721400 3.312300
H -0.373000 3.879100 1.543900
C 2.414200 2.145400 3.616300
H 3.763900 2.832100 2.079600

C 1.088400 2.116100 4.069400
H -0.949600 2.707100 3.660900
H 3.201300 1.676800 4.203600
H 0.844800 1.626800 5.010300
Cl 5.894000 6.658100 3.851200
H 4.987500 6.843200 2.629400

HF=-2356.5498305
Zero-point vibrational energy 1327670.2 (Joules/Mol)
Zero-point correction= 0.505683 (Hartree/Particle)
Thermal correction to Energy= 0.544418
Thermal correction to Enthalpy= 0.545362
Thermal correction to Gibbs Free Energy= 0.425182
Sum of electronic and zero-point Energies= -2356.044148
Sum of electronic and thermal Energies= -2356.005413
Sum of electronic and thermal Enthalpies= -2356.004469
Sum of electronic and thermal Free Energies= -2356.136904

Transition State for the interaction of **CTC^{Me}** and **HCl**
Imaginary Frequency at -693.9 cm^{-1}
Cartesian Coordinates
C 2.919667 10.875668 2.549696
C 2.530357 13.080562 2.734610
H 2.631755 14.130586 2.499479
C 1.770800 12.421275 3.661828
H 1.073192 12.810106 4.390253
C 0.495862 7.782064 5.534524
C -0.521491 6.310951 6.904267
H -1.136296 5.986171 7.731707
C 0.181352 5.613656 5.961326
H 0.285812 4.546848 5.821530
C 3.210317 6.066888 1.828792
C 4.139730 5.623264 -0.179835
H 4.252160 5.090516 -1.113428
C 4.828717 6.656754 0.359438
H 5.674299 7.213159 -0.018127
Au 3.632082 9.076346 1.992342
Au 1.170909 9.467110 4.643400
Au 2.028737 6.150136 3.453455
N 3.235714 12.107997 2.048927
N 2.012636 11.063301 3.545301
N -0.318681 7.651219 6.626166
N 0.805517 6.519930 5.121208
N 3.146881 5.275598 0.740149
N 4.258105 6.962230 1.610965
C 2.183908 4.192380 0.531042
H 2.722471 3.255653 0.353966
H 1.567767 4.105558 1.428931
C -0.875979 8.752571 7.408330
H -1.963793 8.643053 7.478436
H -0.632999 9.690564 6.902189
C 4.212933 12.375131 0.995346
H 3.789014 13.082711 0.275285

H 5.129521 12.793614 1.427289

Cl 6.024948 6.853108 3.871483

H 5.085401 6.934442 2.650728

H 4.444479 11.432139 0.493076

H -0.441942 8.756381 8.414951

H 1.552938 4.426158 -0.333127

HF=-1663.6931792

Zero-point vibrational energy 704912.1 (Joules/Mol)

Zero-point correction= 0.268487 (Hartree/Particle)

Thermal correction to Energy= 0.294045

Thermal correction to Enthalpy= 0.294989

Thermal correction to Gibbs Free Energy= 0.206920

Sum of electronic and zero-point Energies= -1663.424692

Sum of electronic and thermal Energies= -1663.399135

Sum of electronic and thermal Enthalpies= -1663.398190

Sum of electronic and thermal Free Energies= -1663.482260

5. Crystallographic files

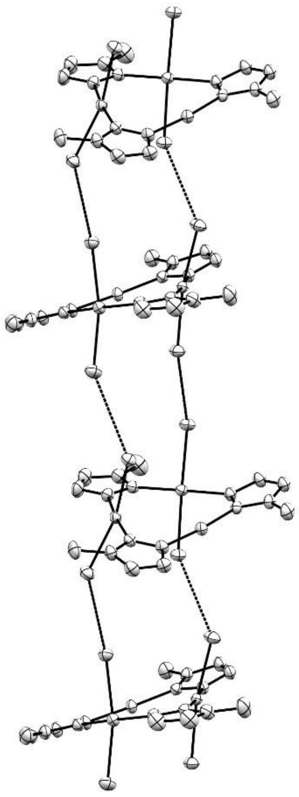

Figure S17. ORTEP plot of the columnar packing in the crystal structure of the compound **2** corresponding to the platelets phase. The linear arrangement is based on the intermolecular iodine-iodine interactions.

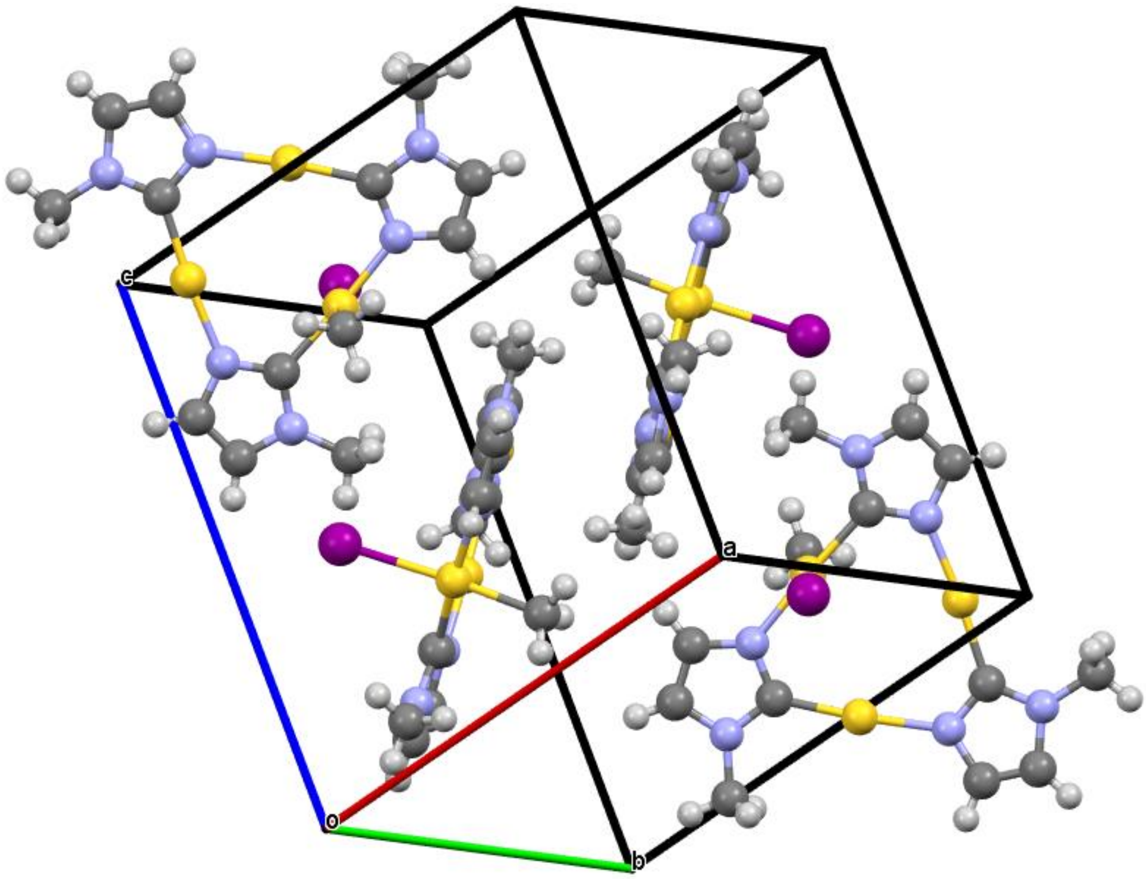

Figure S18. Packing of compound **3** in the unit cell displaying neither aurophilic nor halogen contacts.

References

(1) Abdou, H. E.; Mohamed, A. A.; Fackler, J. P. Oxidative Addition of Methyl Iodide to Dinuclear Gold(I) Amidinate Complex: Schmidbaur's Breakthrough Reaction Revisited with Amidinates. *Zeitschrift fur Naturforsch. - Sect. B J. Chem. Sci.* **2004**, *59* (11–12), 1480–1482. https://doi.org/10.1515/znb-2004-11-1217.

(2) Muramatsu, S.; Wu, X.; Chen, M.; Zhou, M.; Tsukuda, T. Photoassisted Homocoupling of Methyl Iodide Mediated by Atomic Gold in Low-Temperature Neon Matrix. *J. Phys. Chem. A* **2017**, *121* (44), 8408–8413. https://doi.org/10.1021/acs.jpca.7b08863.
